# Mass Spectral Analysis of Sterols and Other Steroids in Different Ionization Modes: Sensitivity and Oxidation Artifacts

**DOI:** 10.1021/jasms.5c00099

**Published:** 2025-06-27

**Authors:** Kevin D. McCarty, F. Peter Guengerich

**Affiliations:** † Department of Biochemistry, 12327 Vanderbilt University School of Medicine, Nashville, Tennessee 37232-0146, United States

**Keywords:** mass spectrometry, APCI, HESI, steroids, hydroxy steroids, oxidation, ammonium fluoride, artifacts

## Abstract

In the course of synthetic work and mass spectrometry (MS) with hydroxy steroids, we observed not only the loss of H_2_O but prominent 2 and 4 amu losses using atmospheric pressure chemical ionization (APCI), leading to confusion of the structural assignments. This loss of 2 amu, which we attributed mainly to oxidation of hydroxyls, varied among 44 steroids and sterols analyzed; 36 showed losses of 2*n* amu in APCI MS analysis (17/22 Δ^5^ steroids, 17/19 Δ^4^ steroids, and 2/3 estrogens). With the Δ^5^ steroids and sterols, the precursor MH^+^ was either observed as a minor ion or (more frequently) not detected at all, constituting the base peak in 7/22 cases. With heated electrospray ionization (HESI) MS, 2*n* amu losses were detected (generally weakly) in 9/44 cases but constituted the base peak in 3/9. In general, the sensitivity (base peak intensity) of steroids correlated with conjugation of the of the steroid frame. Δ^4^ steroids generally performed best on HESI^+^ (up to a maximum factor of 8-fold), while Δ^5^ steroids and sterols performed better on APCI^+^ (up to >137-fold), except for two trihydroxypregnanes. Estrogens did not show a clear trend. Sensitivity generally increased with the use of NH_4_F as a mobile phase additive in ESI^+^ (up to a maximum of 7-fold). We conclude that the prominence of 2*n* amu losses is variable among steroids and sterols but is more commonly an artifact of APCI MS. These *m*/*z* losses can constitute dominant ions that impede detection of the precursor MH^+^ and complicate structural assignments.

## Graphical abstract

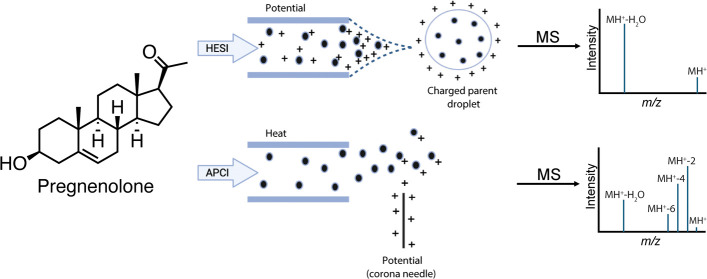

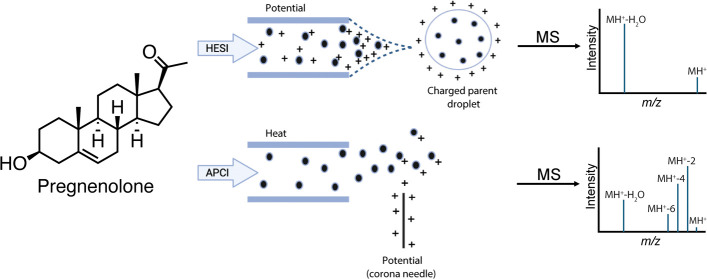

## Introduction

The introduction of electrospray ionization (ESI) technology revolutionized mass spectrometry (MS) in terms of coupling to HPLC and in the generation of mass spectra of intact molecules.^1^ The approach is excellent for both positively and negatively charged molecules, but uncharged molecules (e.g., alkanes, retinoids, and sterols) can be problematic. In many cases, atmospheric pressure chemical ionization (APCI) MS adds considerable sensitivity, but some artifacts have been reported. These include water and oxygen adducts, demethylation, decarboxylation, and dehydrogenation,^2^ some of which were inherent in the electron impact MS of the past.^3^ Several examples of problems with APCI have been documented.^4–6^ Limited reports of oxidation have appeared,^7^ e.g., readily oxidizable compounds derived from hair dyes.^8^ Very recently, the presence of oxygen addition artifacts has also been noted.^9^

In the course of our work on the synthesis of some sterols, we frequently observed the loss of H_2_O and esters (acetoxy and formyl groups) in APCI MS.^10–12^ However, in the synthesis of some other steroids, we often observed products either 2, 4, or (in some cases) up to 6 amu less than expected (generally in terms of the precursor ion, MH^+^). We have developed reliable derivatization procedures for steroids containing carbonyls and used positive-ion ESI methods successfully for quantification in several cases.^11,13–16^ However, in the syntheses of (underivatized) steroids, we were often unsure (due to ≥2 amu losses from the MH^+^ precursor ion in LC-APCI-MS sample analysis, Scheme 1) as to the results of simple oxidations and reductions (e.g., NaBH_4_ and LiAlH_4_ reduction,^17^ Dess–Martin oxidation^18^) and had to turn to ^1^H and ^13^C NMR analysis for definitive answers.

**Scheme 1:**
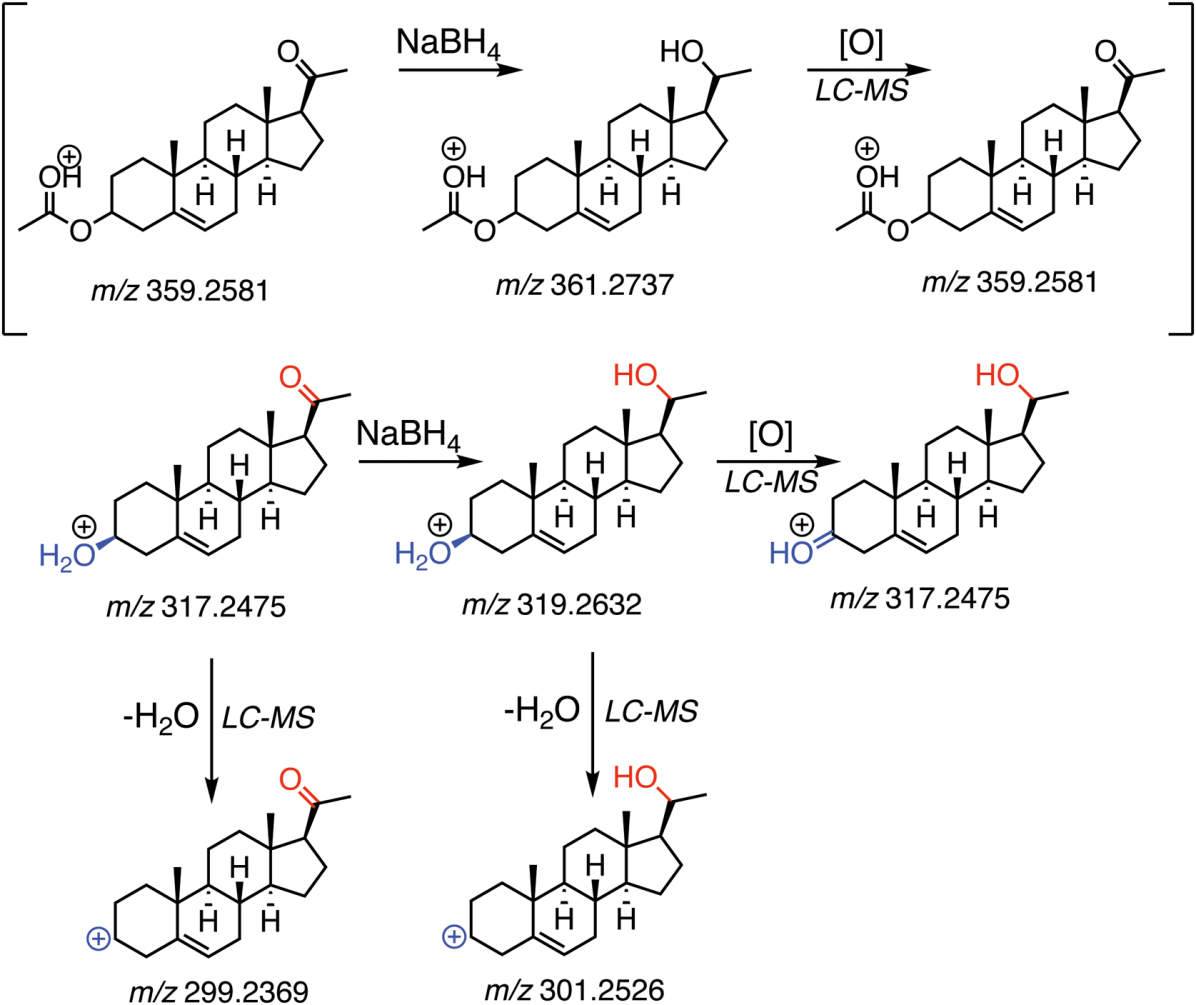
Complications in Correct Product Ion Characterization in Cases of In-Source Oxidation^a^

a In this example, the C-20 (red) carbonyl group of pregnenolone is reduced with NaBH_4_ to yield a +2 amu product. Upon LC-MS analysis, the C-3 (blue) hydroxyl moiety is oxidized to a carbonyl, resulting in a loss of 2 amu and a reduction product that is detected with the same *m/z* as the starting material (pregnenolone). When the C-3 hydroxyl moiety is instead lost as H_2_O (i.e., preventing the oxidation to a carbonyl), the +2 amu *m/z* difference between the starting material and the product is revealed. In this way, the MH^+^–H_2_O ions of the starting material and product confirm a successful reaction, while the corresponding MH^+^ ions are indistinguishable. All molecules are presented in their ionized form. The example ions for 3-acetoxypregnenolone, our synthetic intermediate, are shown above in brackets. Note: NaBH_4_ reductions yield epimeric alcohols which may separate on reversed phase HPLC, yielding two peaks (diastereomers) bearing identical MH^+^–2 ions.

We analyzed a number of hydroxy (OH) and keto steroids and sterols using APCI-MS and observed losses of 2 and 4 amu in many cases, as well as −18 (loss of H_2_O). This phenomenon has apparently not been reported for steroids previously, although LC-APCI-MS analyses of steroid libraries have been presented.^19,20^ We have now compiled the results of analysis of a series of steroids along with a comparison of fragmentation and sensitivity using heated ESI (HESI) as an alternative approach, employing NH_4_F to assist ionization.

## Experimental Section

### Chemicals

Most of the steroids (Scheme 2) were purchased from Sigma-Aldrich-Millipore or Steraloids. Compounds **14** ^11^ and **42** and **41** (FFMAS, follicular fluid meiosis activating sterol, (4*β*,5*α*)-4,4-dimethyl-cholestra-8,14,24-trien-3-ol) ^10^ were synthesized as descrived previously.

**Scheme 2:**
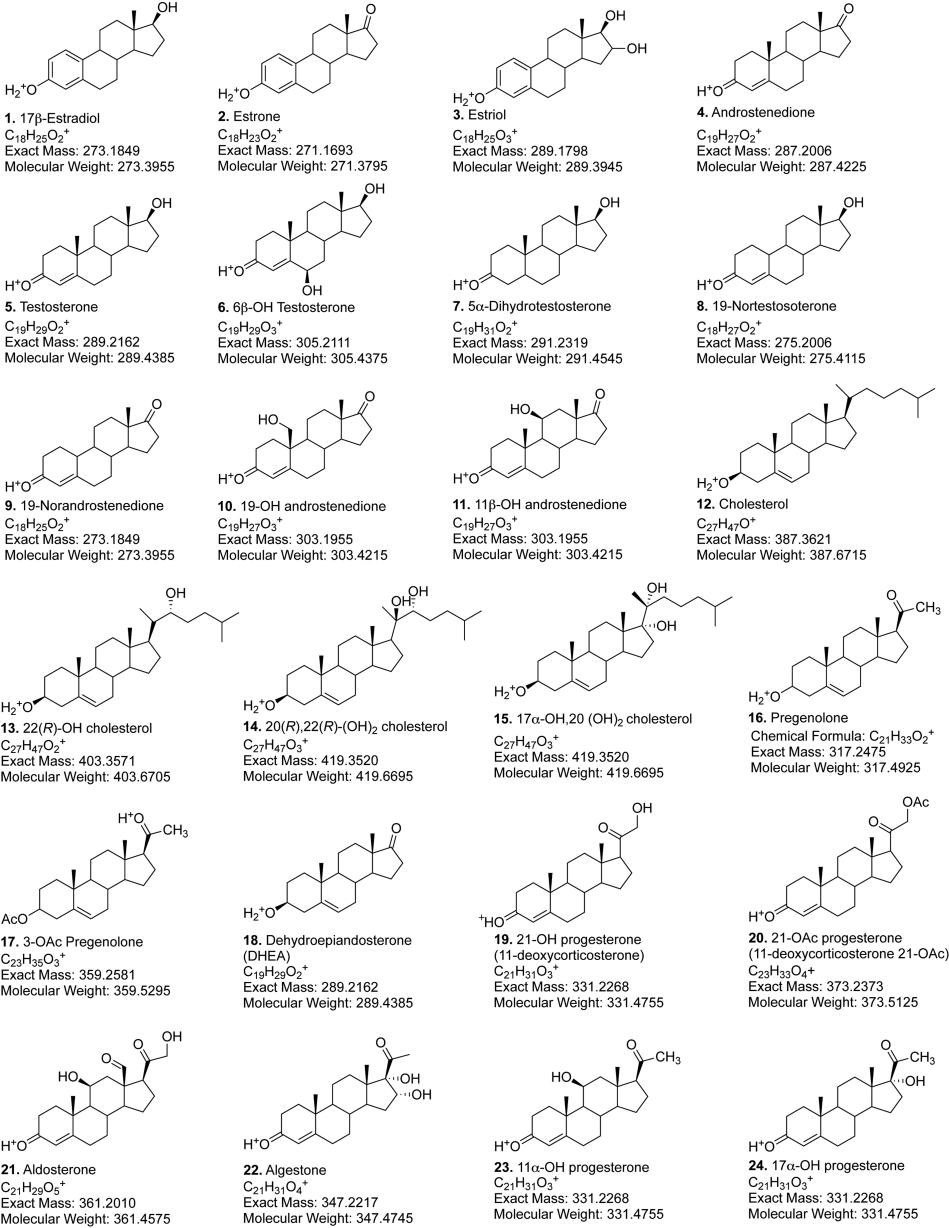

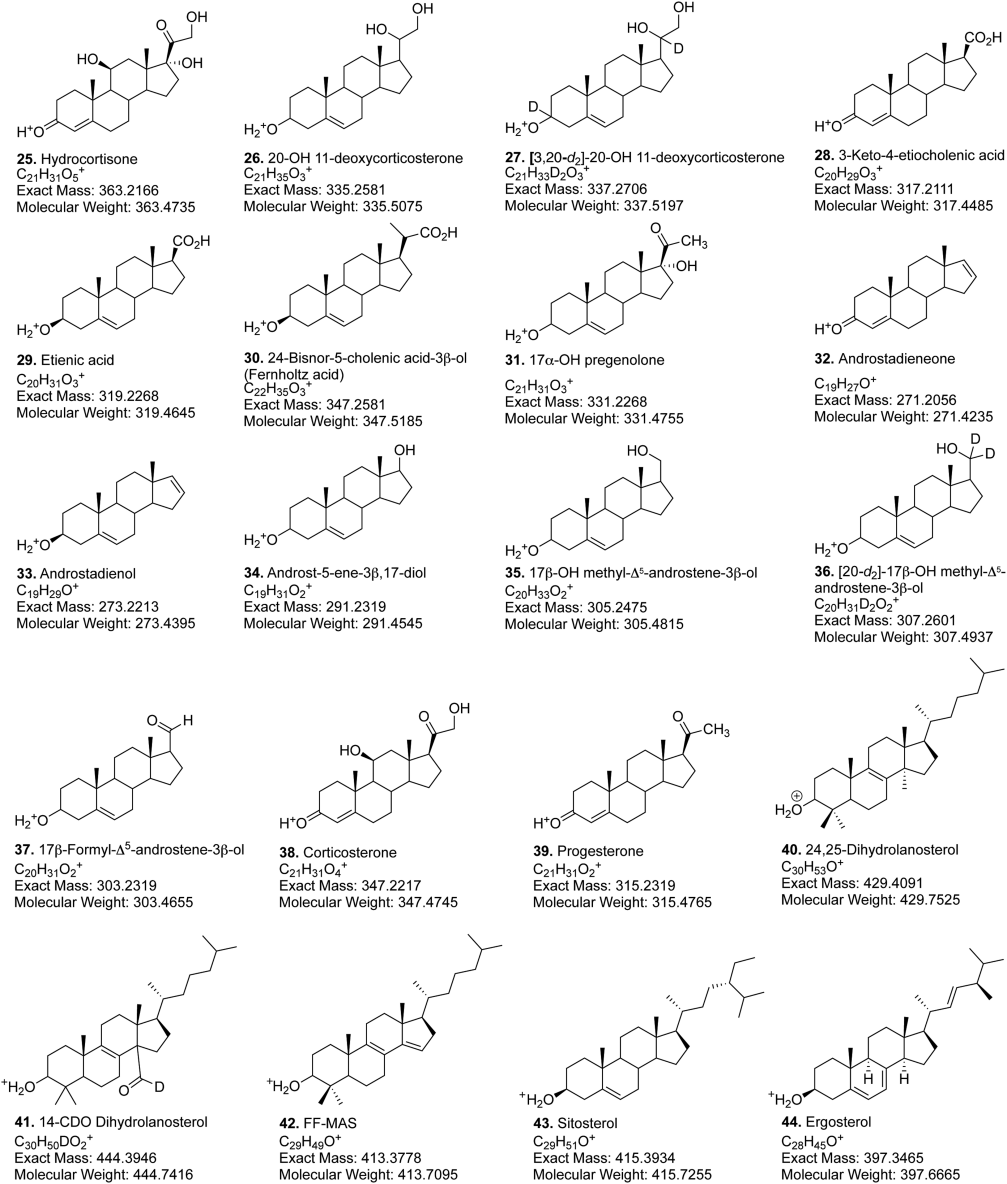
Structures and Names of Steroids Used in Analysis^a^

a All steroids are shown in their ionized form (MH^+^).

17α,20-(OH)_2_ cholesterol (**15**) was from Santa Cruz Biotechnology (a gift from N. Sharifi, Univ. Miami). Etienic acid (**29**) was converted to its ethyl ester by heating in C_2_H_5_OH in the presence of H_2_SO_4_ under reflux overnight and then reduced with LiAlH_4_ or LiAlD_4_ in (C_2_H_5_)_2_O (2 h, reflux) to give 17β-OH methyl-Δ^5^-androstene-3β-ol (**35**). 11-Deoxycorticosterone (19) was reduced to pregn-5-ene-3β,20,21-triol (**26**) by treatment with NaBH_4_ or NaBD_4_ in CH_3_OH at room temperature (2 h, 23 °C): ^1^H NMR (400 MHz, CD_3_OD) δ 5.27, 4.08, 3.55, 3.34; ^13^C NMR (100 MHz, CD_3_OD) δ 147.5, 124.5, 75.5, 68.1, 64.6, 56.5, 53.1. This 3β,20,21-triol was treated with HIO_4_ ^21,22^ to yield 17β-formyl-Δ^5^-androstene-3-ol (**37**): ^1^H NMR (400 MHz, CDCl_3_) δ 9.77, 5.37, 2.28, 1.12, 1.01; ^13^C NMR (400 MHz, CDCl_3_) δ 205.1, 146.5, 122.6.

### UPLC-MS

Steroids (Scheme 1, dissolved in CH_3_CN) were injected (10 μL, held at 25 °C) using a Waters Acquity UPLC and separated (flow rate 0.2 mL min^–1^) using a 2.1 mm × 100 mm (1.7 μm) Acquity BEH octadecylsilane (C_18_) column (held at 25 °C) with a gradient mobile phase of 0.1% HCO_2_H dissolved in (A) H_2_O and (B) CH_3_CN as follows (expressed as % B, v/v): 0 min, 70%; 0.5 min, 70%; 3 min, 100%; 8 min, 100%; 8.1 min, 70%; 10 min, 70%. For allsterols , this was modified to sample injection in 100% B at a flow rate of 0.6 mL min^–1^ for 10 min. Column eluates were subjected to APCI ionization (positive ion mode unless noted otherwise) in a Thermo Fisher Scientific LTQ XL Orbitrap mass spectrometer in the Vanderbilt Mass Spectrometry Research Core Facility, with a vaporizer temperature of 350 °C, a resolution setting of 60,000, and scanning from *m*/*z* 100–800. Data were processed using Xcalibur QualBrowser (Thermo Fisher Scientific) software (version 2.0.7).

HESI analysis was conducted in the same manner as the APCI analysis above with the modification that that ionization was performed using a heated electrospray source (positive-ion mode) with a vaporizer temperature of 300 °C and scanning from *m*/*z* 100–500. The linear gradient (flow rate 0.3 mL min^–1^) was also modified for steroids as follows (expressed as %B, v/v): 0 min, 50%; 0.5 min, 50%; 3 min, 100%; 4.5 min, 100%; 4.6 min, 50%; 6 min, 50%. For oxysterols, the gradient was the same method as with the APCI analysis above, but the flow rate was increased to 0.3 mL min^–1^. For all sterols, the sample was injected with 100% B at a flow rate of 0.6 mL min^–1^ for 10 min. For HESI analyses with NH_4_F, all conditions were kept as described herein with the modification that the mobile phase composition was changed to 0.3 mM NH_4_F in (A) H_2_O and (B) CH_3_OH. For the LC-HESI-MS/MS analyses, the same method was used with the modifications that certain ions identified in the LC-MS run were targeted and fragmented using a collision energy of 35 V.

## Results

### Initial MS Results

In the course of the synthesis of several hydroxy sterols for other projects, we used APCI MS to verify the results of several reductions and oxidations. ESI proved to be rather insensitive to routinely obtain spectra of sterols, and we employed the general use of APCI^+^ MS for the analysis of sterols, other steroids, and retinoids.^10–12,23–30^ Several of the spectra of our synthetic derivatives were problematic in deciding whether reactions had been successful (e.g., NaBH_4_ and LiAlH_4_ reductions) due to presence of peaks 2 amu lower than expected (i.e., a MH^+^-2 ion of the product will present with the same *m*/*z* as the MH^+^ ion of the starting material, Scheme 1). However, analysis of the MH^+^–18 ion (loss of H_2_O) in these samples confirmed a 2 amu *m*/*z* increase (alluding to a successful NaBH_4_ reduction), and a complete reaction was further verified by analysis with TLC and NMR. Due to the dominance and unknown nature of these 2 amu losses, we decided that an investigation into prominence of this effect in the steroid family of molecules was in order.

### MS Fragmentation

We examined a series of 44 sterols and other steroids (Scheme 2, mainly hydroxy) on hand from synthetic work and commercial sources using APCI-MS and HESI-MS, which have been reported to be more useful than standard ESI MS for steroids.^31,32^ Lists of ions and fragments of the tested steroids and sterols are presented in Table 1, and mass spectra are presented in the Supporting Information (Supporting Figures S1–S44). As expected, the MS spectra of most of these compounds were dominated by MH^+^-18*n* ions (i.e., loss of *n* × H_2_O). However, prominent MH^+^-2 and MH^+^-4 ions were often observed (Figure 1).

**Table 1:** APCI and HESI Analyses of the Steroids^a^

		Ion assignment		APCI		HESI	
	Steroid, calc. MH^+^	Theoretical *m*/*z*	Assignment	Prominent ions, *m*/*z*	Intensity	Prominent ions, *m*/*z*	Intensity
**Estrogens**							
**1**	17β-Estradiol, *m*/*z* 273.1849	272.1776	M^+^	ND		**272.1766**	1.5 × 10^4^
		271.1693	MH^+^-2	271.1695	3.2 × 10^4^	**ND**	
		255.1743	MH^+^-H_2_O	255.1745	4.1 × 10^4^	**255.1735**	**9.2 × 10^4^**
**2**	Estrone, *m*/*z* 271.1693	271.1693	MH^+^-H_2_O	271.1699	3.2 × 10^4^	**271.1684**	**1.7 × 10^5^**
**3**	Estriol, *m*/*z* 289.1798	288.1725	M^+^	ND		**288.1713**	**1.9 × 10^4^**
		287.1642	MH^+^-2	287.1653	2.5 × 10^5^	**ND**	
		285.1485	MH^+^-4	285.1487	2.0 × 10^4^	**ND**	
		271.1693	MH^+^-H_2_O	271.1693	1.3 × 10^4^	**ND**	
		269.1536	MH^+^-H_2_O-2	269.1537	3.6 × 10^4^	**ND**	
**Androgens**							
**4**	Androstenedione, *m*/*z* 287.2006	287.2006	MH^+^	287.2010	1.10 × 10^7^	287.2009	2.4 × 10^7^
		285.1849	MH^+^-2	285.1855	1.9 × 10^6^	ND	
		283.1693	MH^+^-4	283.1697	2.8 × 10^5^	ND	
**5**	Testosterone, *m*/*z* 289.2162	289.2162	MH^+^	289.2170	1.6 × 10^7^	289.2160	2.3 × 10^7^
		287.2006	MH^+^-2	287.2016	2.3 × 10^6^	ND	
		285.1849	MH^+^-4	285.1859	7.8 × 10^5^	ND	
		271.2056	MH^+^-H_2_O	271.2064	1.4 × 10^6^	ND	
**6**	6β-OH testosterone, *m*/*z* 305.2111	305.2111	MH^+^	305.2113	3.2 × 10^5^	305.2119	2.5 × 10^6^
		303.1955	MH^+^-2	303.1956	2.3 × 10^5^	ND	
		301.1798	MH^+^-4	301.1799	1.0 × 10^5^	ND	
		287.2006	MH^+^-H_2_O	287.2007	2.9 × 10^5^	ND	
		269.1900	MH^+^-2H_2_O	269.1900	4.2 × 10^4^	ND	
**7**	5α-Dihydrotestosterone, *m*/*z* 291.2319	291.2319	MH^+^	291.2326	2.0 × 10^5^	291.2325	6.8 × 10^5^
		273.2213	MH^+^-H_2_O	273.2219	7.2 × 10^4^	273.2217	5.3 × 10^4^
		255.2107	MH^+^-2H_2_O	255.2110	2.1 × 10^4^	ND	
**8**	19-Nortestosterone, *m*/*z* 275.2006	275.2006	MH^+^	275.2011	1.2 × 10^7^	275.2010	1.6 × 10^7^
		273.1849	MH^+^-2	273.1856	7.2 × 10^5^	ND	
		257.1900	MH^+^-H_2_O	257.1905	8.6 × 10^5^	257.1902	1.7 × 10^5^
**9**	19-Norandrostenedione, *m*/*z* 273.1849	273.1849	MH^+^	273.1852	1.1 × 10^7^	273.1849	2.2 × 10^7^
		271.1693	MH^+^-2	271.1697	6.0 × 10^5^	ND	
		255.1743	MH^+^-H_2_O	255.1746	2.3 × 10^5^	ND	
**10**	19-OH androstenedione, *m*/*z* 303.1955	303.1955	MH	303.1953	3.7 × 10^6^	303.1959	1.2 × 10^7^
**11**	11β-OH androstenedione, *m*/*z* 303.1955	303.1955	MH^+^	303.1966	1.7 × 10^7^	303.1959	1.3 × 10^7^
		301.1798	MH^+^-2	301.1811	1.9 × 10^6^	ND	
		285.1849	MH^+^-H_2_O	285.1859	2.0 × 10^6^	285.1850	1.6 × 10^5^
		267.1743	MH^+^-2H_2_O	267.1752	5.7 × 10^5^	267.1743	5.0 × 10^4^
**32**	Androstadieneone, *m*/*z* 271.2056	271.2056	MH^+^	271.2061	7.0 × 10^6^	271.2057	8.9 × 10^6^
		269.1900	MH^+^-2	269.1907	3.7 × 10^5^	ND	
**33**	Androstadienol, *m*/*z* 273.2213	273.2213	MH^+^	ND		ND	
		271.2056	MH^+^-2	271.2057	6.4 × 10^5^	271.2054	5.9 × 10^4^
		255.2107	MH^+^-H_2_O	255.2108	1.6 × 10^6^	255.2106	6.4 × 10^5^
**Sterols**							
**12**	Cholesterol, *m*/*z* 387.3621	385.3465	MH^+^-2	**385.3452**	**6.5 × 10^5^**	**ND**	
		383.3308	MH^+^-4	**383.3296**	**1.1 × 10^6^**	**ND**	
		369.3516	MH^+^-H_2_O	**369.3505**	**7.7 × 10^6^**	**369.3519**	**2.6 × 10^5^**
**13**	22(*R*)-OH cholesterol, *m*/*z* 403.3571	401.3414	MH^+^-2	401.3414	2.8 × 10^5^	ND	
		399.3258	MH^+^-4	399.3259	1.2 × 10^6^	ND	
		397.3101	MH^+^-6	397.3103	1.3 × 10^6^	ND	
		385.3465	MH^+^-H_2_O	385.3469	2.4 × 10^6^	385.3466	9.4 × 10^4^
		367.3359	MH^+^-2H_2_O	367.3362	2.6 × 10^6^	367.3361	1.2 × 10^5^
**14**	20(*R*)-,22(*R*)-(OH)_2_ cholesterol, *m*/*z* 419.3520	401.3414	MH^+^-H_2_O	401.3415	4.5 × 10^5^	401.3415	6.5 × 10^4^
		399.3258	MH^+^-H_2_O-2	399.3258	4.5 × 10^5^	ND	
		397.3101	MH^+^-H_2_O-4	397.3102	4.5 × 10^5^	ND	
		383.3308	MH^+^-2H_2_O	383.3310	3.3 × 10^6^	383.3313	3.4 × 10^5^
		365.3203	MH^+^-3H_2_O	365.3205	3.5 × 10^5^	365.3203	1.4 × 10^4^
**15**	17(*R*)-,20(*R*)-(OH)_2_ cholesterol, *m*/*z* 419.3520	401.3414	MH^+^-H_2_O	401.3416	6.0 × 10^5^	401.3413	1.2 × 10^5^
		399.3258	MH^+^-H_2_O-2	399.3258	2.8 × 10^5^	ND	
		397.3101	MH^+^-H_2_O-4	397.3102	2.2 × 10^5^	ND	
		383.3308	MH^+^-2H_2_O	383.3311	2.7 × 10^6^	383.3308	9.3 × 10^5^
		365.3203	MH^+^-3H_2_O	365.3206	1.2 × 10^6^	365.3203	6.1 × 10^4^
**40**	24,25-Dihydrolanosterol, *m*/*z* 429.4091	425.3778	MH^+^-4	**425.3772**	**2.3 × 10^4^**	**ND**	
		411.3985	MH^+^-H_2_O	**411.3975**	**3.6 × 10^6^**	**ND**	
**41**	14-CDO dihydrolanosterol, *m*/*z* 444.3946	444.3946	MH^+^	**444.3930**	**6.8 × 10^5^**	**444.3824**	**1.1 × 10^4^**
		426.3841	MH^+^-H_2_O	**426.3824**	**2.6 × 10^6^**	**426.3843**	**1.9 × 10^4^**
**42**	FF-MAS, *m*/*z* 413.3778	413.3778	MH^+^	**413.3768**	**2.0 × 10^6^**	**ND**	
		395.3672	MH^+^-H_2_O	**395.3665**	**1.6 × 10^5^**	**ND**	
**43**	β-Sitosterol, *m*/*z* 415.3934	413.3778	MH^+^-2	**413.3770**	**5.1 × 10^5^**	**ND**	
		411.3621	MH^+^-4	**411.3613**	**4.3 × 10^5^**	**ND**	
		397.3829	MH^+^-H_2_O	**397.3823**	**4.6 × 10^6^**	**ND**	
**44**	Ergosterol, *m*/*z* 397.3465	379.3359	MH^+^-H_2_O	**379.3356**	**2.0 × 10^4^**	**ND**	
**Pregnenolone derivatives (Δ^5^)**							
**16**	Pregnenolone, *m*/*z* 317.2475	317.2475	MH^+^	ND		317.2473	9.4 × 10^4^
		315.2319	MH^+^-2	315.2320	1.5 × 10^6^	ND	
		313.2162	MH^+^-4	313.2164	1.3 × 10^6^	ND	
		311.2006	MH^+^-6	311.2008	3.2 × 10^5^	ND	
		299.2369	MH^+^-H_2_O	299.2372	4.9 × 10^5^	299.2369	8.1 × 10^5^
**17**	3-OAc pregnenolone, *m*/*z* 359.2581	357.2424	MH^+^-2	357.2429	4.1 × 10^5^	357.2421	7.3 × 10^5^
		299.2369	MH^+^-OAc	299.2282	3.2 × 10^4^	299.2280	1.5 × 10^4^
		297.2213	MH^+^-OAc-2	297.2218	2.6 × 10^6^	297.2211	6.1 × 10^5^
**18**	Dehydroepiandosterone (DHEA), *m*/*z* 289.2162	289.2162	MH^+^	289.2165	7.0 × 10^4^	289.2166	3.5 × 10^4^
		287.2006	MH^+^-2	287.2009	1.1 × 10^6^	ND	
		285.1849	MH^+^-4	285.1853	6.4 × 10^5^	ND	
		271.2056	MH^+^-H_2_O	271.2060	6.7 × 10^5^	271.2061	3.9 × 10^5^
**31**	17α-OH pregnenolone, *m*/*z* 333.2424	331.2268	MH^+^-2	331.2274	3.7 × 10^4^	ND	
		329.2111	MH^+^-4	329.2115	1.2 × 10^4^	ND	
		315.2319	MH^+^-H_2_O	315.2292	3.5 × 10^4^	ND	
		313.2162	MH^+^-H_2_O-2	313.2169	1.3 × 10^5^	ND	
		311.2006	MH^+^-H_2_O-4	311.2011	1.0 × 10^5^	ND	
		297.2213	MH^+^-2H_2_O	297.2217	1.6 × 10^4^	ND	
**Progesterone derivatives (Δ^4^)**							
**22**	Algestone, *m*/*z* 347.2217	347.2217	MH^+^	347.2221	7.5 × 10^6^	347.2222	7.7 × 10^6^
		345.2060	MH^+^-2	345.2066	5.9 × 10^5^	345.2068	4.1 × 10^4^
**23**	11-OH progesterone, *m*/*z* 331.2268	331.2268	MH^+^	331.2270	3.0 × 10^7^	331.2267	1.9 × 10^7^
		329.2111	MH^+^-2	329.2117	3.3 × 10^6^	ND	
		313.2162	MH^+^-H_2_O	313.2166	4.0 × 10^6^	313.2162	2.8 × 10^5^
**24**	17α-OH progesterone, *m*/*z* 331.2268	331.2268	MH^+^	331.2275	1.3 × 10^7^	331.2272	3.4 × 10^7^
		329.2111	MH^+^-2	329.2122	5.2 × 10^5^	ND	
		313.2162	MH^+^-H_2_O	313.2171	3.4 × 10^6^	313.2162	1.5 × 10^5^
		311.2006	MH^+^-H_2_O-2	311.2015	1.3 × 10^6^	ND	
**39**	Progesterone, *m*/*z* 315.2319	315.2319	MH^+^	315.2328	2.0 × 10^7^	315.2327	4.0 × 10^7^
		313.2162	MH^+^-2	313.2176	3.2 × 10^6^	ND	
		311.2006	MH^+^-4	311.2020	6.9 × 10^5^	ND	
**Glucocorticoid**							
**25**	Hydrocortisone, *m*/*z* 363.2166	363.2166	MH^+^	363.2168	4.0 × 10^6^	363.2172	1.3 × 10^7^
		361.2010	MH^+^-2	361.2012	1.1 × 10^5^	361.2015	4.5 × 10^5^
			MH^+^-H_2_O	345.2062	1.4 × 10^5^	345.2061	4.2 × 10^4^
**Mineralocorticoid derivatives**							
**19**	21-OH progesterone, *m*/*z* 331.2268	331.2268	MH^+^	331.2269	1.3 × 10^7^	331.2273	2.0 × 10^7^
		329.2111	MH^+^-2	329.2113	2.0 × 10^6^	329.2119	6.5 × 10^5^
		327.1955	MH^+^-4	327.1957	1.3 × 10^6^	ND	
		325.1798	MH^+^-6	325.1801	7.8 × 10^5^	ND	
		313.2162	MH^+^-H_2_O	313.2164	1.4 × 10^6^	313.2166	1.4 × 10^4^
**20**	21-OAc progesterone, *m*/*z* 373.2373	373.2373	MH^+^	373.2374	1.3 × 10^7^	373.2378	3.9 × 10^7^
		313.2162	MH^+^-OAc	313.2165	4.1 × 10^6^	313.2164	8.6 × 10^4^
		311.2006	MH^+^-OAc-2	311.2009	9.1 × 10^5^	ND	
**21**	Aldosterone, *m*/*z* 361.2010	343.1904	MH^+^-H_2_O	343.1911	7.7 × 10^6^	343.1906	9.1 × 10^6^
		341.1747	MH^+^-H_2_O-2	341.1755	2.4 × 10^5^	ND	
		325.1798	MH^+^-2H_2_O	325.1805	2.6 × 10^5^	325.1798	1.0 × 10^5^
**26**	3β-OH-preg-5-ene-20,21-diol, *m*/*z* 335.2581	333.2424	MH^+^-2	ND		333.2432	3.5 × 10^5^
		317.2475	MH^+^-H_2_O	317.2476	1.5 × 10^4^	317.2480	2.3 × 10^4^
		299.2369	MH^+^-2H_2_O	299.2373	7.5 × 10^4^	299.2373	1.0 × 10^5^
		281.2264	MH^+^-3H_2_O	281.2267	1.2 × 10^5^	281.2269	1.9 × 10^5^
**27**	[3,20-*d*_2_] 3β-OH-preg-5-ene-20,21-diol, *m*/*z* 337.2706	334.2487	MH^+^-3	ND		334.2488	4.4 × 10^5^
		319.2601	MH^+^-H_2_O	319.2600	1.4 × 10^4^	319.2600	2.8 × 10^4^
		301.2495	MH^+^-2H_2_O	301.2495	1.0 × 10^5^	301.2497	2.0 × 10^5^
		283.2389	MH^+^-3H_2_O	283.2390	1.9 × 10^5^	283.2390	2.8 × 10^5^
**38**	Corticosterone, *m*/*z* 347.2217	347.2217	MH^+^	347.2222	2.0 × 10^6^	347.2223	6.0 × 10^6^
		345.2060	MH^+^-2	345.2067	2.1 × 10^5^	345.2065	2.2 × 10^4^
		343.1904	MH^+^-4	343.1908	7.8 × 10^4^	ND	
		329.2111	MH^+^-H_2_O	329.2116	1.3 × 10^5^	329.2114	4.2 × 10^4^
		311.2006	MH^+^-2H_2_O	311.2010	8.4 × 10^4^	311.2070	1.4 × 10^4^
**Miscellaneous steroids**							
**28**	3-Keto-4-etiocholenic acid, *m*/*z* 317.2111	317.2111	MH^+^	317.2114	7.8 × 10^6^	317.2119	2.2 × 10^7^
		315.1955	MH^+^-2	315.1960	4.1 × 10^5^	ND	
**29**	Etienic acid, *m*/*z* 319.2268	317.2111	MH^+^-2	317.2109	2.8 × 10^4^	ND	
		315.1955	MH^+^-4	315.1952	2.8 × 10^4^	ND	
		301.2162	MH^+^-H_2_O	301.2162	3.3 × 10^5^	301.2162	2.2 × 10^5^
		299.2006	MH^+^-H_2_O-2	299.2005	1.4 × 10^4^	ND	
**30**	24-Bisnor-5-cholenic acid-3β-ol, *m*/*z* 347.2581	345.2424	MH^+^-2	345.2429	1.8 × 10^4^	ND	
		329.2475	MH^+^-H_2_O	329.2480	8.5 × 10^5^	329.2477	4.8 × 10^5^
		327.2319	MH^+^-H_2_O-2	327.2322	2.7 × 10^4^	ND	
**34**	Androst-5-ene-3β,17-diol, *m*/*z* 291.2319	289.2162	MH^+^-2	289.2162	5.6 × 10^5^	ND	
		287.2006	MH^+^-4	287.2006	8.3 × 10^5^	ND	
		285.1849	MH^+^-6	285.1849	5.9 × 10^5^	ND	
		273.2213	MH^+^-H_2_O	273.2213	6.8 × 10^5^	273.2211	4.6 × 10^5^
		271.2056	MH^+^-H_2_O-2	271.2055	5.7 × 10^4^	ND	
		255.2107	MH^+^-2H_2_O	255.2108	3.1 × 10^5^	255.2105	1.1 × 10^5^
**35**	17β-OH methyl-Δ^5^-androstene-3β-ol, *m*/*z* 305.2475	303.2319	MH^+^-2	303.2318	2.3 × 10^5^	303.2320	2.8 × 10^5^
		301.2162	MH^+^-4	301.2162	1.8 × 10^5^	ND	
		287.2369	MH^+^-H_2_O	287.2370	1.8 × 10^5^	287.2372	5.9 × 10^5^
		269.2264	MH^+^-2H_2_O	269.2264	8.1 × 10^5^	269.2265	5.4 × 10^5^
**36**	*d*_2_-17β-OH methyl-Δ^5^-androstene-3β-ol, *m*/*z* 307.2601	305.2444	MH^+^-2	305.2450	1.6 × 10^5^	ND	
		302.2225	MH^+^-5	302.2231	7.1 × 10^4^	ND	
		289.2495	MH^+^-H_2_O	289.2501	3.1 × 10^5^	289.2497	3.0 × 10^5^
		271.2389	MH^+^-2H_2_O	271.2395	2.5 × 10^5^	271.2390	5.2 × 10^4^
**37**	17β-Formyl-Δ^5^-androstene-3β-ol, *m*/*z* 303.2319	303.2319	MH^+^	303.2326	2.0 × 10^5^	ND	
		301.2162	MH^+^-2	301.2170	2.0 × 10^6^	301.2166	1.9 × 10^6^
		299.2006	MH^+^-5	299.2014	5.4 × 10^5^	ND	
		285.2213	MH^+^-H_2_O	285.2220	7.4 × 10^5^	285.2216	1.2 × 10^6^

a Values recorded in plain text were determined at 100 μM steroid concentration, while values in bold text were determined at 500 μM (injection volume 10 μL, i.e., 1 or 5 nmol). ND: not detected (response <1 × 10^4^).

**Figure 1:**
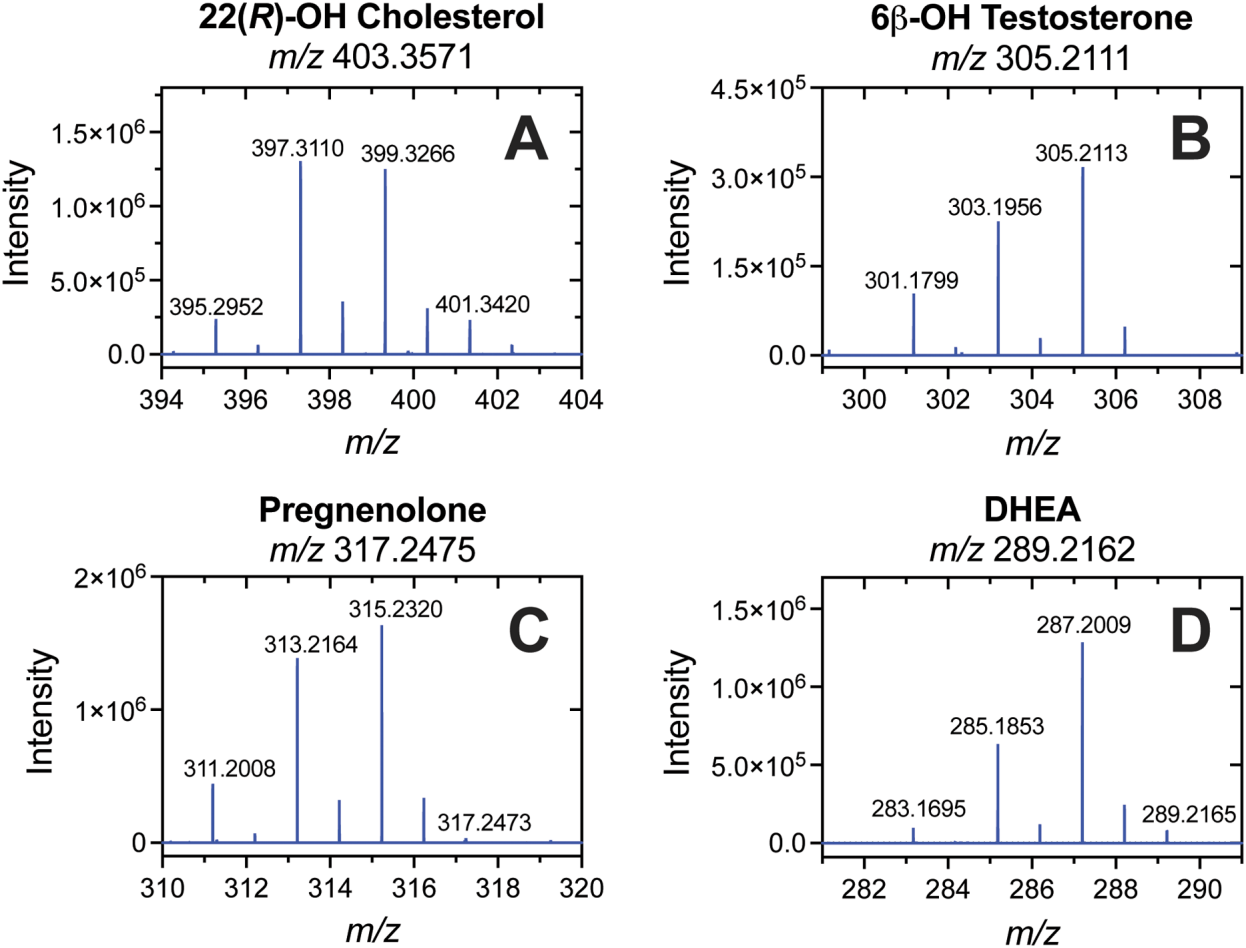
Mass spectra of four molecules showing strong 2*n* amu losses (2-electron oxidations, *n*= 1, 2, or 3). (A) 22­(*R*)-OH cholesterol (**13**), (B) 6β-OH testosterone (**6**), (C) pregnenolone (**16**), and (D) dehydroepiandrosterone (DHEA, **18**). The exact *m*/*z* value of the MH^+^ precursor ion is indicated above each panel. The major ions detected are printed on each panel (note: the MH^+^ ion is not always detected).

In the cases of 22­(*R*)-OH cholesterol (**13**, Figure 1A), pregnenolone (**16**, Figure 1C), and dehydroepiandrosterone (DHEA, **18**, Figure 1D) the MH^+^ ion was either absent or present in very low abundance (near baseline level). With 6β-OH testosterone (**6**, Figure 1B) the MH^+^ ion was the base peak, though a strong prevalence of MH^+^-2 and MH^+^-4 ions was observed. Interestingly, in the case of the six Δ^5^ steroids and sterols (i.e., C-3 hydroxy; pregnenolone and DHEA), a 2*n*-electron oxidation was the base peak rather than the generally dominant MH^+^-H_2_O (−18 amu) ion (Supporting Information, compounds **16**, **17**, **18**, **31**, **34**, and **37**). (This includes the possibility of a 2*n*-electron oxidation occurring in combination with the loss of a water or acetoxy group, i.e., [MH^+^–H_2_O– 2*n*] ions). In most (5/6) cases, the base peak was a single 2*n*-electron oxidation (i.e., *n* = 1), with the exception of androst-5-ene-3β-ol (**34**, *n* = 2). A 2*n*-electron oxidation was also the base peak for the estrogen estriol (**3**, *n* = 1). In cases where multiple hydroxyl groups are present in the molecule, the base peak may be the loss of *n* × H_2_O (e.g., a value of *n* = 2 was observed as the base peak of 22­(*R*)-hydroxycholesterol (**13**), Table 1, Supporting Information). Of the 44 steroids and sterols analyzed, 36 showed some form of a 2*n* amu loss in APCI MS analysis (17/22 Δ^5^ steroids, 17/19 Δ^4^ steroids, and 2/3 estrogens) (Table 1).

In the case of a deuterated steroid (**35** and **36**), the prevalence of a MH^+^-5 ion (loss of 2H from one site and H + D from another, Figure 2A) provided further evidence that the observed MH^+^-2*n* ions were products of carbinol oxidation, i.e., the loss of deuterium was only possible at one site.

**Figure 2:**
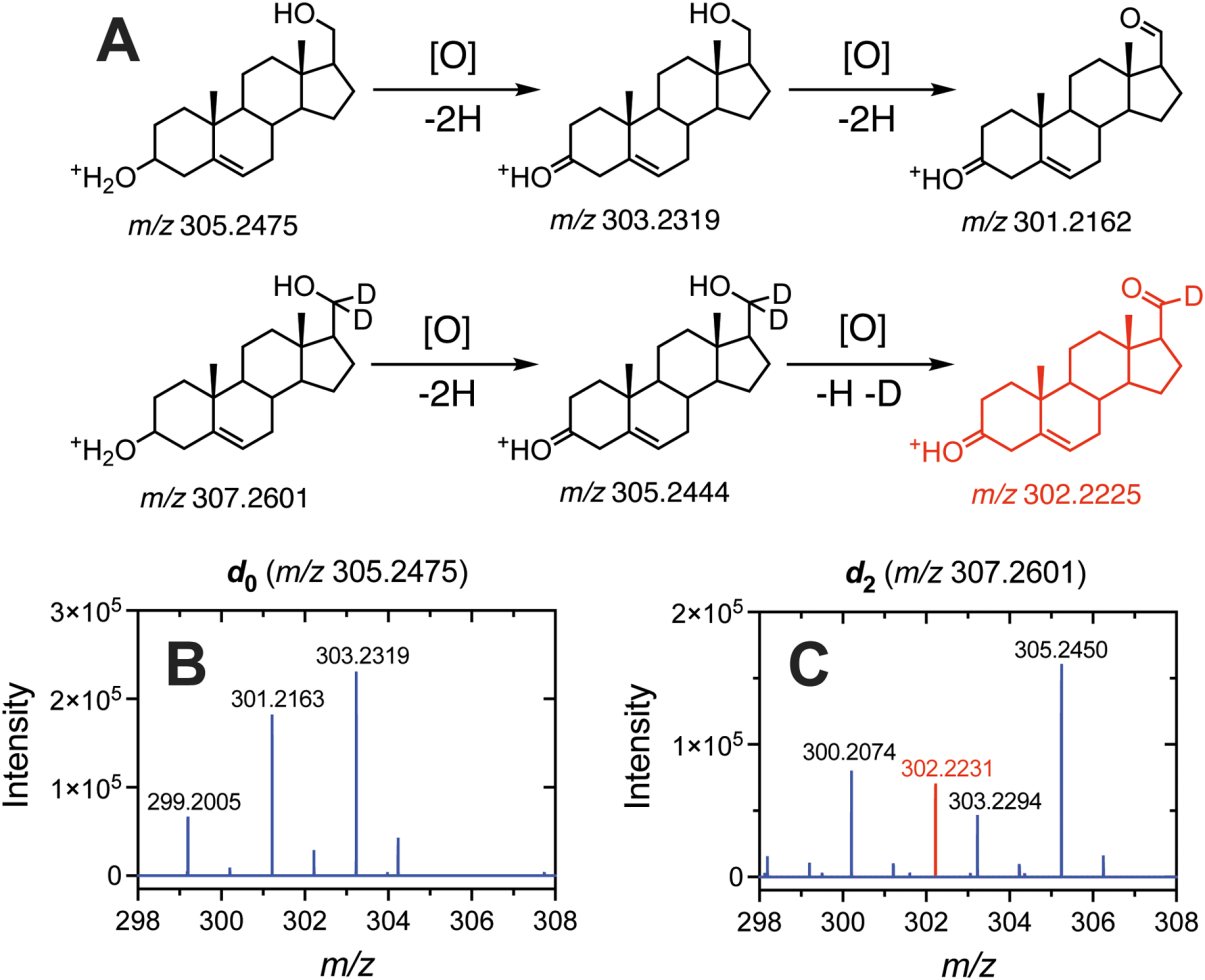
Comparison of the APCI mass spectra *d*_0_-(**35**) and *d*_2_-17β-OH methyl-Δ^5^-androstene-3β-ol (**36**). (A) Scheme illustrating the products of the oxidation of the 3β-OH group followed by the 17β-OH group of *d*_0_- (upper) and *d*_2_-17β-OH methyl-Δ^5^-androstene-3β-ol (lower). The exact *m*/*z* values are displayed under each molecule. Mass spectra of 100 μM standards of (B) *d*_0_-17β-OH methyl-Δ^5^-androstene-3β-ol (**35**) and (C) *d*_2_-17β-OH methyl-Δ^5^-androstene-3β-ol (**36**) are shown (1 nmol injected), with the most prominent ions labeled. Ions colored red result from the loss of deuterium.

In the case of *d*_0_-17β-OH methyl-Δ^5^-androstene-3β-ol (**35**), the dominant ions were MH^+^-2, MH^+^-4, and MH^+^-6 (Figure 2B) as we observed with other steroids (Figure 1, Supporting Information). When carbon C-17 was deuterated (*d*_2_-17β-OH methyl-Δ^5^-androstene-3β-ol, **36**, Figure 2A), the distribution shifted to MH^+^-2, MH^+^-5, and MH^+^-7 (Figure 2C). We did not observe any 3 or 5 amu losses in nondeuterated steroids.

In HESI-MS analysis of the same steroid library, 2*n* electron oxidations were less prevalent (Figure 3, Supporting Information).

**Figure 3:**
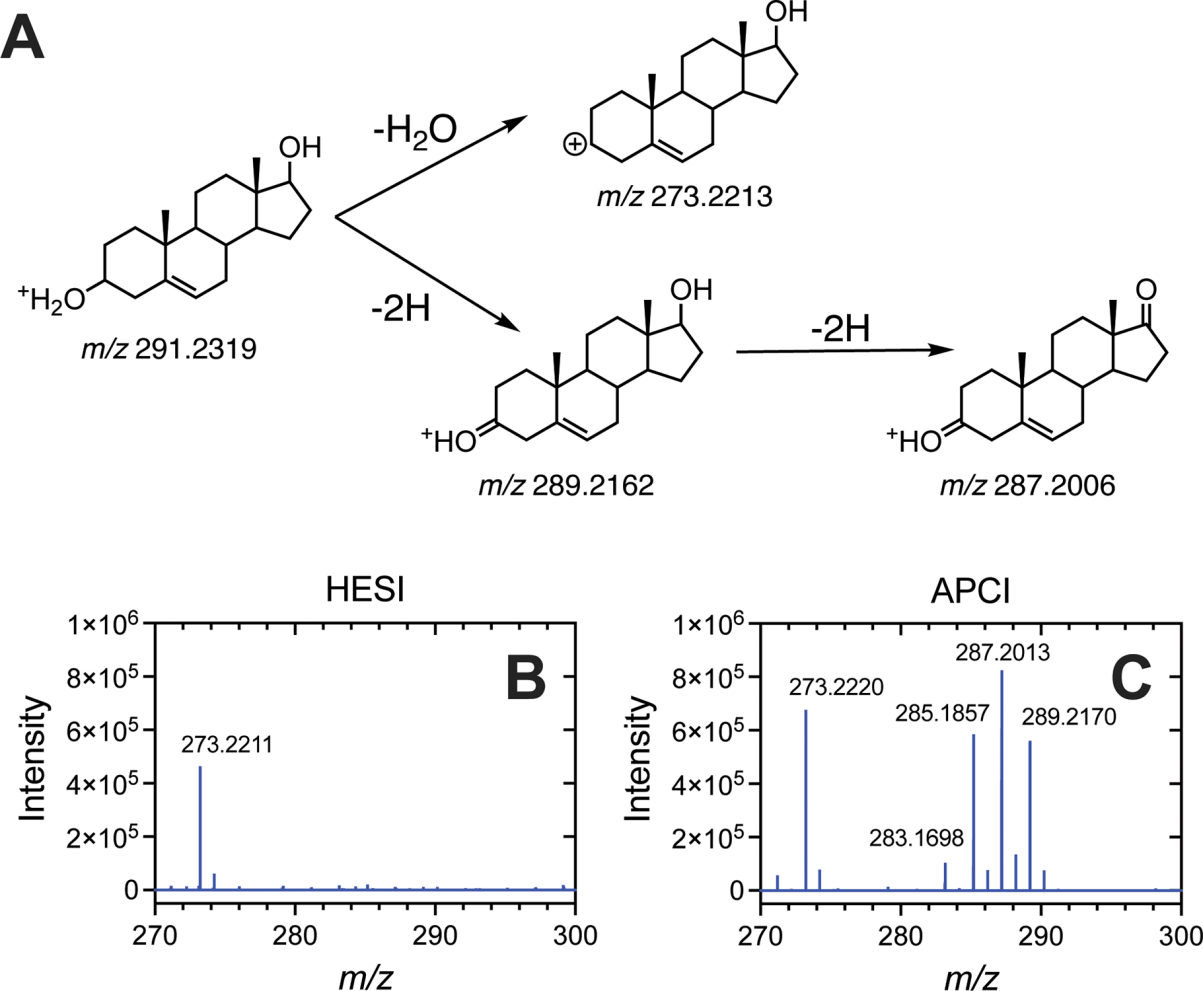
Comparison of HESI and APCI MS spectra of androst-5-ene-3β,17-diol (**34**). (A) Scheme illustrating the products of the loss of the 3β-OH group (−18 amu, upper) or two sequential oxidations of the 3β-OH and 17β-OH groups (−2 amu, lower) of androst-5-ene-3β,17-diol (**34**). The exact *m*/*z* values are displayed under each molecule. (B) HESI and (C) APCI MS spectra of a 100 μM standard of androst-5-ene-3β,17-diol (**34**), with the most prominent ions labeled.

However, the losses were detected (generally weakly) in 9 of the 44 analyzed steroids and sterols (Supporting Information, compounds **17**, **19**, **22**, **25**, **26**, **27**, **35**, **37**, and **38**) and constituted the base peak in 3 of those (**26**, **27**, and **37**). In an example case of a typical APCI vs HESI MS comparison, androst-5-ene-3β,17-diol (**34**) HESI-MS analysis yielded two dominant ions, MH^+^-18 and MH^+^-36 (both losses of *n* × H_2_O), with the former ion being the base peak (Figure 3B). Conversely, when the same sample was analyzed via APCI-MS, a series of MH^+^-2*n* ions was generated in addition to the MH^+^-18 ion (which was similar in intensity to the HESI-MS analyzed sample), with the MH^+^-4 ion being the base peak (Figure 3A). Neither ionization method revealed the MH^+^ ion. APCI MS frequently generated more ions, generally as −2 amu losses, than the same steroid subjected to HESI MS (Supporting Information).

### Alternative Mechanism for Ion Generation

A recent report of LC-ESI MS studies of the sesquiterpene artemisinic acid identified MH^+^-2 ions in chromatograms but attributed the origin to a mechanism involving the loss of allylic hydride (i.e., MH^+^-2H = M^+^-H).^33^ To determine whether such a mechanism might also generate the MH^+^-2 ions we report primarily in APCI analyses of Δ^5^ steroids, we reasoned that reduction of the Δ^5^ bond and thus elimination of the allylic site should reveal the contribution of allylic hydride loss to artifactual ion generation. We selected dehydroepiandrosterone (**18**), a Δ^5^ steroid with dominant MH^+^-2*n* ions (Table 1, Figure 1), for this experiment and subjected the molecule to hydrogenation using palladium-on-carbon as a catalyst to yield *allo*-DHEA.^34^ LC-APCI-MS analysis of the crude sample revealed the starting material (DHEA) and product (*allo*-DHEA) (Supporting Figure S45). As previously reported, the MH^+^-2*n* ions of DHEA were dominant with only a weak MH^+^ ion (*m*/*z* 289) detected (Figure 1). When the Δ^5^ bond was reduced (to yield *allo*-DHEA), the ion distribution shifted in the direction of MH^+^-(*n* × H_2_O) and MH^+^ ions, where the MH^+^ ion (sparsely detected for DHEA) appeared at ∼20% relative intensity and the MH^+^-(*n* × H_2_O) ions (*n* = 1, 2) were observed at ∼95% and ∼90% relative intensity, respectively (roughly ∼50% and ∼20%, respectively, for DHEA). However, in *allo*-DHEA the base peak was still a MH^+^-2*n* ion, as had been shown for DHEA. Further efforts were made to assess the requirement for protons from the HPLC mobile phase in generating MH^+^-2*n* ions (carbinol oxidation vs hydride loss) using deuterated ionization salts (ND_4_OAc, DCO_2_D), although the data were inconclusive.

Further efforts to characterize the MH^+^-2 ions were conducted by using targeted LC-MS/MS fragmentation analysis. Compound **37** was selected as a molecule that demonstrated prominent MH^+^-2 ions in both APCI^+^ and HESI^+^ MS (Supporting Figure S37) and was subjected to both APCI^+^ and HESI^+^ MS/MS analysis targeting the MH^+^ ion (*m*/*z* 303), the MH^+^-2 ion (*m*/*z* 301), and the MH^+^-H_2_O ion (*m*/*z* 285). As previously observed, the MH^+^-2 ion was dominant, and subsequent fragmentation of that ion revealed identical product ions with both APCI^+^ and HESI^+^, which were MH^+^-(*n* × H_2_O)-2 ions (Supporting Figure S46). APCI^+^ and HESI^+^ MS/MS analysis of the MH^+^-2 ion yielded nearly identical *m*/*z* spectra, suggesting they correspond to the same structure in both ionization methods. The MH^+^-H_2_O ion (*m*/*z* 285) observed in the untargeted analysis (Supporting Figure S37) was not detected as a fragment ion in the targeted run, and the base peak was the MH^+^-H_2_O-2 ion (*m*/*z* 283) that was detected near the baseline level in the untargeted approach (Supporting Information, Figure S37). Oxidation of the C-3 carbinol of **37** (detected as a MH^+^-2 ion) eliminates the only hydroxyl moiety in the structure (Scheme 2) that might protonate and leave (as H_2_O), yet a MH^+^-H_2_O-2 ion was detected as the base peak in the targeted run. To assess the necessity of free hydroxyl moieties for the generation of MH^+^-H_2_O ions, APCI-MS/MS fragmentation of progesterone (**39**, a Δ^4^ steroid with C-3 and C-20 ketones) was conducted. MS/MS fragmentation of the precursor ion (*m*/*z* 315, the base peak in the untargeted run) yielded product ions of MH^+^-(*n* × H_2_O), and a base peak was observed where *n* = 1 (*m*/*z* 297, Supporting Figure S46), an ion that was not detected in the untargeted run (Supporting Figure S39). Formation of the MH^+^-H_2_O ion was thus not observed to be dependent on the presence of a carbinol in the molecular structure.

### MS Sensitivity

In addition to a difference in ions generated by each ionization method (Table 1, Figure 3, Supporting Information), we also predictably observed differences in overall detection sensitivity. We compared the sensitivity of APCI and HESI with all 44 steroids in Table 2 (mass spectra are available in the Supporting Information) using a conventional mobile phase of 0.1% HCO_2_H in H_2_O/CH_3_CN (see Experimental Section).

**Table 2:** Comparison of APCI and ESI^a^

		APCI		HESI		
	Steroid, calc. MH^+^	Base peak, *m*/*z*	Intensity	Base peak, *m*/*z*	Intensity	Ratio HESI/APCI
**Estrogens**						
**1**	17β-Estradiol, *m*/*z* 273.1849	255.1745	4.1 × 10^4^	**255.1735**	**9.2 × 10^4^**	2.2
**2**	Estrone, *m*/*z* 271.1693	271.1699	3.2 × 10^4^	**271.1684**	**1.7 × 10^5^**	5.3
**3**	Estriol, *m*/*z* 289.1798	287.1653	2.5 × 10^5^	**288.1713**	**1.9 × 10^4^**	0.08
**Androgens**						
**4**	Androstenedione, *m*/*z* 287.2006	287.2010	1.1 × 10^7^	287.2009	2.4 × 10^7^	2.2
**5**	Testosterone, *m*/*z* 289.2162	289.2170	1.6 × 10^7^	289.2160	2.3 × 10^7^	1.4
**6**	6β-OH testosterone, *m*/*z* 305.2111	305.2113	3.2 × 10^5^	305.2119	2.5 × 10^6^	7.8
**7**	5α-Dihydrotestosterone, *m*/*z* 291.2319	291.2326	2.0 × 10^5^	291.2325	6.8 × 10^5^	3.4
**8**	19-Nortestosterone, *m*/*z* 275.2006	275.2011	1.2 × 10^7^	275.2010	1.6 × 10^7^	1.3
**9**	19-Norandrostenedione, *m*/*z* 273.1849	273.1852	1.1 × 10^7^	273.1849	2.2 × 10^7^	2.0
**10**	19-OH androstenedione, *m*/*z* 303.1955	303.1953	3.7 × 10^6^	303.1959	1.2 × 10^7^	3.2
**11**	11β-OH androstenedione, *m*/*z* 303.1955	303.1966	1.7 × 10^7^	303.1959	1.3 × 10^7^	0.8
**32**	Androstadieneone, *m*/*z* 271.2056	271.2061	7.0 × 10^6^	271.2057	8.9 × 10^6^	1.3
**33**	Androstadienol, *m*/*z* 273.2213	255.2108	1.6 × 10^6^	255.2106	6.4 × 10^5^	0.4
**Sterols**						
**12**	Cholesterol, *m*/*z* 387.3621	**369.3505**	**7.7 × 10^6^**	**369.3519**	**2.6 × 10^5^**	0.03
**13**	22(*R*)-OH cholesterol, *m*/*z* 403.3571	367.3362	2.6 × 10^6^	367.3361	1.2 × 10^5^	0.05
**14**	20(*R*)-,22(*R*)-(OH)_2_ cholesterol, *m*/*z* 419.3520	383.3310	3.3 × 10^6^	383.3313	3.4 × 10^5^	0.1
**15**	17(*R*)-,20(*R*)-(OH)_2_ cholesterol, *m*/*z* 419.3520	383.3311	2.7 × 10^6^	383.3308	9.3 × 10^5^	0.3
**40**	24,25-Dihydrolanosterol, *m*/*z* 429.4091	**411.3975**	**3.6 × 10^6^**	**ND**		N/A
**41**	14-CDO dihydrolanosterol, *m*/*z* 444.3946	**426.3824**	**2.6 × 10^6^**	**426.3843**	**1.9 × 10^4^**	0.007
**42**	FF-MAS, *m*/*z* 413.3778	**413.3768**	**2.0 × 10^6^**	**ND**		N/A
**43**	β-Sitosterol, *m*/*z* 415.3934	**397.3823**	**4.6 × 10^6^**	**ND**		N/A
**44**	Ergosterol, *m*/*z* 397.3465	**379.3356**	**2.0 × 10^4^**	**ND**		N/A
**Pregnenolone derivatives**						
**16**	Pregnenolone, *m*/*z* 317.2475	315.2320	1.5 × 10^6^	299.2369	8.1 × 10^5^	0.5
**17**	3-OAc pregnenolone, *m*/*z* 359.2581	297.2218	2.6 × 10^6^	357.2421	7.3 × 10^5^	0.3
**18**	Dehydroepiandosterone, *m*/*z* 289.2162	287.2009	1.1 × 10^6^	271.2061	3.9 × 10^5^	0.4
**31**	17α-OH pregnenolone, *m*/*z* 331.2268	313.2169	1.3 × 10^5^	ND		N/A
**Progesterone derivatives**						
**22**	Algestone, *m*/*z* 347.2217	347.2221	7.5 × 10^6^	347.2222	7.7 × 10^6^	1.0
**23**	11-OH progesterone, *m*/*z* 331.2268	331.2270	3.0 × 10^7^	331.2267	1.9 × 10^7^	0.6
**24**	17α-OH progesterone, *m*/*z* 331.2268	331.2275	1.3 × 10^7^	331.2272	3.4 × 10^7^	2.6
**39**	Progesterone, *m*/*z* 315.2319	315.2328	2.0 × 10^7^	315.2327	4.0 × 10^7^	2.0
**Glucocorticoid**						
**25**	Hydrocortisone, *m*/*z* 363.2166	363.2168	4.0 × 10^6^	363.2172	1.3 × 10^7^	3.3
**Mineralocorticoid derivatives**						
**19**	21-OH progesterone, *m*/*z* 331.2268	331.2269	1.3 × 10^7^	331.2273	2.0 × 10^7^	1.5
**20**	21-OAc progesterone, *m*/*z* 373.2373	373.2374	1.3 × 10^7^	373.2378	3.9 × 10^7^	3.0
**21**	Aldosterone, *m*/*z* 361.2010	343.1911	7.7 × 10^6^	343.1906	9.1 × 10^6^	1.2
**26**	3β-OH-preg-5-ene-20,21-diol, *m*/*z* 335.2581	281.2267	1.2 × 10^5^	333.2432	3.5 × 10^5^	2.9
**27**	[3,20-*d*_2_] 3β-OH-preg-5-ene-20,21-diol, *m*/*z* 337.2706	283.2390	1.9 × 10^5^	334.2488	4.4 × 10^5^	2.3
**38**	Corticosterone, *m*/*z* 347.2217	347.2222	2.0 × 10^6^	347.2223	6.0 × 10^6^	3.0
**Miscellaneous steroids**						
**28**	3-Keto-4-etiocholenic acid, *m*/*z* 317.2111	317.2114	7.8 × 10^6^	317.2119	2.2 × 10^7^	2.8
**29**	Etienic acid, *m*/*z* 319.2268	301.2162	3.3 × 10^5^	301.2162	2.2 × 10^5^	0.7
**30**	24-Bisnor-5-cholenic acid-3β-ol, *m*/*z* 347.2581	329.2480	8.5 × 10^5^	329.2477	4.8 × 10^5^	0.6
**34**	Androst-5-ene-3β,17-diol, *m*/*z* 291.2319	287.2006	8.3 × 10^5^	273.2211	4.6 × 10^5^	0.6
**35**	17β-OH methyl-Δ^5^-androstene-3β-ol, *m*/*z* 305.2475	269.2264	8.1 × 10^5^	287.2372	5.9 × 10^5^	0.7
**36**	*d*_2_-17β-OH methyl-Δ^5^-androstene-3β-ol, *m*/*z* 307.2601	289.2501	3.1 × 10^5^	289.2497	3.0 × 10^5^	1.0
**37**	17β-Formyl-Δ^5^-androstene-3β-ol, *m*/*z* 303.2319	301.2170	2.0 × 10^6^	301.2166	1.9 × 10^6^	1.0

a Values recorded in plain text were determined at 100 μM steroid concentration, while values in bold text were determined at 500 μM (i.e., 10 μL injected = 1 or 5 nmol). Differences in steroid concentration were accounted for, where appropriate, in the calculation of the intensity ratio. ND: not detected (response <1 × 10^4^). N/A: cannot compute signal ratio, as one ionization method did not yield a detectable signal.

In general, the difference of the base peak intensity between APCI and HESI for the 44 tested compounds uncommonly exceeded 3-fold. The major exceptions to this were sterols (**12**–**15**) and pregnenolone derivatives (Δ^5^ steroids, **16**–**18**, **31**), which generally demonstrated a pronounced advantage of APCI-MS over HESI-MS (Table 2). For the tested sterols, the effect was a minimum of a 3.3-fold increase in sensitivity with APCI MS, but was 30-fold for cholesterol (**12**) and 137-fold for 14-CDO dihydrolanosterol (**41**), and several sterols were only detectable with APCI-MS (**40**, **42**–**44**) (Table 2). For pregnenolone derivatives, the advantage of APCI was >2-fold (17-OH pregnenolone (**31**) was not detected using a conventional HESI method). For glucocorticoids (**25**) and mineralocorticoids or trihydroxypregnanes (**19**–**21**, **25**–**27**, **38**), HESI was generally preferable, giving base peak intensities 1.0–3.3-fold higher than those of APCI-MS. For derivatives of androstenedione (**4**–**11**, **32**, **33**) and progesterone (**22**–**24**, **39**) (all almost exclusively Δ^4^ steroids), HESI was again generally preferred, with base peak intensities 0.6–3.4-fold those of APCI, with the main exceptions being a 7.8-fold increase in signal observed with 6β-OH testosterone (**6**) and a 2.5-fold decrease in signal with androstadienol (**33**, the lone Δ^5^ steroid). For the remaining “miscellaneous” steroids (**28**–**30**, **34**–**37**), the majority of which are Δ^5^ steroids, there was minimal difference between the methods with base peak intensities being 1.0–1.7-fold those of APCI, the exception being 3-keto-4-etiocholenic acid (**28**), the lone Δ^4^ steroid, with a 2.8-fold advantage of HESI.

### Effect of NH_4_F

The analysis of steroids via HESI-MS has been reported to be enhanced by the use of NH_4_F as a mobile phase additive in both positive and negative ion modes.^35–37^ Accordingly, we also analyzed our library of 44 steroids and sterols with HESI MS and the addition of 0.3 mM NH_4_F to the mobile phase. For most of the tested steroids, the intensity of the base peak increased in the presence of NH_4_F, but rarely >4-fold (Table 3). Increases of 1.2–4.0-fold were observed for Δ^4^ steroids (androgens (**4**–**11**, **32**, **33**), progesterone derivatives (**22**–**24**, **39**), and a “miscellaneous” steroid (**28**)), 1.7–4.8-fold for glucocorticoids (**25**) and mineralocorticoids or trihydroxypregnanes (**19**–**21**, **25**–**27**, **38**), and 1.5–5.1-fold for oxysterols (**13**–**15, 41**) while the precursor sterols (**12**, **40**, **42**–**44**) were not detected. One outlier was androstadieneone (**32**), with a 7-fold sensitivity increase with the NH_4_F mobile phase. The pregnenolone derivatives (**16**–**18**, **31**, and the “miscellaneous” steroids **29**–**30**, **34**–**37**) presented an interesting exception to this rule, generally showing lower base peak intensity, i.e., 0.1–0.9-fold that of the formic acid mobile phase, except 17β-OH methyl-Δ^5^-androstene-3β-ol (**35**) (2.9-fold increase).

**Table 3:** Comparison of Mobile Phase Additives in HESI^**+**^ ^a^

		HCO_2_H		0.3 mM NH_4_F		
	Steroid, calc. MH^+^	Base peak, *m*/*z*	Intensity	Base peak, *m*/*z*	Intensity	Ratio NH_4_F/HCOOH
**Estrogens**						
**1**	17β-Estradiol, *m*/*z* 273.1849	**255.1735**	9.2 × 10^4^	**255.1737**	1.6 × 10^4^	0.2
**2**	Estrone, *m*/*z* 271.1693	**271.1684**	**1.7 × 10^5^**	288.1952*	**1.9 × 10^5^**	1.1
**3**	Estriol, *m*/*z* 289.1798	**288.1713**	**1.9 × 10^4^**	306.2064*	**3.4 × 10^5^**	18
**Androgens**						
**4**	Androstenedione, *m*/*z* 287.2006	287.2009	2.4 × 10^7^	287.1989	4.8 × 10^7^	2.0
**5**	Testosterone, *m*/*z* 289.2162	289.2160	2.3 × 10^7^	289.2148	6.6 × 10^7^	2.9
**6**	6β-OH testosterone, *m*/*z* 305.2111	305.2119	2.5 × 10^6^	305.2101	1.0 × 10^7^	4.0
**7**	5α-Dihydrotestosterone, *m*/*z* 291.2319	291.2325	6.8 × 10^5^	291.2307	8.0 × 10^5^	1.2
**8**	19-Nortestosterone, *m*/*z* 275.2006	275.2010	1.6 × 10^7^	275.1990	4.3 × 10^7^	2.7
**9**	19-Norandrostenedione, *m*/*z* 273.1849	273.1849	2.2 × 10^7^	273.1837	4.0 × 10^7^	1.8
**10**	19-OH androstenedione, *m*/*z* 303.1955	303.1959	1.2 × 10^7^	303.1937	3.8 × 10^7^	3.2
**11**	11β-OH androstenedione, *m*/*z* 303.1955	303.1959	1.3 × 10^7^	303.1940	4.4 × 10^7^	3.4
**32**	Androstadieneone, *m*/*z* 271.2056	271.2057	8.9 × 10^6^	271.2046	6.2 × 10^7^	7.0
**33**	Androstadienol, *m*/*z* 273.2213	255.2106	6.4 × 10^5^	273.2205	2.0 × 10^6^	3.1
**Sterols**						
**12**	Cholesterol, *m*/*z* 387.3621	**369.3519**	**2.6 × 10^5^**	**ND**		N/A
**13**	22(*R*)-OH cholesterol, *m*/*z* 403.3571	367.3361	1.2 × 10^5^	385.3447	5.6 × 10^5^	4.7
**14**	20(*R*)-,22(*R*)-(OH)_2_ cholesterol, *m*/*z* 419.3520	383.3313	3.4 × 10^5^	383.3297	5.2 × 10^5^	1.5
**15**	17(*R*)-,20(*R*)-(OH)_2_ cholesterol, *m*/*z* 419.3520	383.3308	9.3 × 10^5^	365.3188	4.7 × 10^6^	5.1
**40**	24,25-Dihydrolanosterol, *m*/*z* 429.4091	**ND**		**ND**		N/A
**41**	14-CDO dihydrolanosterol, *m*/*z* 444.3946	**426.3843**	**1.9 × 10^4^**	**426.3834**	**7.6 × 10^4^**	**4.0**
**42**	FF-MAS, *m*/*z* 413.3778	**ND**		**395.3663**	**7.2 × 10^4^**	N/A
**43**	β-Sitosterol, *m*/*z* 415.3934	**ND**		**ND**		N/A
**44**	Ergosterol, *m*/*z* 397.3465	**ND**		**ND**		N/A
**Pregnenolone derivatives**						
**16**	Pregnenolone, *m*/*z* 317.2475	299.2369	8.1 × 10^5^	317.2459	1.0 × 10^5^	0.1
**17**	3-OAc pregnenolone, *m*/*z* 359.2581	357.2421	7.3 × 10^5^	ND		N/A
**18**	Dehydroepiandosterone, *m*/*z* 289.2162	271.2061	3.9 × 10^5^	289.2142	4.2 × 10^4^	0.1
**31**	17α-OH pregnenolone, *m*/*z* 331.2268	ND		ND		N/A
**Progesterone derivatives**						
**22**	Algestone, *m*/*z* 347.2217	347.2222	7.7 × 10^6^	347.2194	3.1 × 10^7^	4.0
**23**	11-OH progesterone, *m*/*z* 331.2268	331.2267	1.9 × 10^7^	331.2254	6.8 × 10^7^	3.6
**24**	17α-OH progesterone, *m*/*z* 331.2268	331.2272	3.4 × 10^7^	331.2256	8.9 × 10^7^	2.6
**39**	Progesterone, *m*/*z* 315.2319	315.2327	4.0 × 10^7^	315.2305	1.1 × 10^8^	2.8
**Glucocorticoid**						
**25**	Hydrocortisone, *m*/*z* 363.2166	363.2172	1.3 × 10^7^	363.2143	4.6 × 10^7^	3.5
**Mineralocorticoid derivatives**						
**19**	21-OH progesterone, *m*/*z* 331.2268	331.2273	2.0 × 10^7^	331.2258	5.9 × 10^7^	3.0
**20**	21-OAc progesterone, *m*/*z* 373.2373	373.2378	3.9 × 10^7^	373.2351	1.1 × 10^8^	2.8
**21**	Aldosterone, *m*/*z* 361.2010	343.1906	9.1 × 10^6^	343.1887	2.1 × 10^7^	2.3
**26**	3β-OH-preg-5-ene-20,21-diol, *m*/*z* 335.2581	333.2432	3.5 × 10^5^	333.2405	6.0 × 10^5^	1.7
**27**	[3,20-*d*_2_] 3β-OH-preg-5-ene-20,21-diol, *m*/*z* 337.2706	334.2488	4.4 × 10^5^	301.2481	2.1 × 10^6^	4.8
**38**	Corticosterone, *m*/*z* 347.2217	347.2223	6.0 × 10^6^	347.2207	2.9 × 10^7^	4.8
**Miscellaneous steroids**						
**28**	3-Keto-4-etiocholenic acid, *m*/*z* 317.2111	317.2119	2.2 × 10^7^	317.2100	7.5 × 10^7^	3.4
**29**	Etienic acid, *m*/*z* 319.2268	301.2162	2.2 × 10^5^	317.2098	9.7 × 10^4^	0.4
**30**	24-Bisnor-5-cholenic acid-3β-ol, *m*/*z* 347.2581	329.2477	4.8 × 10^5^	329.2458	7.7 × 10^4^	0.2
**34**	Androst-5-ene-3β,17-diol, *m*/*z* 291.2319	273.2211	4.6 × 10^5^	291.2306	2.0 × 10^5^	0.4
**35**	17β-OH methyl-Δ^5^-androstene-3β-ol, *m*/*z* 305.2475	287.2372	5.9 × 10^5^	269.2256	1.7 × 10^6^	2.9
**36**	*d*_2_-17β-OH methyl-Δ^5^-androstene-3β-ol, *m*/*z* 307.2601	289.2497	3.0 × 10^5^	289.2480	1.7 × 10^4^	0.06
**37**	17β-Formyl-Δ^5^-androstene-3β-ol, *m*/*z* 303.2319	301.2166	1.9 × 10^6^	285.2201	1.8 × 10^6^	0.9

a Values recorded in plain text were determined at 100 μM steroid concentration, while values in bolded text were determined at 500 μM (i.e., 10 μL injected = 1 or 5 nmol). Ions denoted with an asterisk (*) are NH_4_^+^ adducts. ND: not detected (response <1 × 10^4^). N/A: not applicable.

The use of ESI^–^ MS has been reported to increase the detection sensitivity of estrogens and acidic steroids.^35–37^ For estrogen analysis, the use of a basic mobile phase (e.g., supplemented with NH_4_OH) can facilitate deprotonation of the phenolic hydroxyl moiety for ESI^–^ analysis, though significant ESI^–^sensitivity gains have been reported using NH_4_F.^38–40^ We tested whether the NH_4_F (0.3 mM) mobile phase facilitated HESI^–^ analysis of estrogens (**1**–**3**) and three acidic steroids (**28**–**30**) and observed mixed results. The ratio of base peak intensity (HESI^–^ /HESI^+^, both conducted in the NH_4_F (0.3 mM) mobile phase) was 77 (**1**), 10 (**2**), and 0.8 (**3**) for the estrogens and 0.4 (**28**), 66 (**29**), and 95 (**30**) for three analyzed carboxy-steroids (Supporting Figures S48–S53). HESI^–^ was optimal for two estrogens (**1** and **2**) and two carboxy-steroids (**29** and **30**) but was roughly equally sensitive for one molecule in each class (**3** and **28**), though the effect was observed to vary >100-fold among the three molecules in each class.

## Discussion

A variety of MS-based approaches have been used in the detection and quantification of steroids and sterols over the past 60 years. Early work predating the advent of electrospray ESI-MS employed electron impact GC-MS,^3,41,42^ while more recent methods utilize ESI^43^ or APCI (MS) approaches, in many cases involving a tandem (MS/MS) approach. Recently, HESI MS has been reported to be more useful than standard ESI MS for steroids.^31,32^ Some investigators have instead relied on chemical derivatization to increase sensitivity (i.e., dansyl chloride^44^ or hydroxylamine^45^), while others have found that careful selection of the mobile phase additive (i.e., NH_4_OH, NH_4_F, or NH_4_OAc) can give comparable or enhanced sensitivity relative to chemical derivatization. In our own experience, steroid/sterol sensitivity has been significantly enhanced with chemical (dansyl) derivatization,^11,13,16^ which is practical with a subset of steroids (those bearing carbonyl moieties).

We compared the detection sensitivity of 44 steroids using both APCI^+^ and ESI^+^ MS approaches (Table 2). Δ^4^ steroids generally performed best on ESI^+^ (up to a maximum factor of 7.8-fold), while Δ^5^ steroids and sterols generally performed better on APCI^+^ (up to a maximum factor of >137-fold), except for two trihydroxypregnanes (**26**, **27**). Estrogens did not show a clear trend, demonstrating either minimal difference or substantial preference for APCI (by a factor of 0.2–14-fold). We, like others, observed a general increase in sensitivity with the use of NH_4_F as a mobile phase additive for ESI^+^ MS, with the exception of some Δ^5^ steroids (Table 3), as well as gains in ESI^–^ MS for some estrogens and acidic steroids (**1**, **2**, **29**, **30**).^35–37^

While we did not compare the effect of this mobile phase on APCI-MS, another group has reported significant sensitivity increases with a very high NH_4_F concentration (1 M) in an SFC-APCI MS approach.^35^ These higher concentrations were necessary in APCI MS analyses, but lower concentrations were optimal in the ESI MS approach (1 mM).^35^ This level compares to LC-ESI MS approaches, where a similarly lower additive concentration (6 mM)^44^ has been used (in our own approach we used 0.3 mM). We cannot exclude the possibility that the sensitivity gain from the NH_4_F additive would increase at higher concentrations.

While we often observed that APCI generated more ions than HESI (generally as 2*n* or 18*n* amu losses, Figure 3), the sensitivity advantage of HESI over APCI that was observed for many steroids is not inherently due to this effect (i.e., that HESI generated fewer more intense ions while APCI generated more ions with lower intensity, thus “diluting” the signal). Rather, examples with the mineralocorticoids (e.g., corticosterone (**38**), Table 2) often revealed the same base peak generated from both methods with roughly the same ions but with an increase in base peak signal in HESI MS analysis. Further, the trend was maintained by summing the intensities of the ions in Table 1 (generating a figure for the total ion intensity) for each method, confirming the effect.

In the course of our sterol analysis with APCI MS, we previously observed 2*n* amu losses but did not give much attention to the matter. In fact, this phenomenon was evidently present in some of our earlier APCI work with OH-androgens to varying degrees, e.g., 6β-OH testosterone.^23^ We did not, however, see any evidence for this phenomenon for 1β-OH testosterone using a time-of-flight instrument (200 °C).^46^ More recently, discordance in structural assignments between NMR and *m*/*z* spectra in our synthetic work with oxysterols caused us to consider this phenomenon more seriously, in that dominant 2*n* amu losses on NaBH_4_-treated sterols were initially mistaken as the precursor ion MH^+^ of the starting material (rather than MH^+^-2 of the product), causing ambiguity as to whether the reduction (which had supposedly gone to completion, per the NMR spectrum) was successful (Scheme 1).

A total of 36 of the 44 tested sterols showed losses of 2*n* amu in APCI MS analysis (17/22 Δ^5^ steroids, 17/19 Δ^4^ steroids, and 2/3 estrogens). These losses were prominent ions in most cases but were especially dominant in the Δ^5^ steroids and sterols, in which the precursor MH^+^ was either observed as a minor ion or (more frequently) not detected at all, constituting the base peak in 7/22 cases (Figure 1). These results compare to HESI MS, in which 2*n* amu losses were detected (generally weakly) in 9/44 cases but constituted the base peak in 3/9 of those. The nature of these losses was confirmed via the analysis of a deuterated steroid. When *d*_0_-17β-OH methyl-Δ^5^-androstene-3β-ol (**35**) was deuterated (*d*_2_-17β-OH methyl-Δ^5^-androstene-3β-ol, **36**, Figure 2A), the prominent ion distribution shifted from MH^+^-2, MH^+^-4, and MH^+^-6 (*d*_0_, Figure 2B) to MH^+^-2, MH^+^-5, and MH^+^-7 (*d*_2_, Figure 2C). The presence of MH^+^-3 or MH^+^-5 ions was rarely observed in our APCI analysis of steroids, except for with deuterated steroids. The prominence of the MH^+^-5 ion in *d*_2_-17β-OH methyl-Δ^5^-androstene-3β-ol (**36**) confirmed that 2-electron oxidations observed in APCI MS analysis can be attributed to carbinol oxidations, in that deuterium could only be lost at C-17, and the dominance of the MH^+^-2 ion may suggest that a single 2-electron oxidation may be most prevalent at C-3, potentially owing to the increased stability of a C-3 ketone over an C-20 aldehyde.

One interesting observation was that some steroids showed 2*n* and 18*n* amu losses exceeding the number of available hydroxyl groups (i.e., that *n* > the number of −OH groups) suggesting that not every 2*n* loss could be explained by carbinol oxidation. Particularly interesting cases are the Δ^4^ steroids, some of which lack any available hydroxyl moiety (i.e., **4**, **28**, **32**, **39**) but show 2*n* losses. While these losses are generally minor peaks (maximum of 5–18% of the base peak intensity), they might be rationalized via a mechanism involving the loss of allylic hydride and thus generating a carbocation as has been suggested recently.^33^ In addition to a mechanism of carbinol oxidation and subsequent protonation of the carbonyl, the loss of allylic hydride presents an alternative mechanistic route to a positively charged ion detected as M^+^-H (mathematically equivalent to MH^+^-2H).

Our attempts to distinguish between the contributions of either mechanism began with reduction of the Δ^5^ bond of DHEA (to yield *allo*-DHEA), with the rationale being that if the formation of MH^+^-2*n* ions was dependent on the loss of allylic hydride, then elimination of the allylic site (via saturation of the Δ^5^ bond) should prevent the formation of MH^+^-2*n* ions. If such a mechanism was not present in our analyses, then *allo*-DHEA and DHEA would be expected to show very similar ion distributions (different *m*/*z* values, but similar patterns of MH^+^-2*n* and MH^+^-(*n* × H_2_O) ions). We observed that the ion distribution (of *allo*-DHEA) shifted in the direction of MH^+^-(*n* × H_2_O) and revealed the MH^+^ ion (relative to DHEA), and that the base peak was still a MH^+^-2*n* ion (Supporting Figure S45). As reduction of the Δ^5^ bond did not preclude the formation of MH^+^-2*n* ions but significantly shifted the ion distribution toward alternative ionization mechanisms (generation of MH^+^-(*n* × H_2_O) and MH^+^ ions), a mechanism involving loss of allylic hydride is likely present in addition to carbinol oxidation in the generation of MH^+^-2*n* ions in our APCI analyses, i.e., the relative contribution of the allylic hydride mechanism to ionization in DHEA was diverted to alternative ionization mechanisms in *allo*-DHEA, detected as a significant increase in the proportion of MH^+^-(*n* × H_2_O) ions observed. Given the retained prominence of the MH^+^-2*n* ions, we consider that the contribution of an allylic hydride loss mechanism is likely minor in relation to the carbinol oxidation mechanism. We again note the presence of several steroids (i.e., **4**, **28**, **32**, **39**) in our analysis that did not have any carbinol moieties in their structures but still demonstrated significant MH^+^-2*n* ions, an ion which is perhaps best rationalized via a loss of allylic hydride mechanism.

Further efforts to characterize the MH^+^-2 ions were conducted using targeted LC-MS/MS fragmentation analysis of compound **37**, which demonstrated prominent MH^+^-2 ions in untargeted analyses in both APCI^+^ and HESI^+^ MS (Supporting Figure S37). MS/MS fragmentation of the MH^+^-2 ion revealed identical MH^+^-(*n* × H_2_O)-2 product ions in both techniques (Supporting Figure S46), suggesting the precursor MH^+^-2 ion is the same. However, if the route to the MH^+^-2 precursor ion was a carbinol oxidation mechanism, the loss of H_2_O from this ion in the MS/MS fragmentation spectrum is initially difficult to rationalize, as no carbinol is left in the molecule to protonate and leave (as H_2_O) to generate this ion. We considered progesterone (**39**), which lacks a carbinol moiety, and collected MS/MS spectra of the precursor MH^+^ ion, which revealed a product ion (base peak) of MH^+^-H_2_O in the MS/MS fragmentation spectrum (Supporting Figure S46). Thus, the generation of MH^+^-(*n* × H_2_O) ions is not dependent on the availability of hydroxyl moieties in the molecular structure, and the observation of a MH^+^-H_2_O-2 product ion in the fragmentation MS/MS spectrum of **37** does not contradict our carbinol oxidation mechanism, i.e., a MH^+^-2 precursor ion resulting from carbinol oxidation of **37** could still lose H_2_O to generate product ions of MH^+^-H_2_O-2 (as we observed) without bearing a carbinol.

In total, we consider that carbinol oxidation is likely a dominant mechanism in the generation of MH^+^-2 ions, with a minor contribution of allylic hydride loss.

## Conclusions

Our results indicate that the analysis of Δ^4^ steroids was generally optimal with HESI^+^ MS, while APCI^+^ MS was often advantageous for Δ^5^ steroids, with some exceptions. The sensitivity difference rarely exceeded 3-fold in either direction, though sensitivity on HESI^+^ could often be enhanced with the use of NH_4_F as a mobile phase additive (though rarely by a factor >4-fold). The detection of sterols (also Δ^5^ structures), however, was substantially improved with APCI^+^ MS, though analyses with APCI^+^ often generated additional ions (generally −2*n* amu losses) that dominated the *m*/*z* spectrum. While 2*n* amu losses were also observed in HESI^+^ analysis (in 9/44 steroids/sterols), they were most prominent (and problematic) in APCI^+^ analyses (36/44 steroids/sterols). We attribute these losses largely to a carbinol oxidation mechanism, although we provide evidence that loss of allylic hydride is an alternative mechanism, likely minor in contribution, that is also capable of generating these ions. These artifacts may complicate accurate structure assignment, particularly when the precursor ion MH^+^ is observed as a minor or absent ion. In the case of dominant 2*n* amu losses, the MH^+^-18*n* (loss of *n* × H_2_O) ion generally provides a reliable point of reference for structure determination.

Taken together, we present a general guide for conducting LC-MS analyses of the steroid family of molecules, guided by two main properties: (i) Δ^5^ vs Δ^4^ conjugation and (ii) steroid vs sterol. A steroid of Δ^4^ conjugation was generally most sensitively analyzed by HESI-MS complemented by a NH_4_F (0.3 mM) mobile phase additive, while one of Δ^5^ conjugation was generally optimal via APCI-MS (though the advantage was frequently ≤2-fold). Given that APCI-MS typically generates a pattern of MH^+^-2*n* ions that is not frequently observed in HESI-MS, the latter method is suitable for structure elucidation with a minimal loss of sensitivity, which may be necessary in steroid synthesis. Note that the HESI-MS of Δ^5^ steroids should generally exclude the NH_4_F mobile phase additive, which frequently compromised the sensitivity. For sterols (all of Δ^5^ conjugation), APCI-MS was a general necessity, in that many sterols were not detected by HESI-MS, although this effect was much less pronounced with some oxysterols.

## Supplementary Material



## Notes

APCI and HESI UPLC chromatograms and spectra of all analyzed steroids (PDF)
