# Supplementary material for: Mass Spectral Analysis of Sterols and Other Steroids in Different Ionization Modes: Sensitivity and Oxidation Artifacts
Source: J Am Soc Mass Spectrom. 2025 Jun 27;36(8):1702–17. doi: 10.1021/jasms.5c00099 (PMC12333350; doi:10.1021/jasms.5c00099)
Supplement: Supplementary file 1 [file js5c00099_si_001.pdf]

SUPPORTING INFORMATION

Mass Spectral Analysis of Sterols and Other Steroids in Different Ionization Modes: Sensitivity and Oxidation Artifacts

Kevin D. McCarty[1] and F. Peter Guengerich[1, *]

[1]Department of Biochemistry, Vanderbilt University School of Medicine, Nashville, Tennessee 37232-0146, United States

Address correspondence to:
Prof. F. Peter Guengerich
E-mail: f.guengerich@vanderbilt.edu

Table of contents

Figures S1 to **S44.** APCI$^+$ and HESI$^+$ spectra of all analyzed steroids (**1** to **44**) (structures in Scheme 2)….. pp. S2 to S45

Figure S45. APCI$^+$ analysis of DHEA and *allo*-DHEA ………………………………………………………… p. S46

Figure S46. APCI$^+$ and HESI$^+$ targeted LC-MS/MS analysis of **37** ………………………………………… p. S47

Figure S47. APCI$^+$ targeted LC-MS/MS analysis of **39** ……………………………………………...………...… p. S48

Figure S48 to **S53.** HESI$^-$ LC-MS of **1-3**, **28-30** analyzed with a NH_4F mobile phase……………………...…… pp. S49 to S54

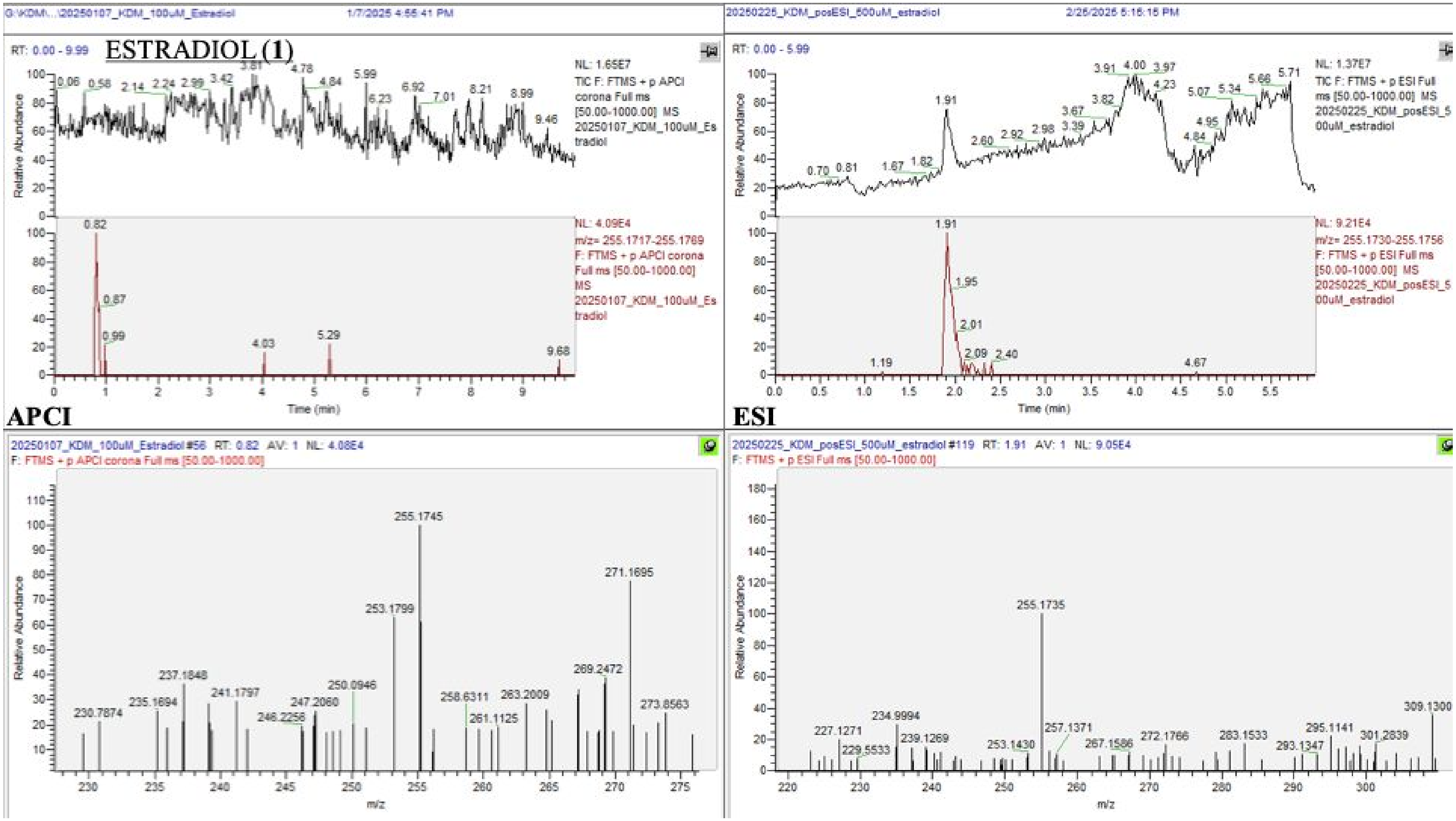

Figure S1. LC-HRMS chromatograms of estradiol (**1**, 500 μM) ionized by APCI (left) and HESI (right).

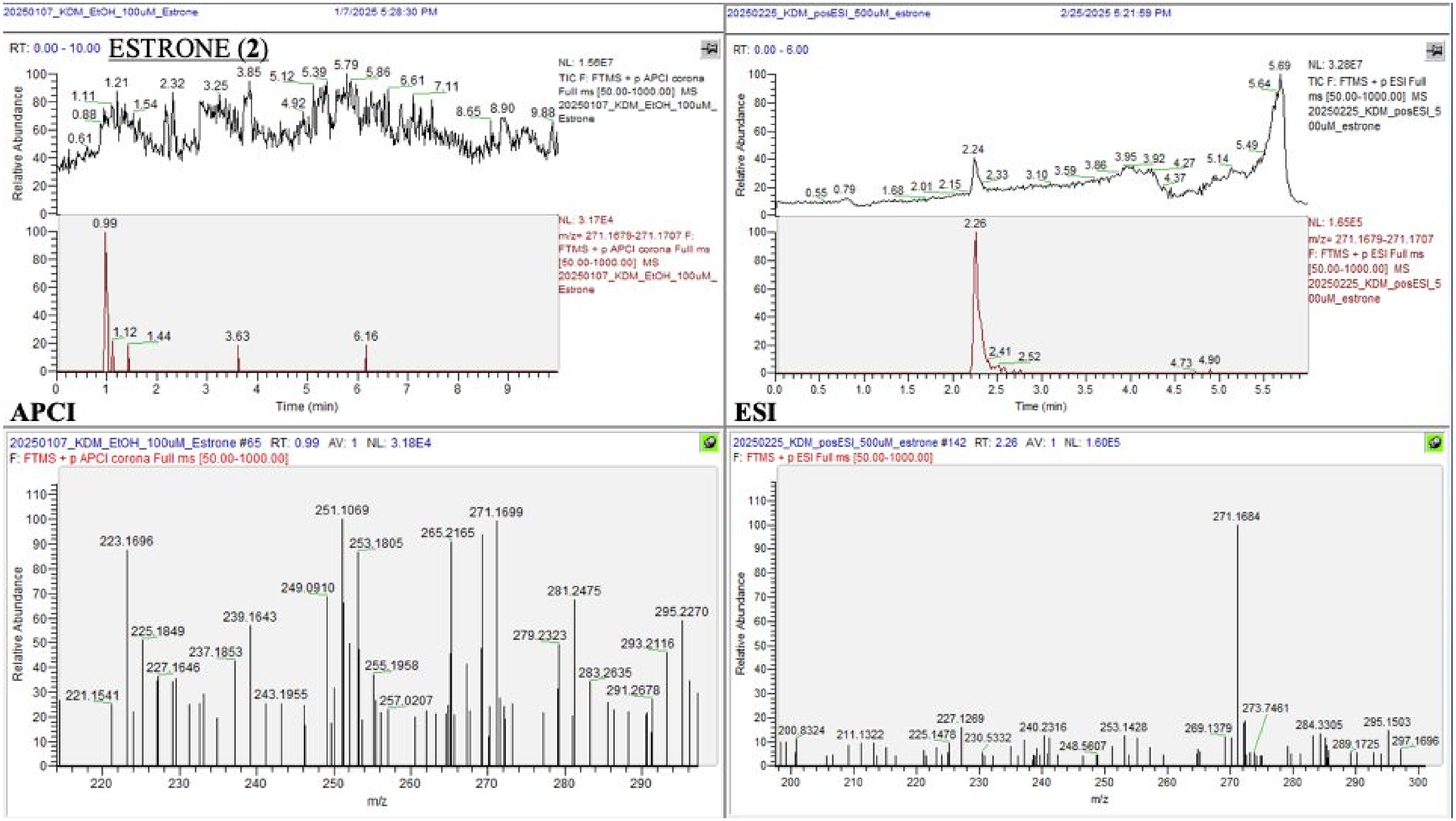

Figure S2. LC-HRMS chromatograms of estrone (**2**, 500 μM) ionized by APCI (left) and HESI (right).

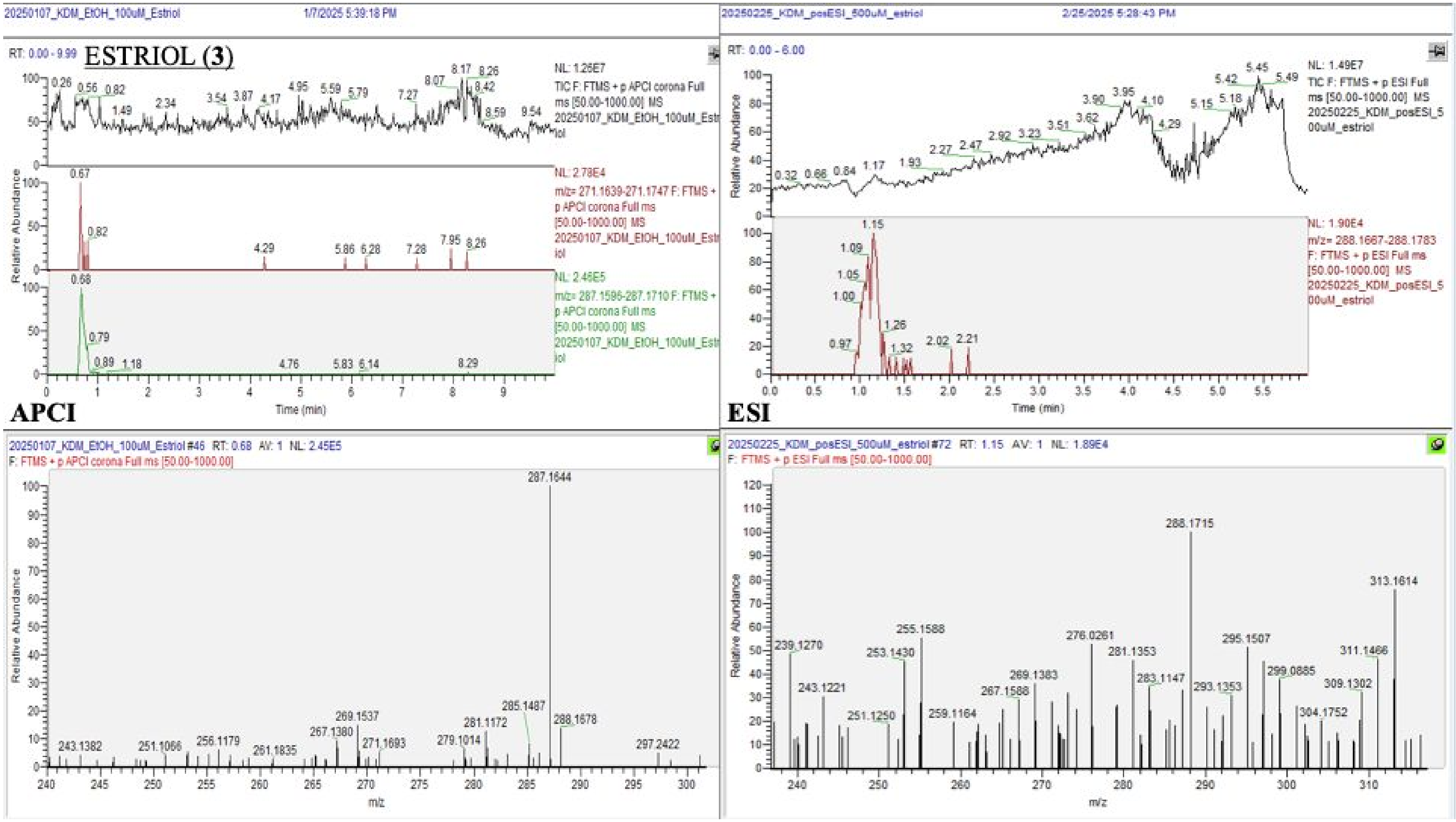

Figure S3. LC-HRMS chromatograms of estriol (**3**, 500 μM) ionized by APCI (left) and HESI (right).

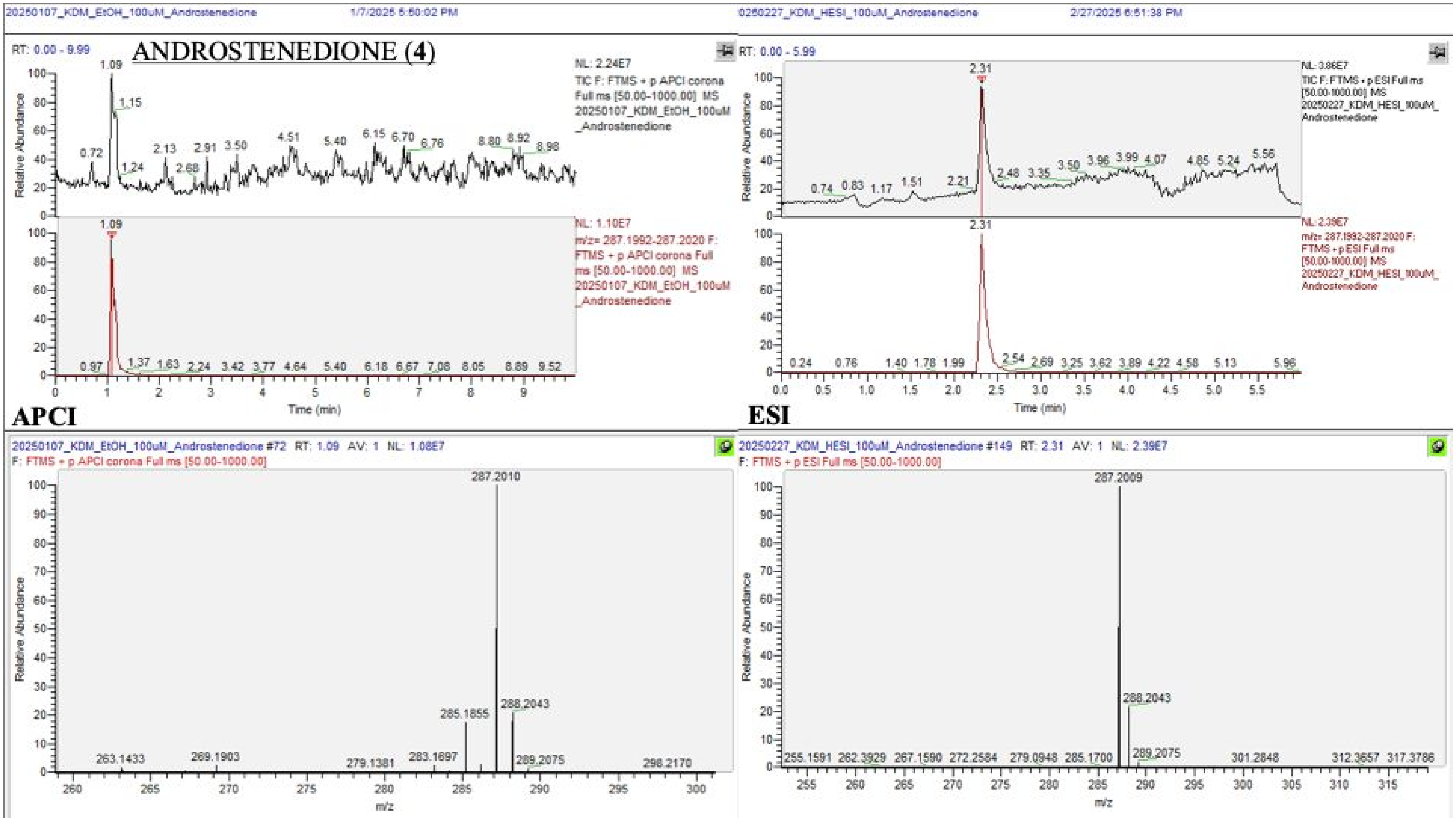

Figure S4. LC-HRMS chromatograms of androstenedione (**4**, 100 µM) ionized by APCI (left) and HESI (right).

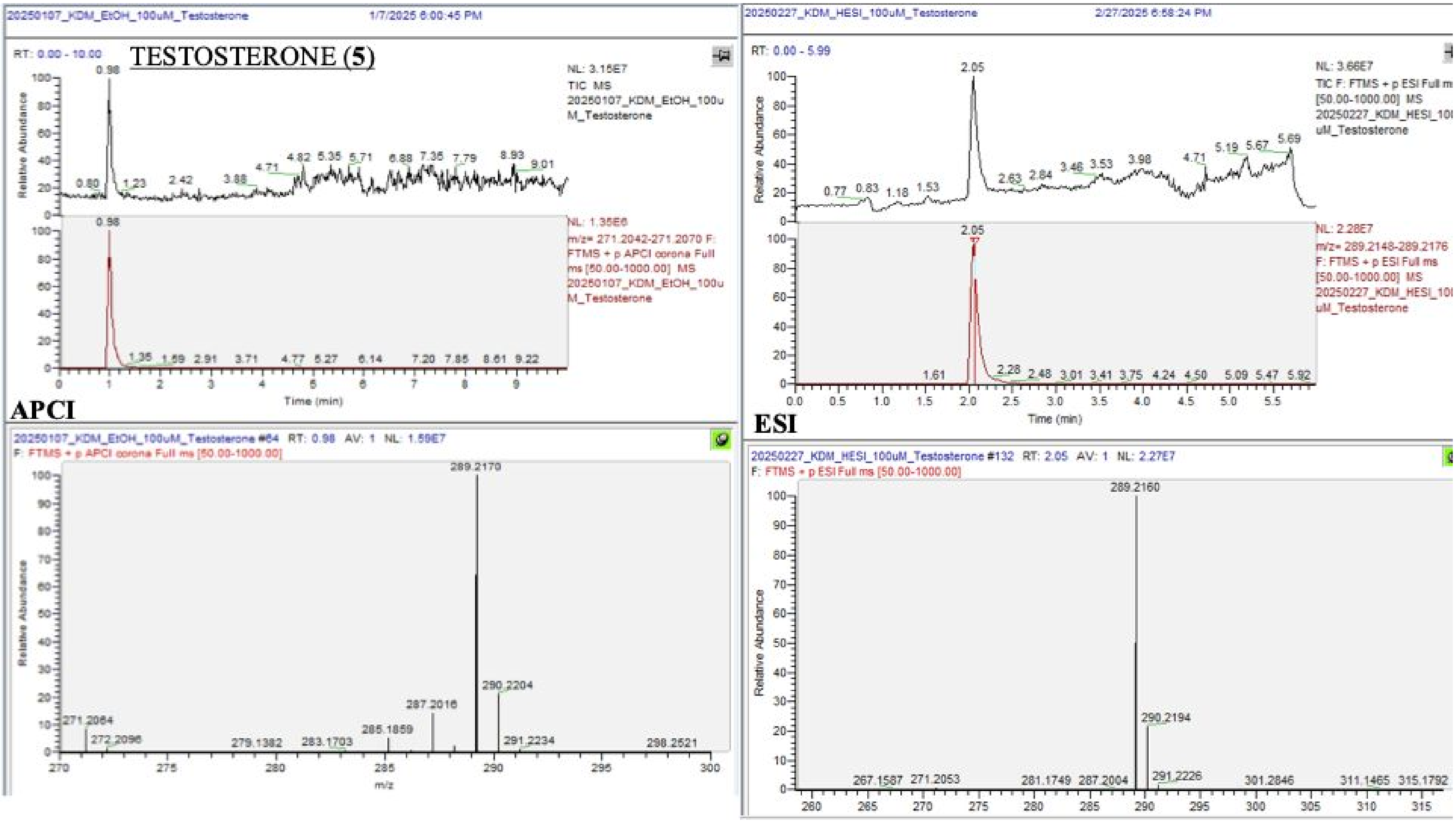

Figure S5. LC-HRMS chromatograms of testosterone (**5**, 100 μM) ionized by APCI (left) and HESI (right).

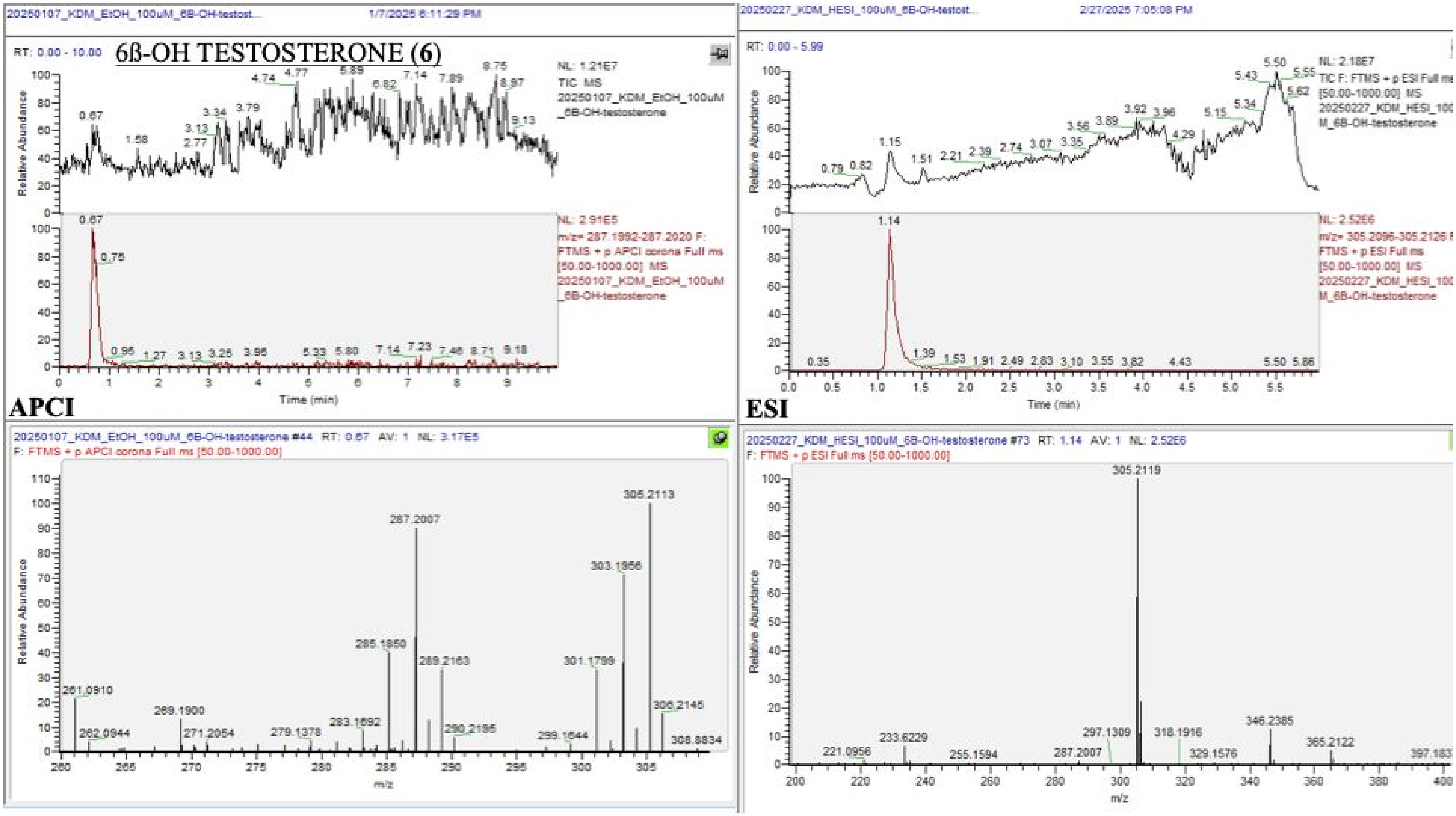

Figure S6. LC-HRMS chromatograms of 6β-OH testosterone (**6**, 100 μM) ionized by APCI (left) and HESI (right).

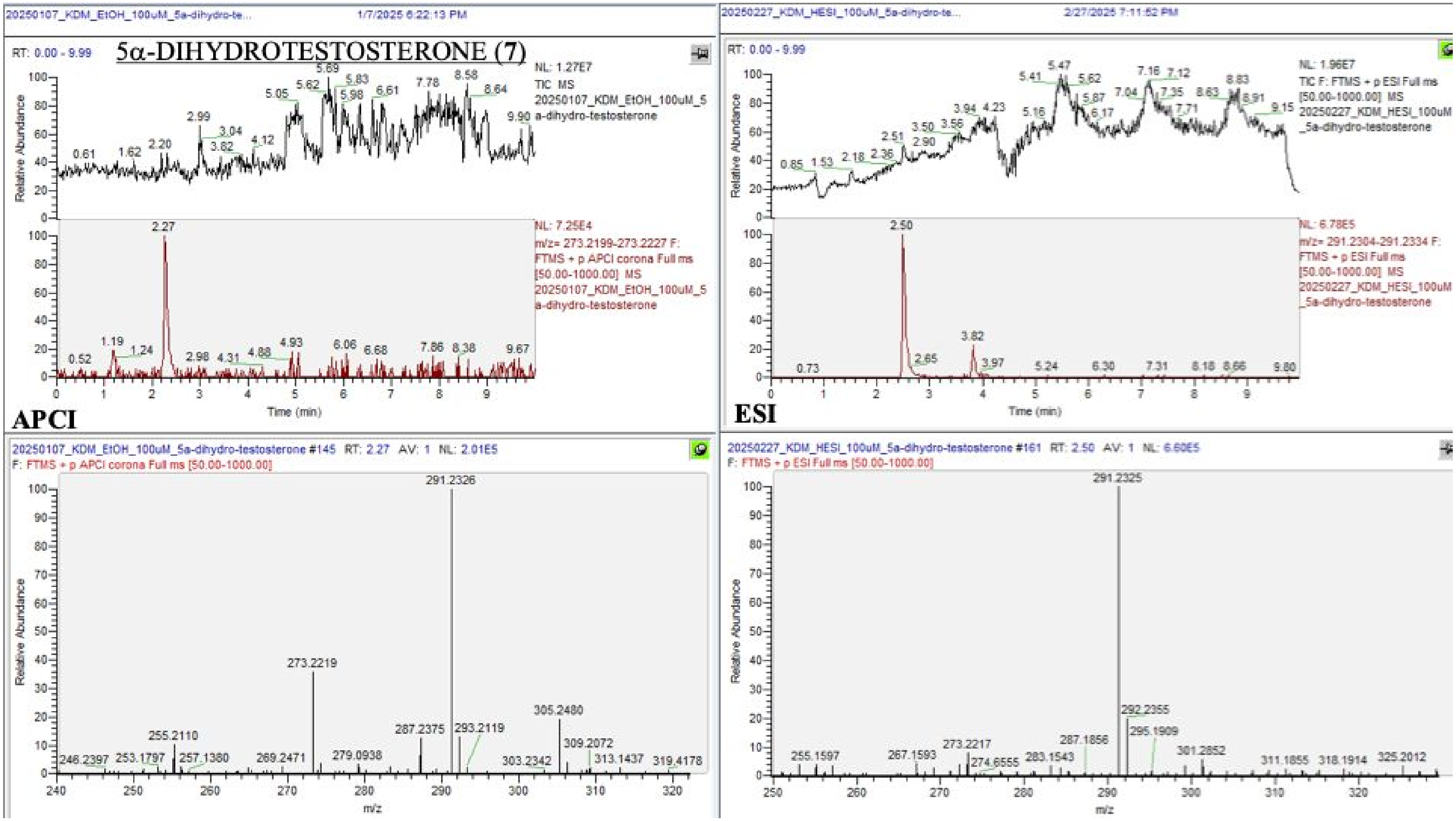

Figure S7. LC-HRMS chromatograms of 5α-dihydrotestosterone (**7**, 100 μM) ionized by APCI (left) and HESI (right).

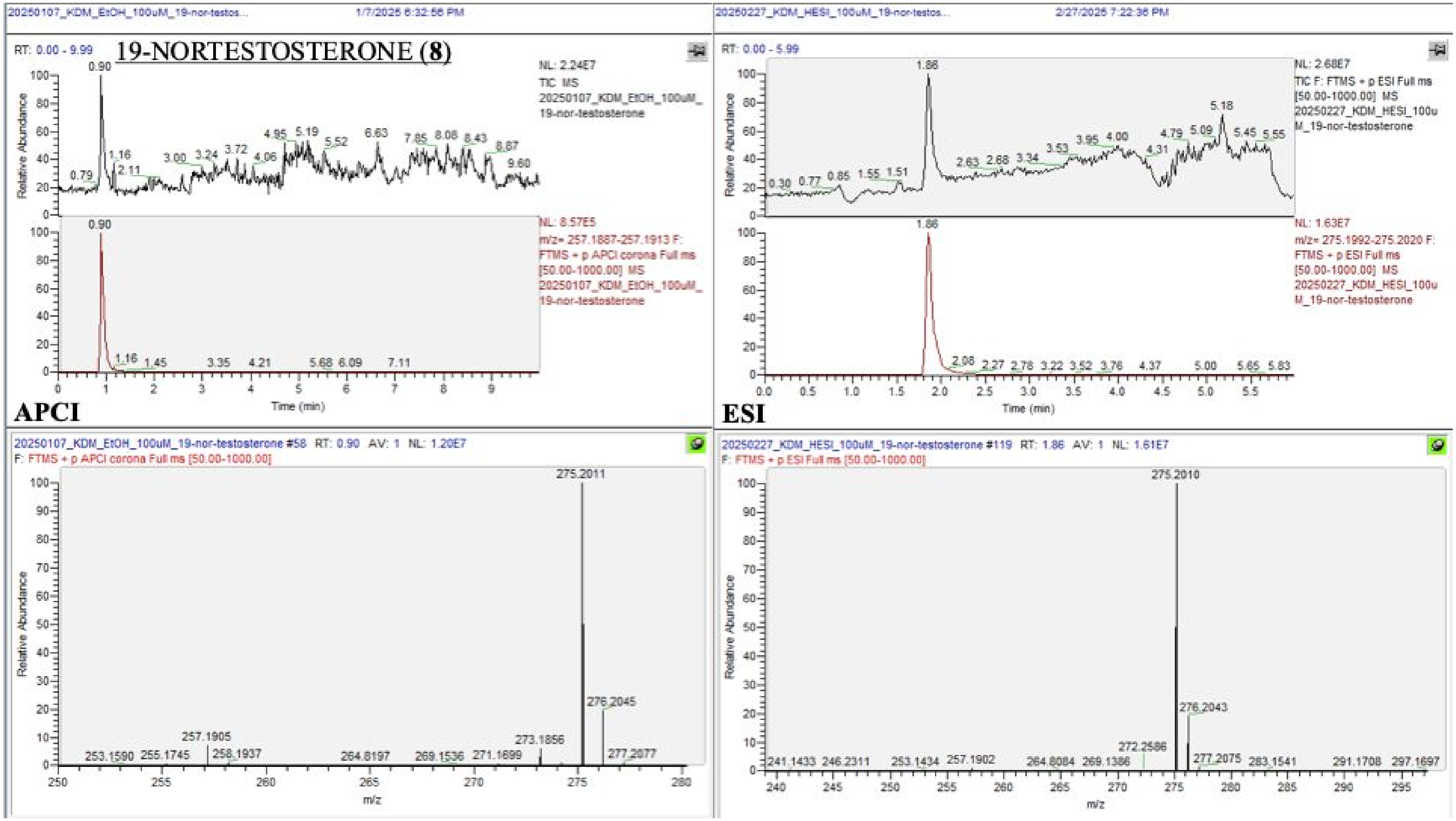

Figure S8. LC-HRMS chromatograms of 19-nortestosterone (**8**, 100 μM) ionized by APCI (left) and HESI (right).

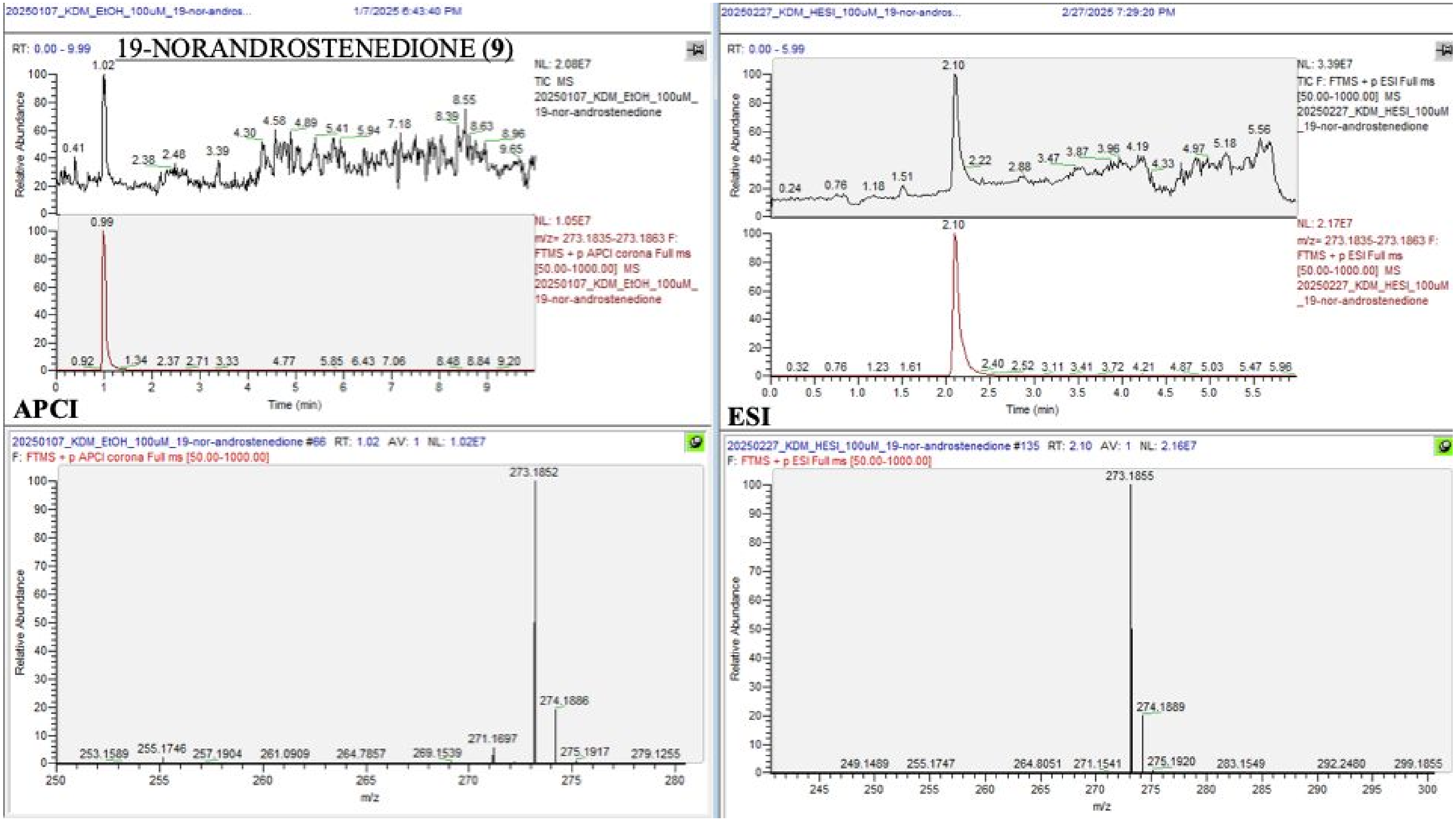

Figure S9. LC-HRMS chromatograms of 19-norandrostenedione (**9**, 100 μM) ionized by APCI (left) and HESI (right).

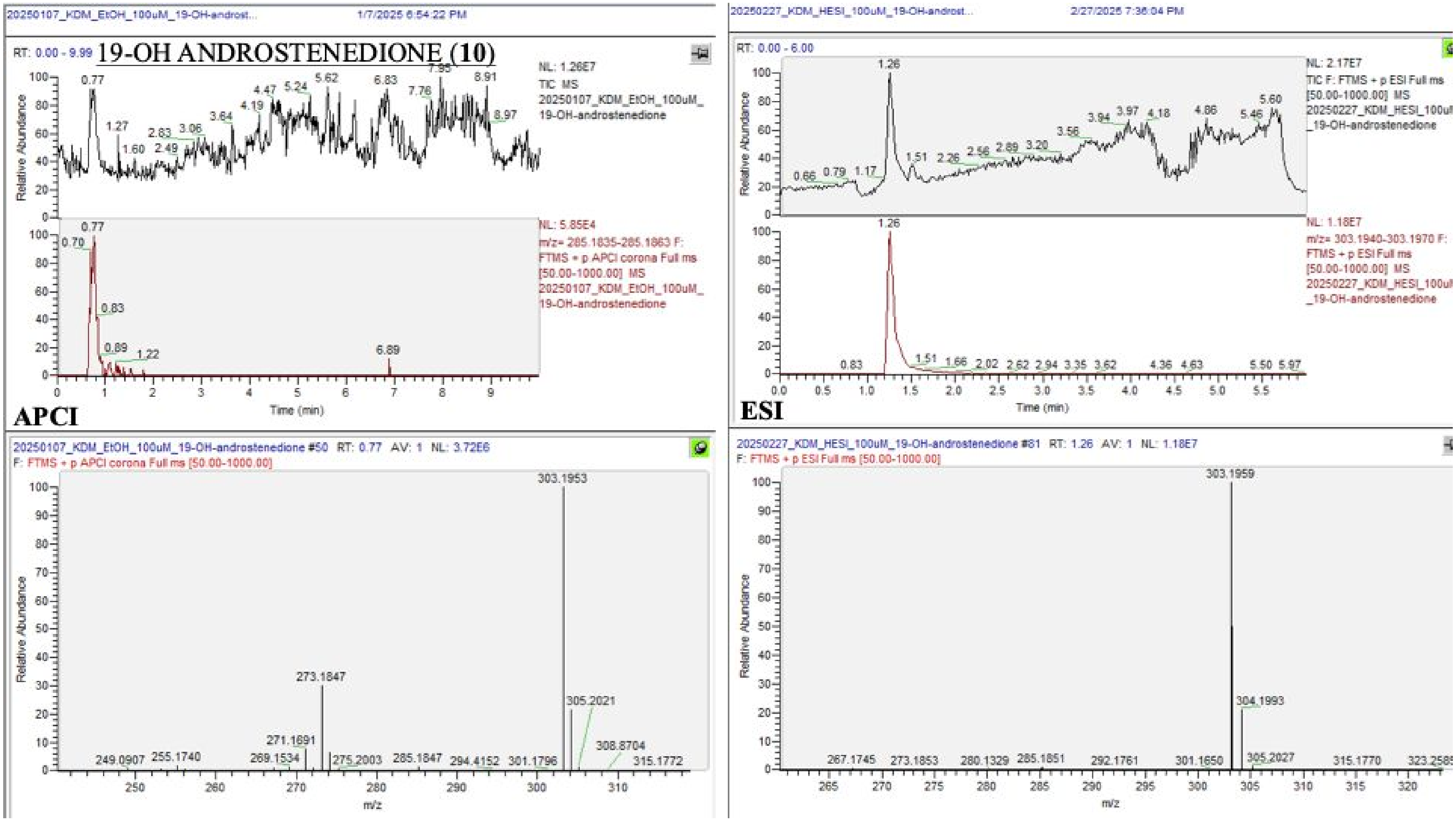

Figure S10. LC-HRMS chromatograms of 19-OH androstenedione (**10**, 100 μM) ionized by APCI (left) and HESI (right).

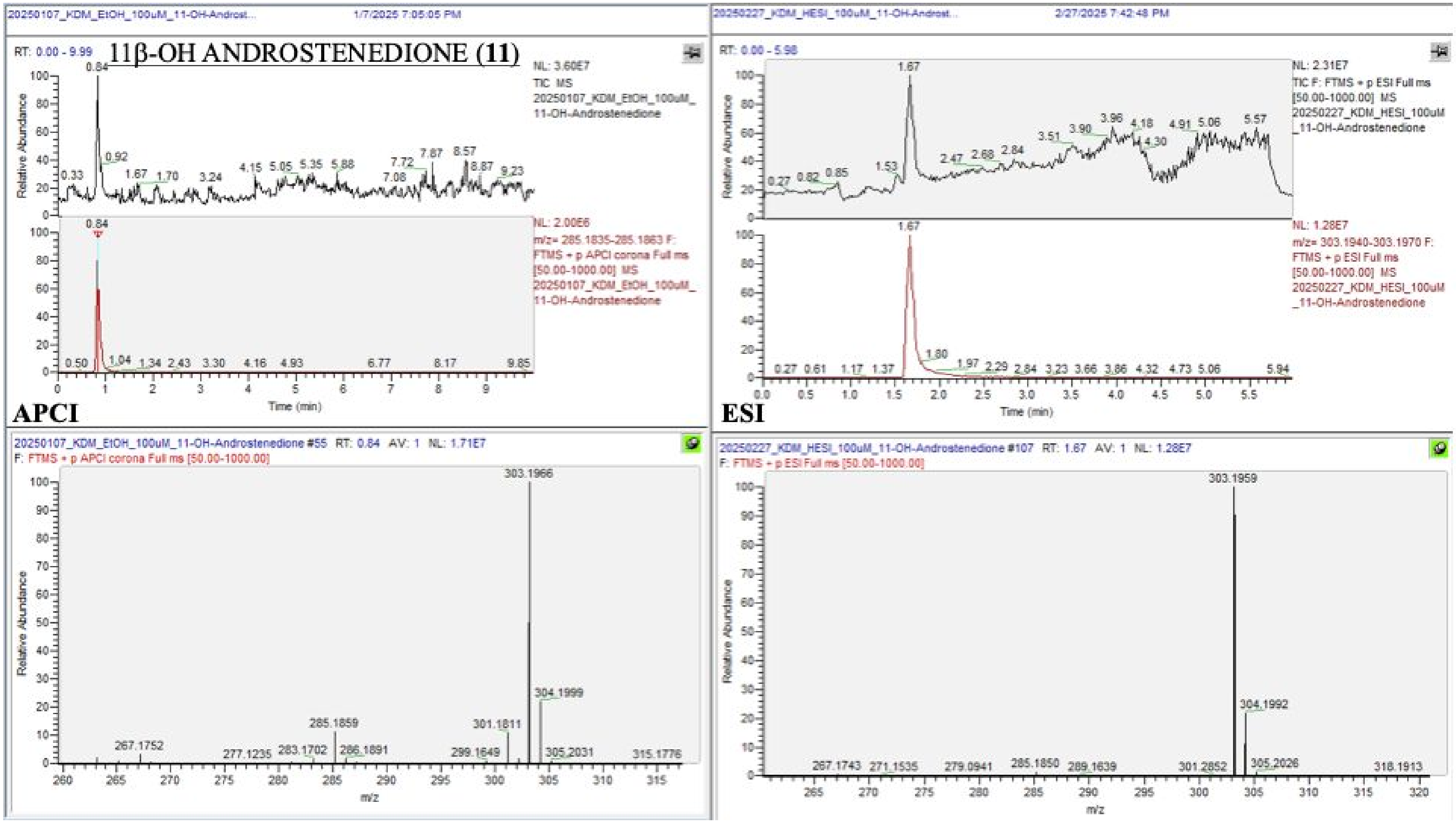

Figure S11. LC-HRMS chromatograms of 11β-OH androstenedione (**11**, 100 μM) ionized by APCI (left) and HESI (right).

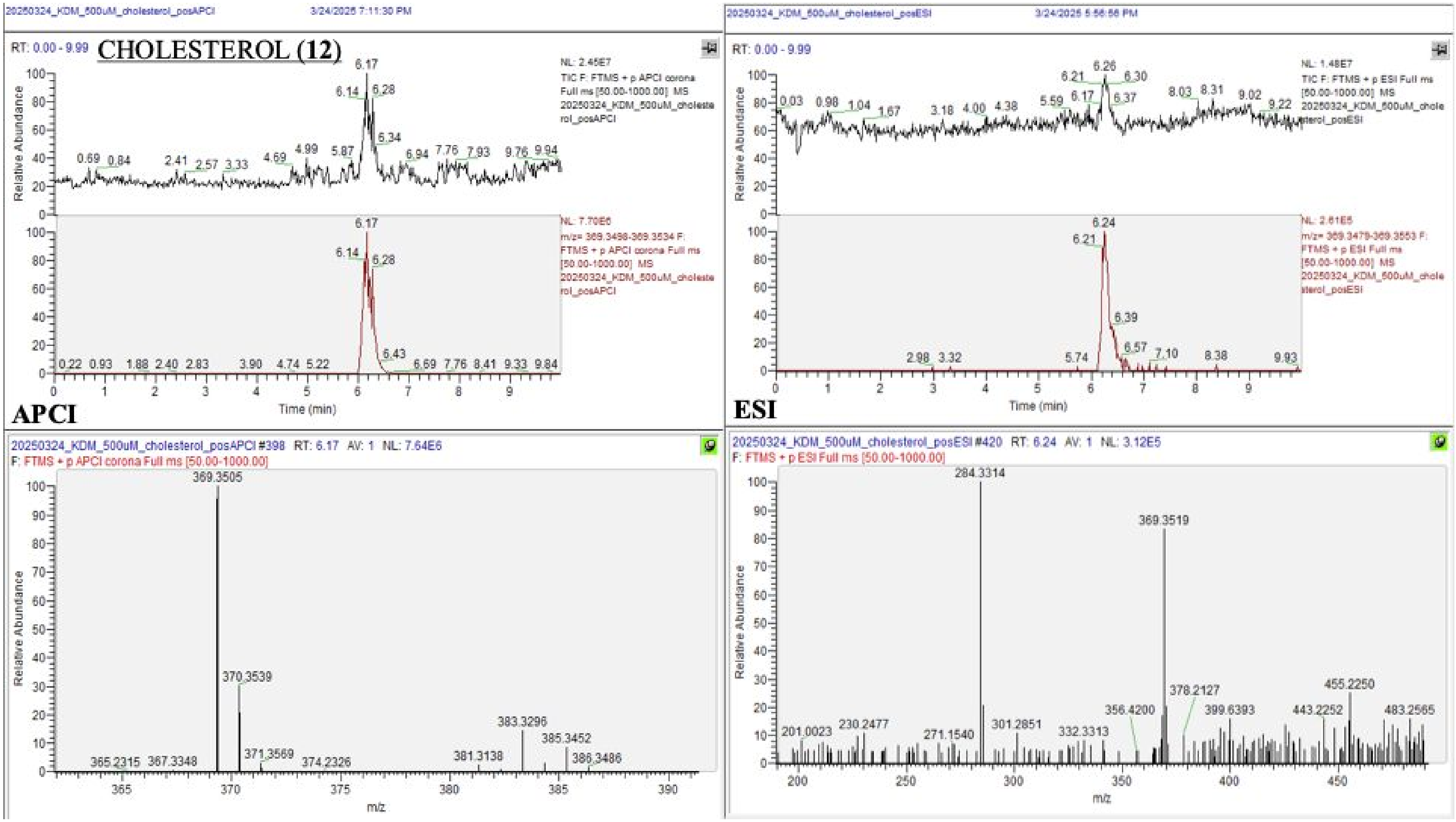

Figure S12. LC-HRMS chromatograms of cholesterol (**12**, 100 μM) ionized by APCI (left) and HESI (right).

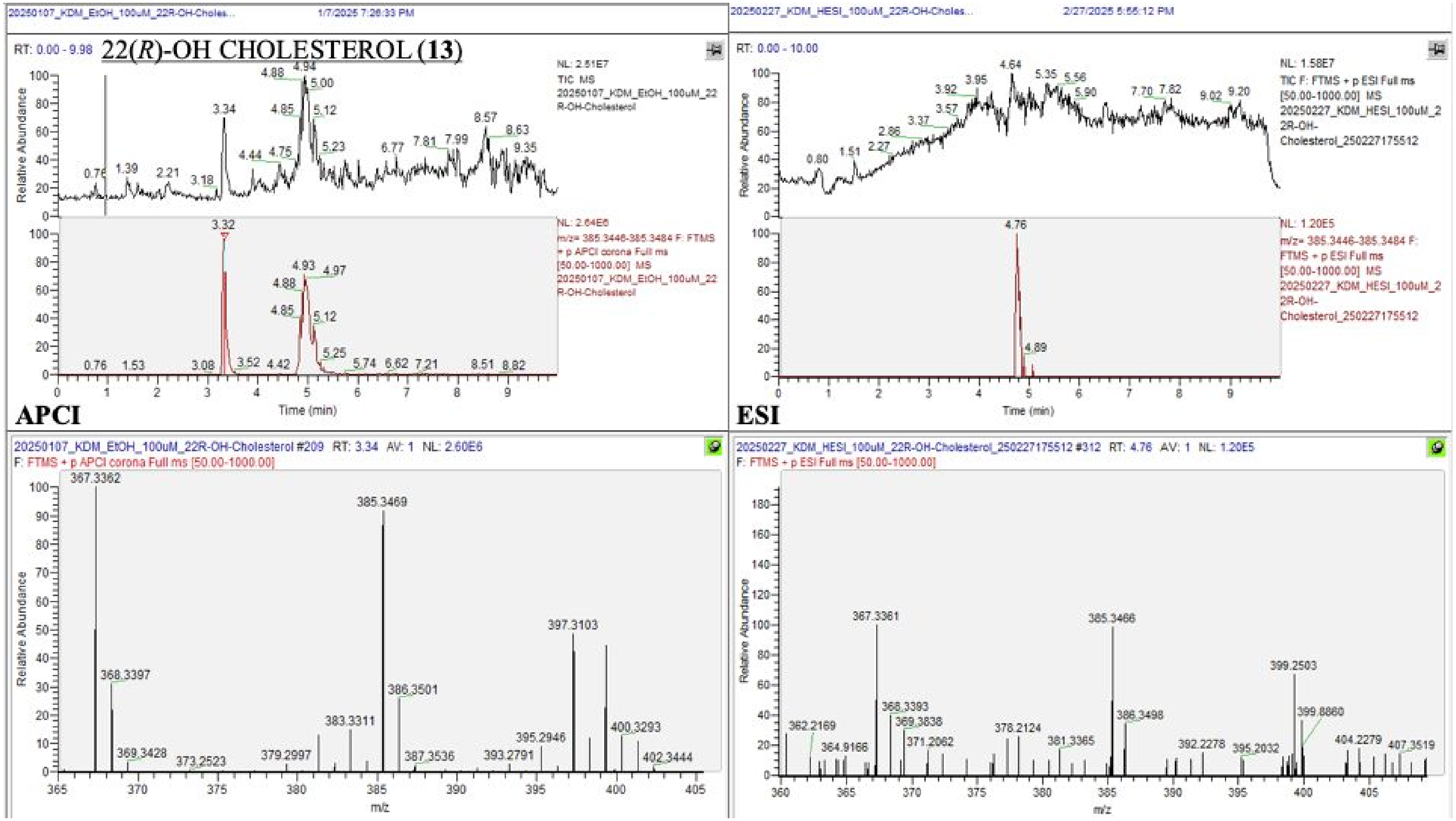

Figure S13. LC-HRMS chromatograms of 22*R*-OH cholesterol (**13**, 100 μM) ionized by APCI (left) and HESI (right).

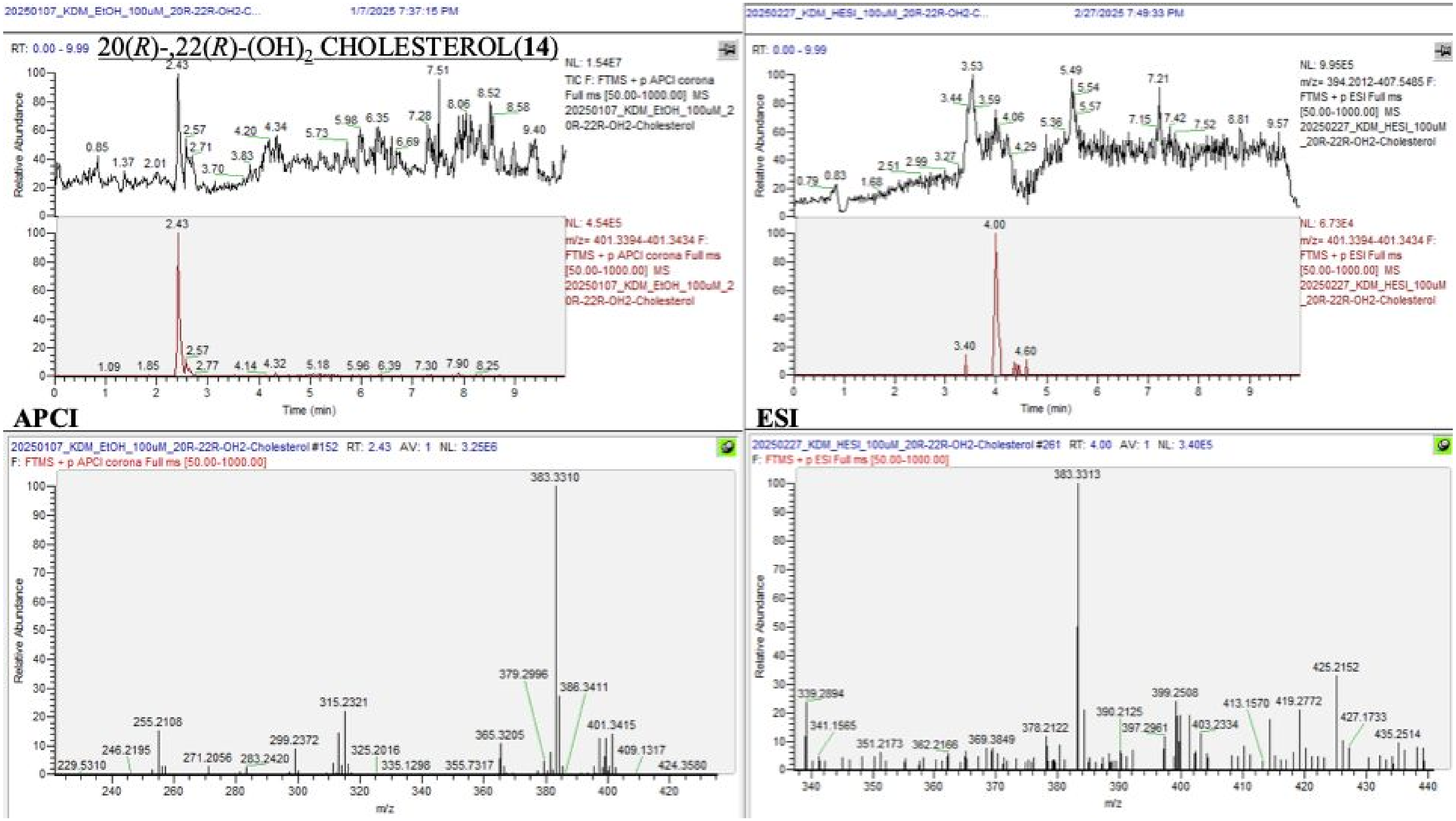

Figure S14. LC-HRMS chromatograms of 20R,22R-$(OH)_2$ cholesterol (**14**, 100 μM) ionized by APCI (left) and HESI (right).

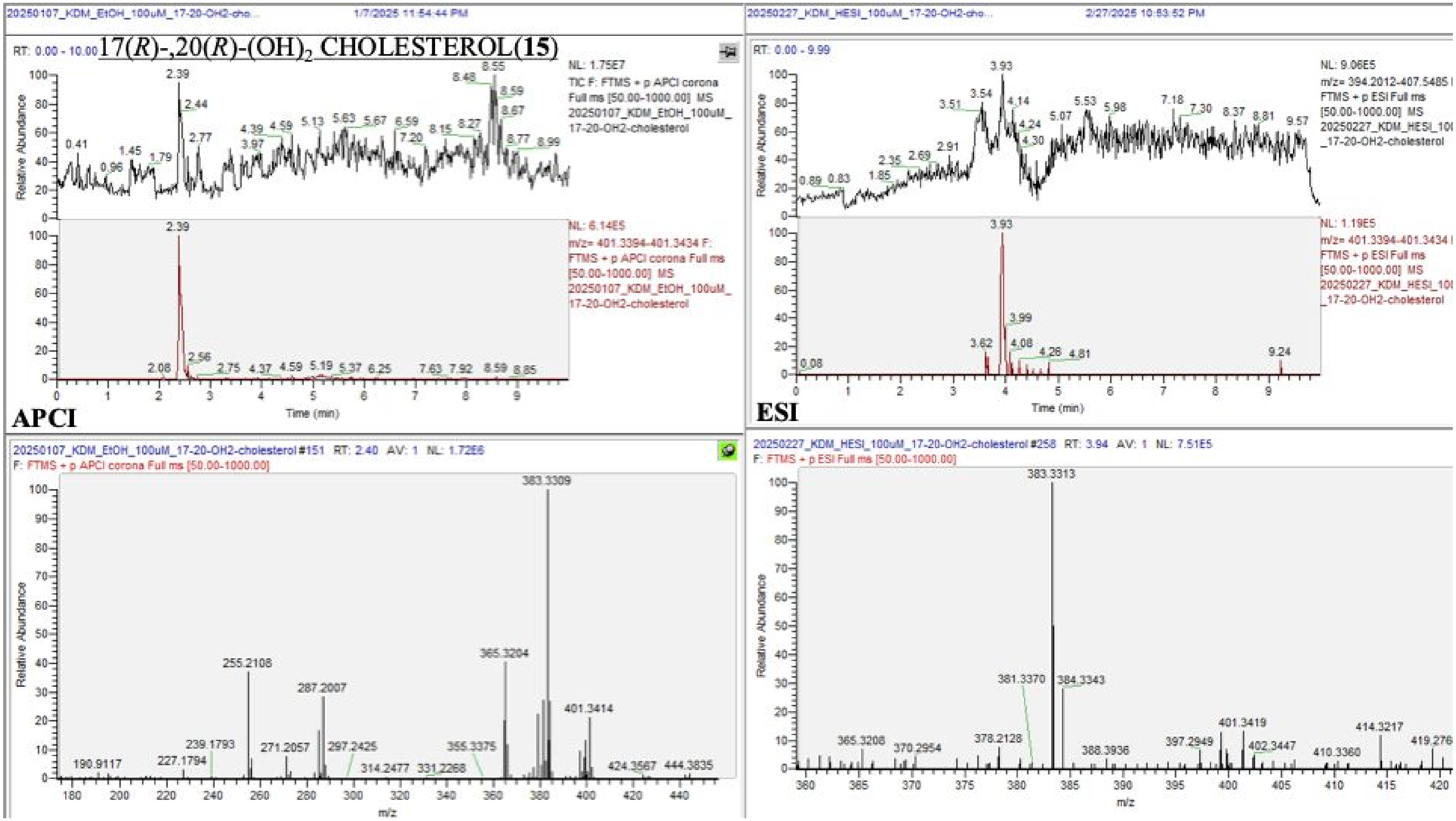

Figure S15. LC-HRMS chromatograms of 17*R*,20*R*-$(OH)_2$ cholesterol (**15**, 100 μM) ionized by APCI (left) and HESI (right).

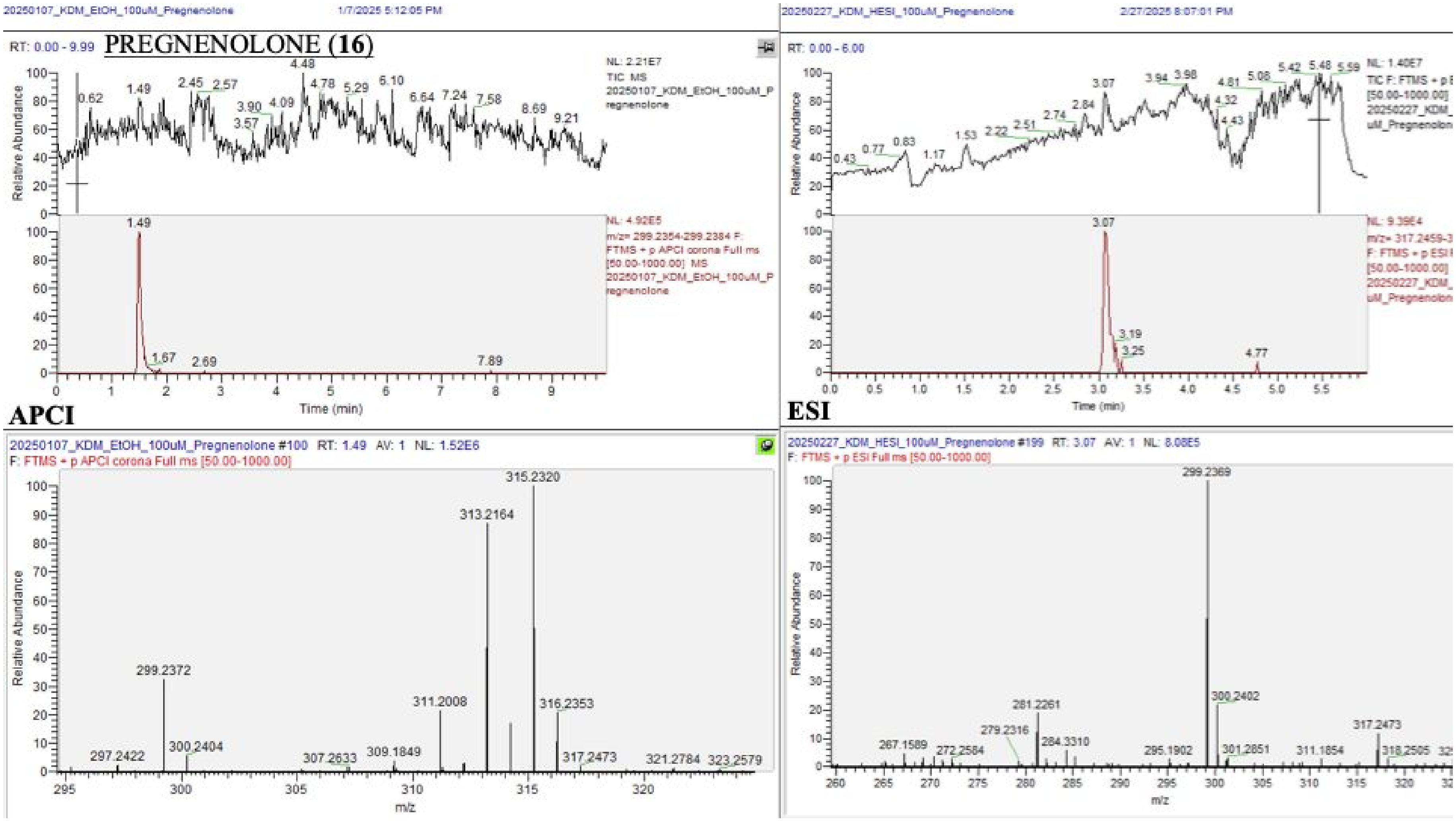

Figure S16. LC-HRMS chromatograms of pregnenolone (**16**, 100 µM) ionized by APCI (left) and HESI (right).

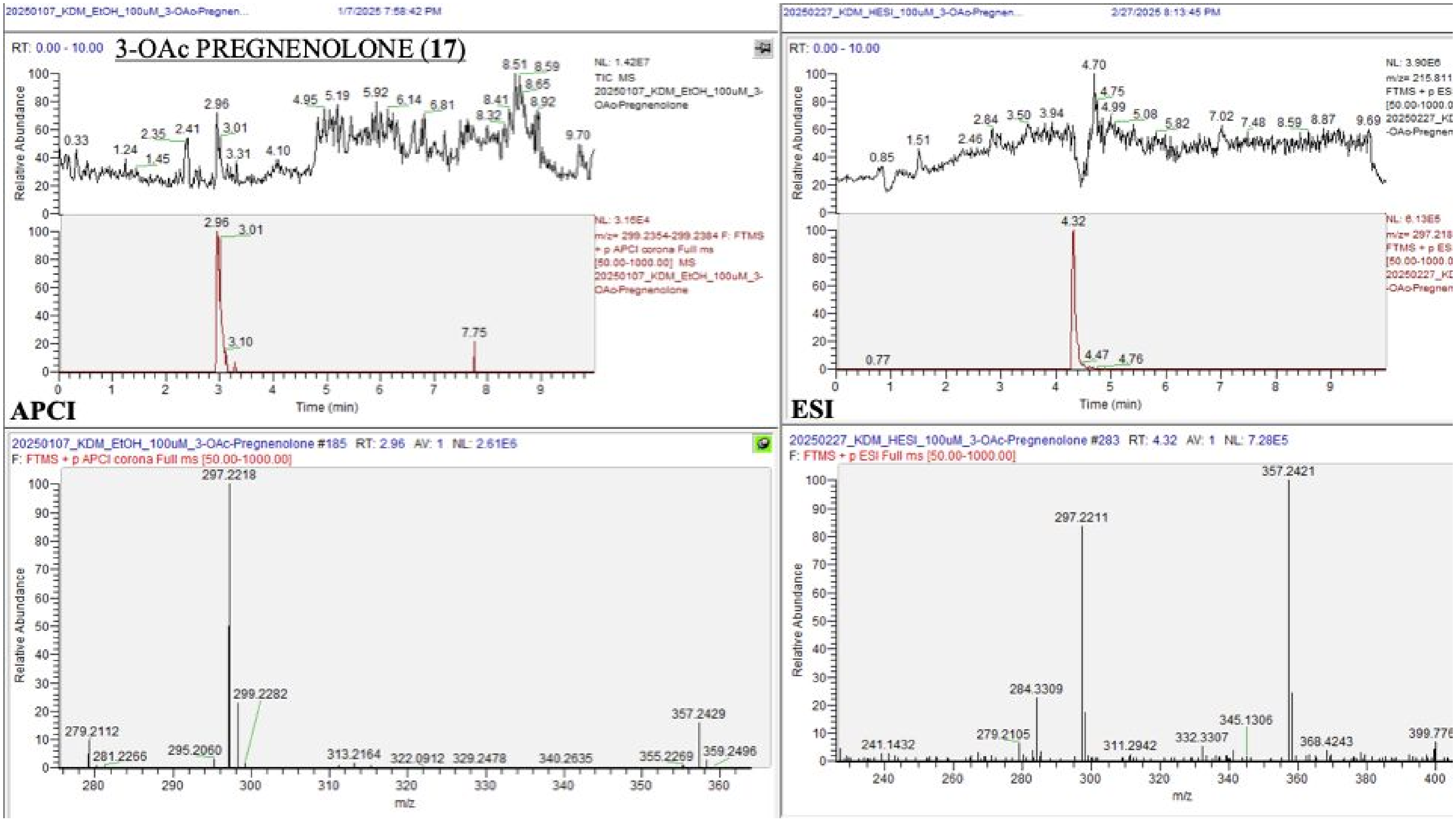

Figure S17. LC-HRMS chromatograms of 3-OAc pregnenolone (**17**, 100 μM) ionized by APCI (left) and HESI (right).

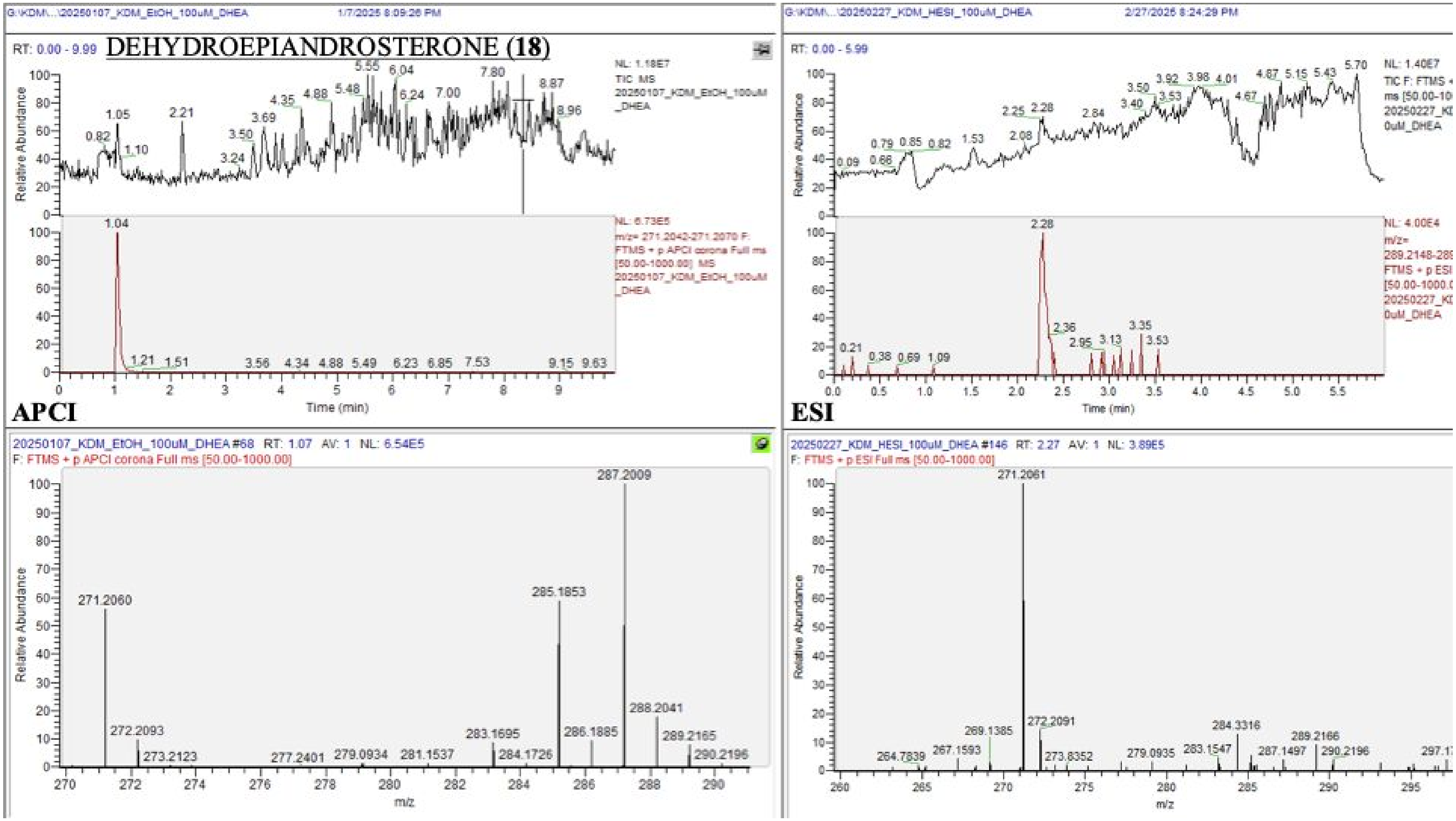

Figure S18. LC-HRMS chromatograms of dehydroepiandrosterone (**18**, 100 μM) ionized by APCI (left) and HESI (right).

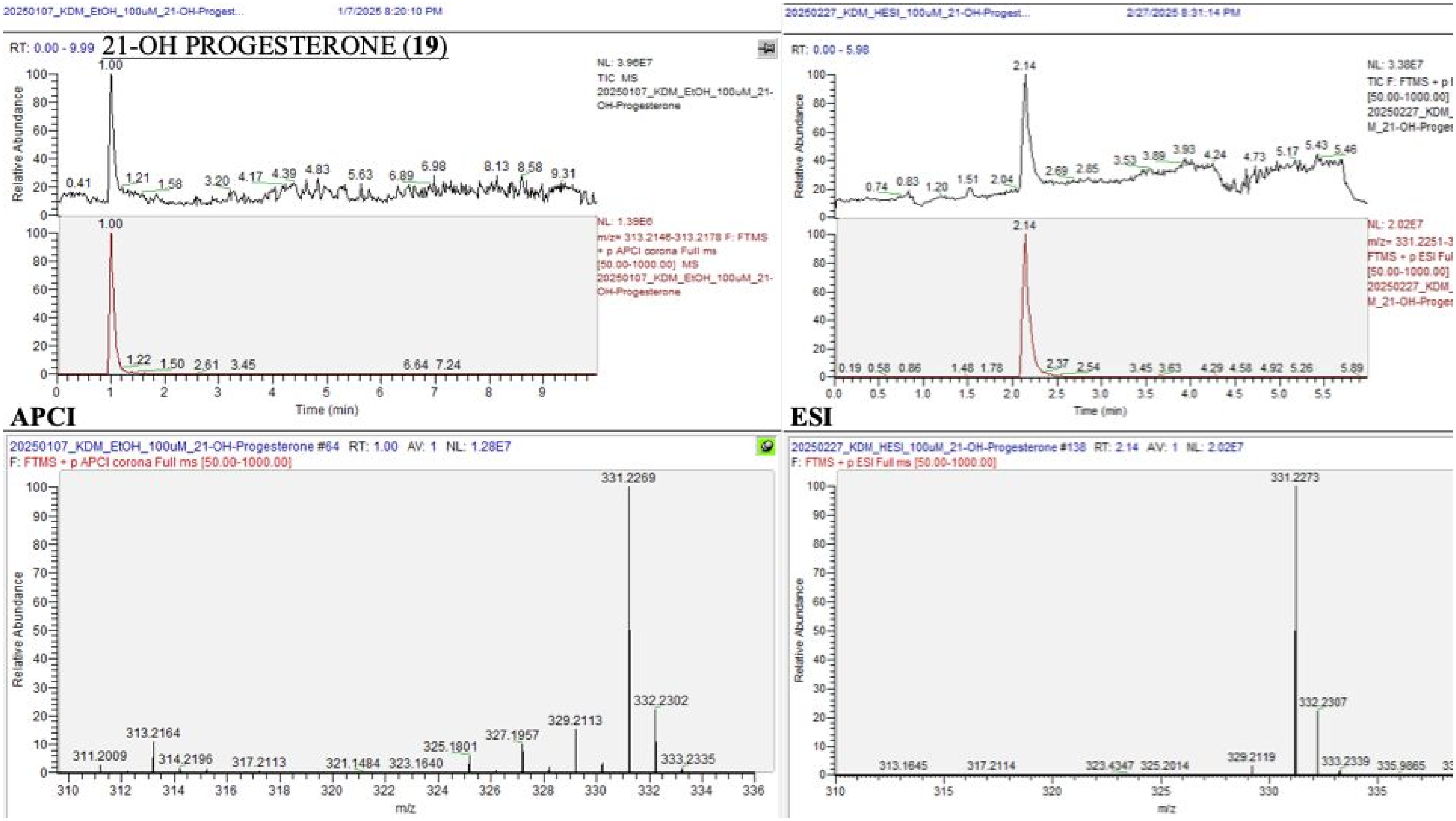

Figure S19. LC-HRMS chromatograms of 21-OH progesterone (**19**, 100 μM) ionized by APCI (left) and HESI (right).

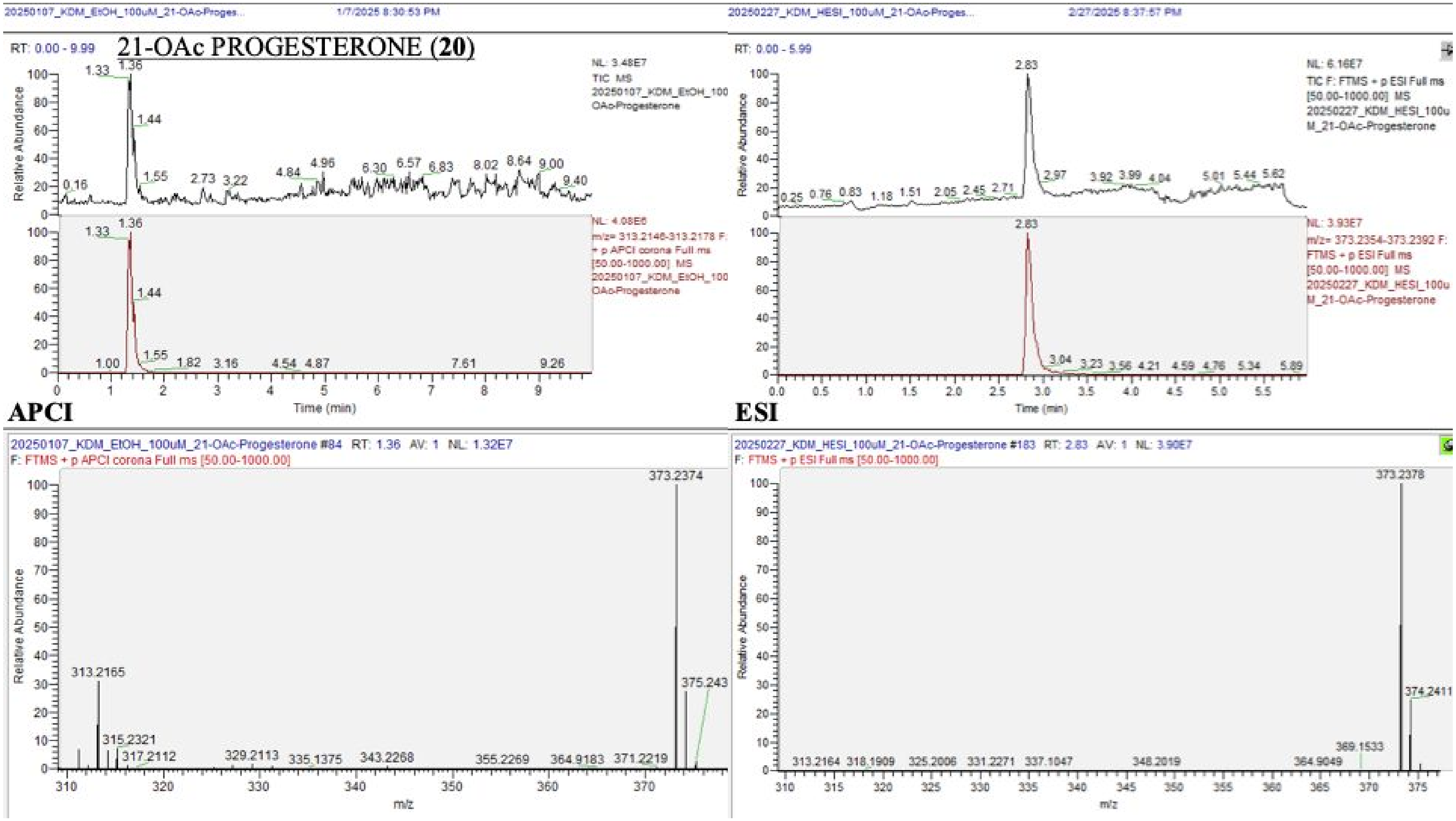

Figure S20. LC-HRMS chromatograms of 21-OAc progesterone (**20**, 100 μM) ionized by APCI (left) and HESI (right).

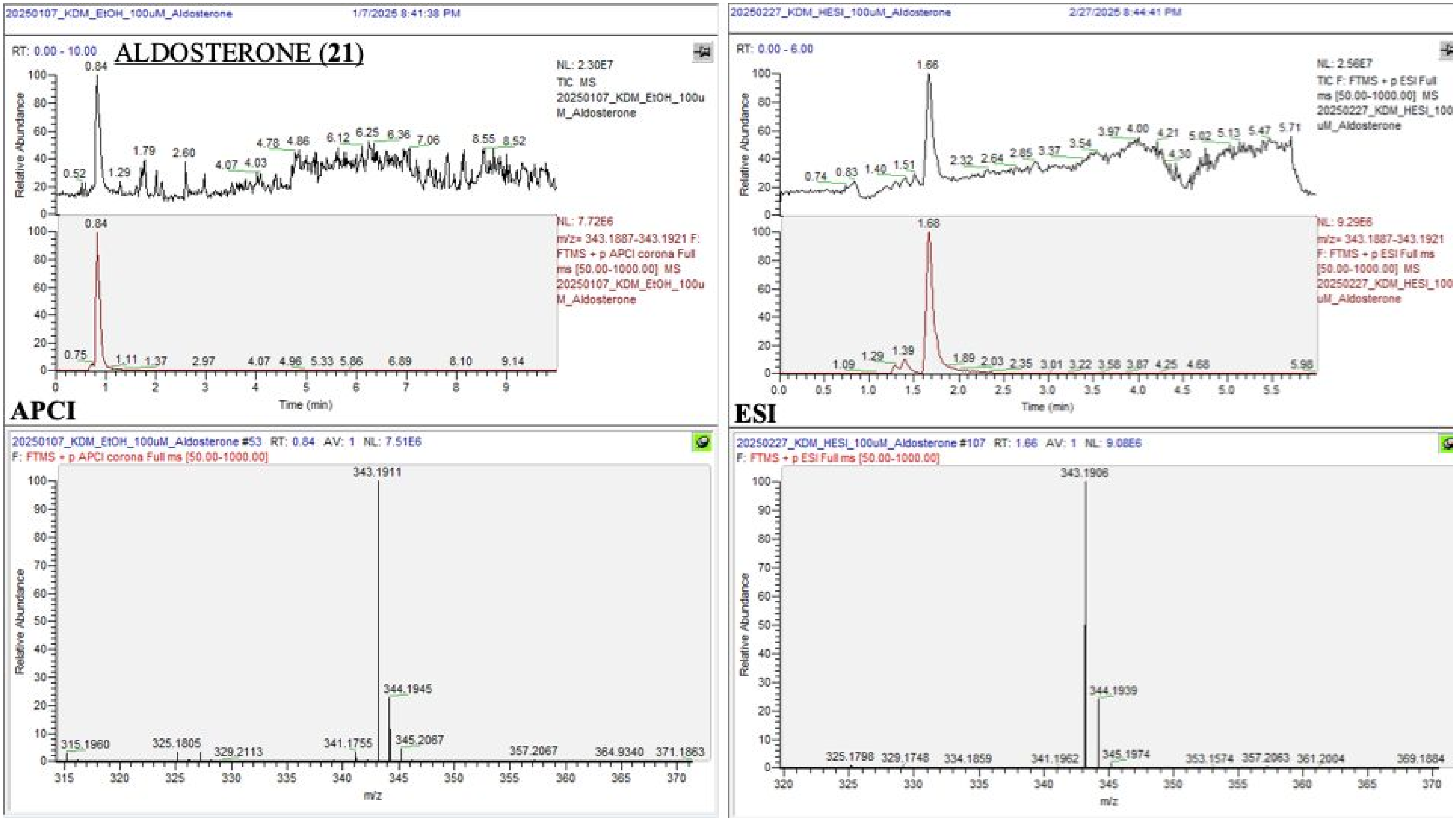

Figure S21. LC-HRMS chromatograms of aldosterone (**21**, 100 μM) ionized by APCI (left) and HESI (right).

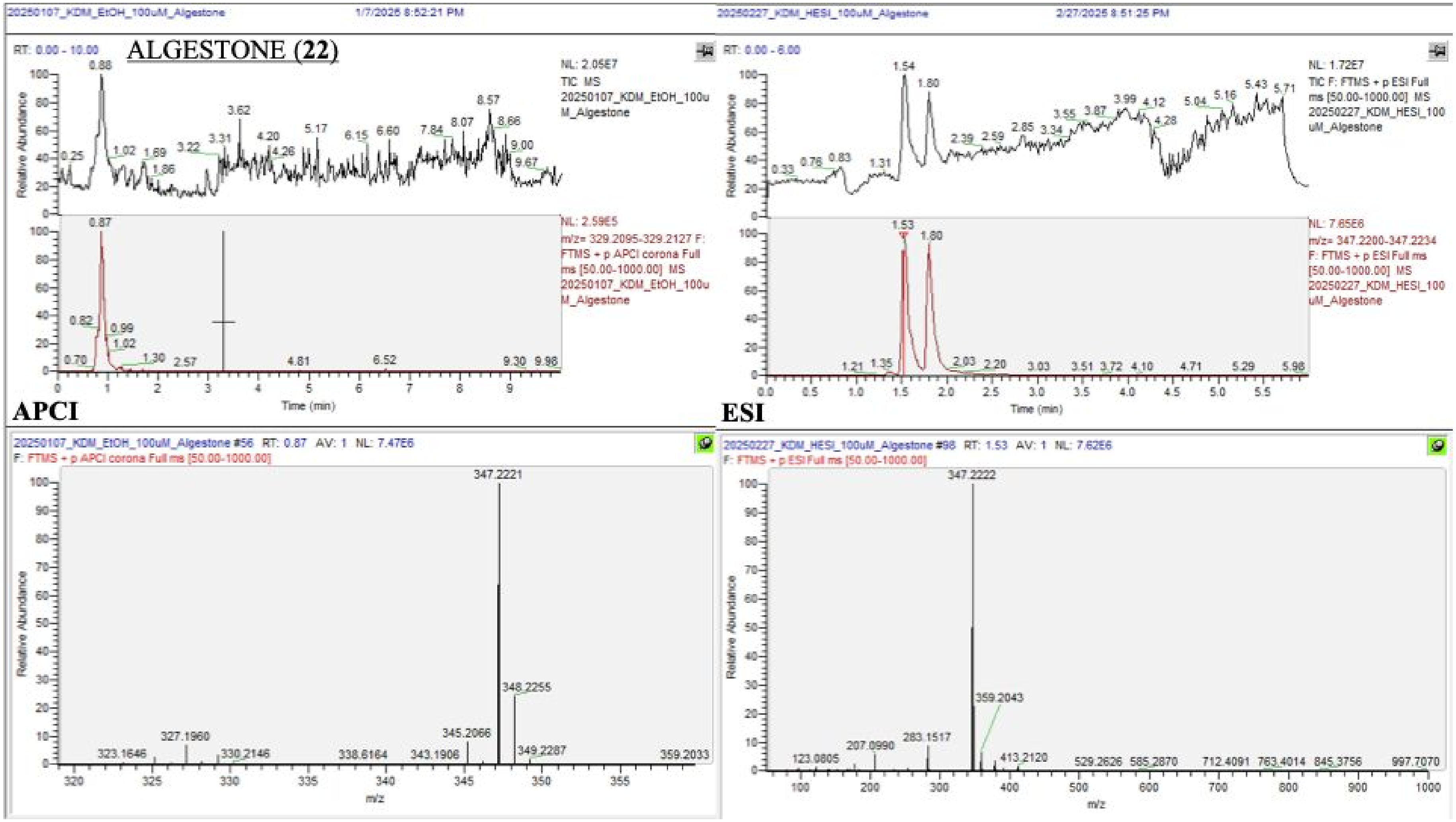

Figure S22. LC-HRMS chromatograms of algestone (**22**, 100 μM) ionized by APCI (left) and HESI (right).

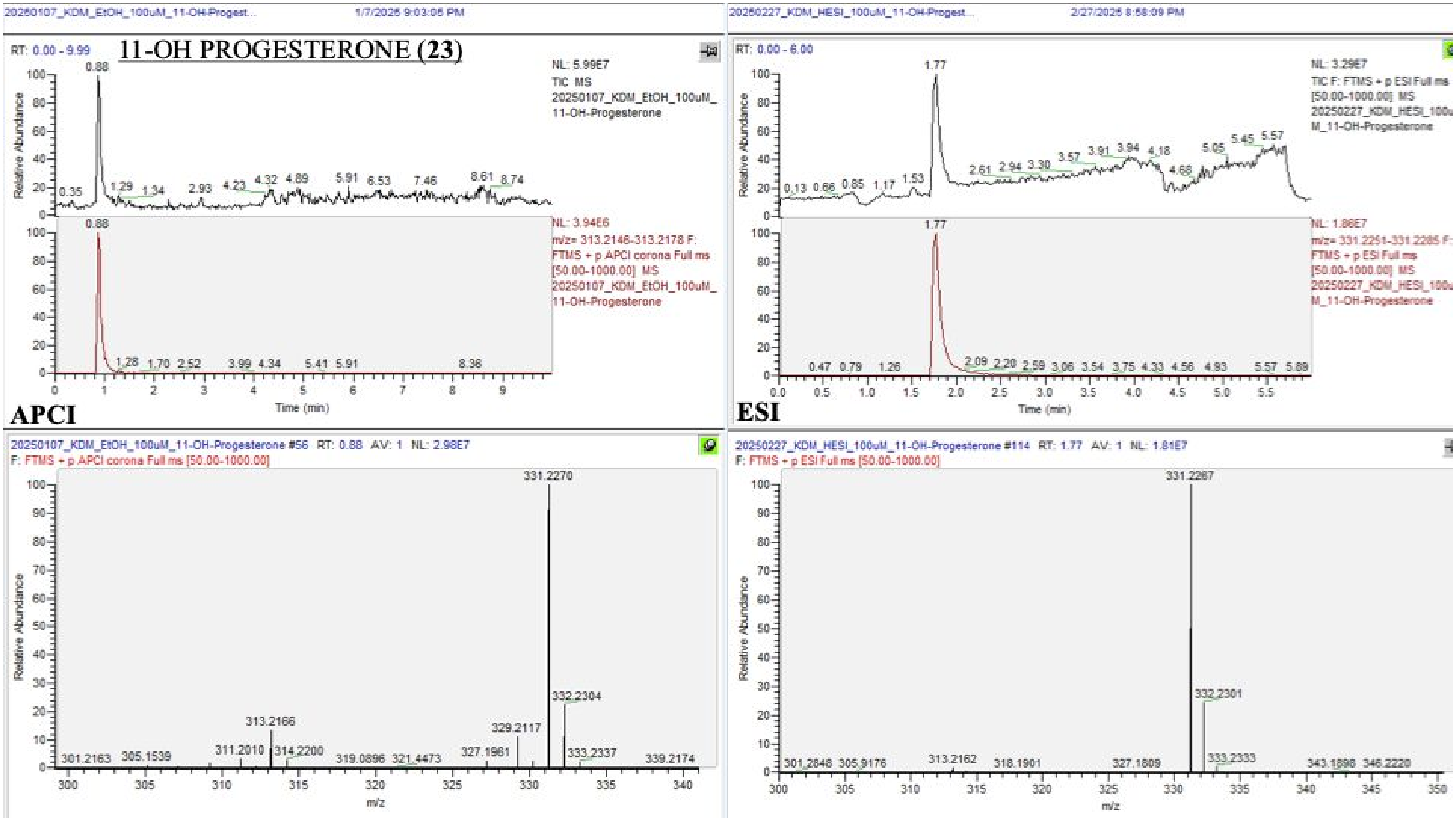

Figure S23. LC-HRMS chromatograms of 11-OH progesterone (**23**, 100 μM) ionized by APCI (left) and HESI (right).

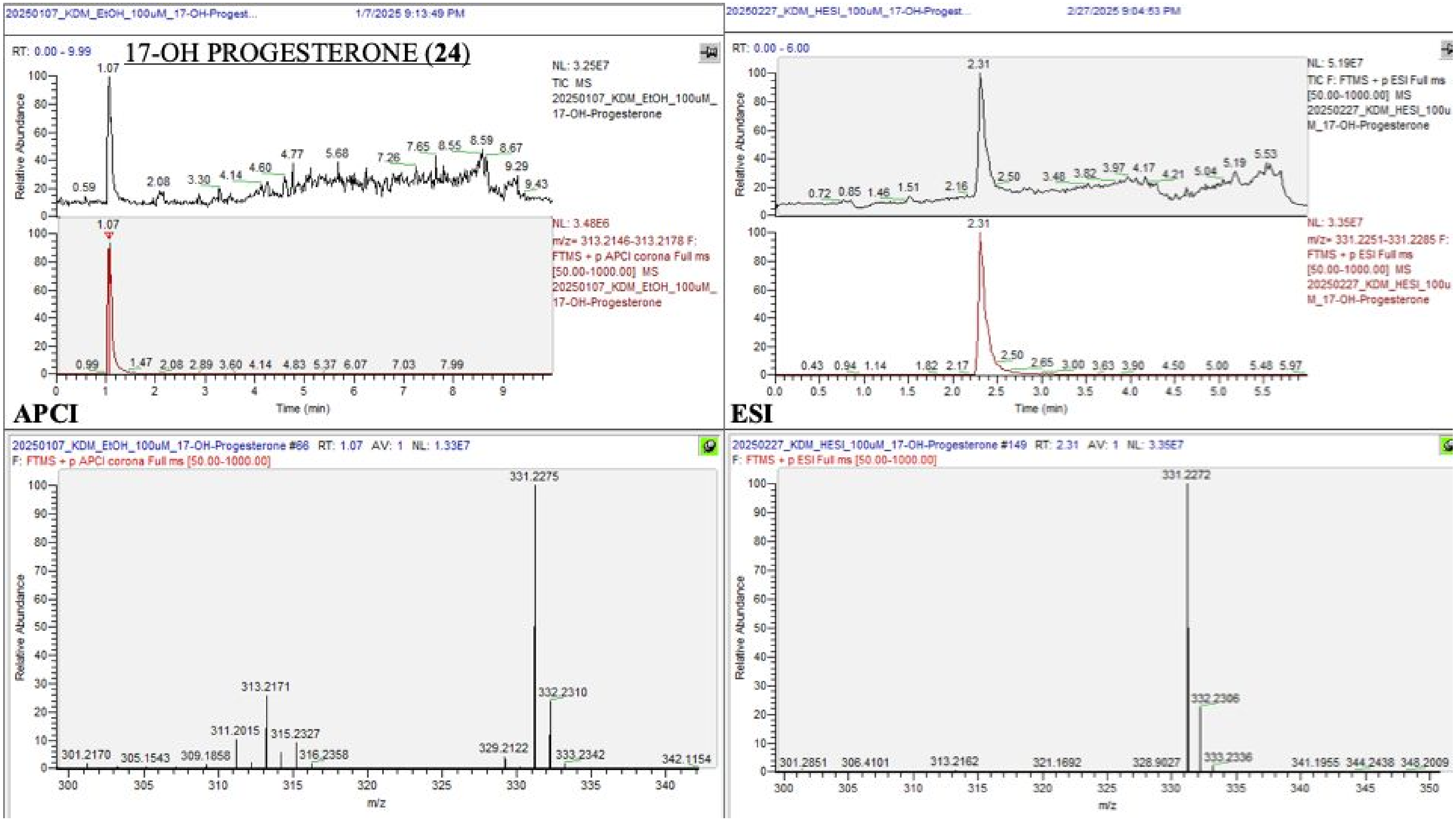

Figure S24. LC-HRMS chromatograms of 17-OH progesterone (**24**, 100 µM) ionized by APCI (left) and HESI (right).

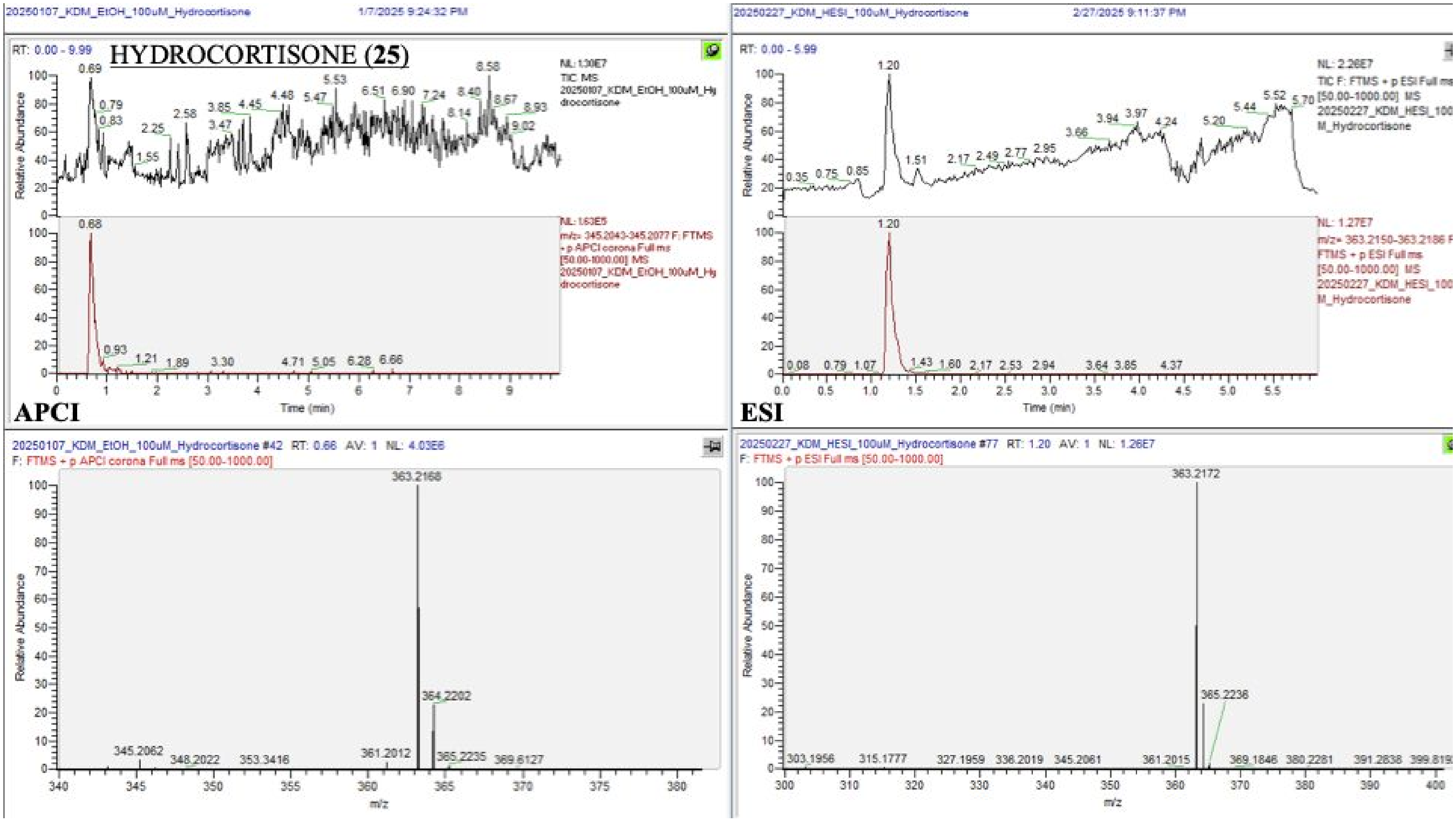

Figure S25. LC-HRMS chromatograms of hydrocortisone (**25**, 100 µM) ionized by APCI (left) and HESI (right).

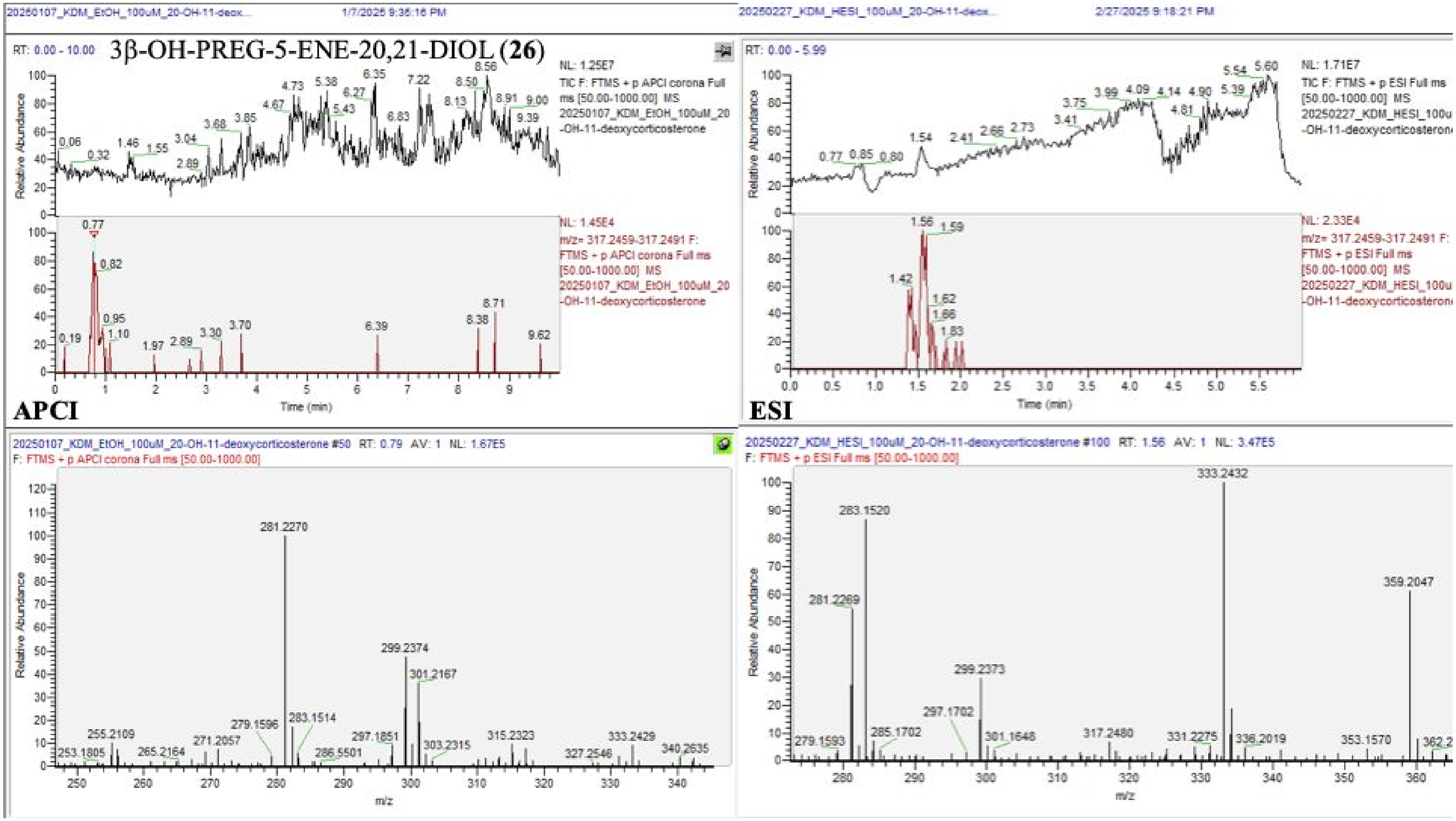

Figure S26. LC-HRMS chromatograms of 3β-OH-preg-5-ene-20,21-diol (**26**, 100 μM) ionized by APCI (left) and HESI (right). *Original name of 20-OH-11-deoxycorticosterone (printed on the spectrum) has been corrected to current name.*

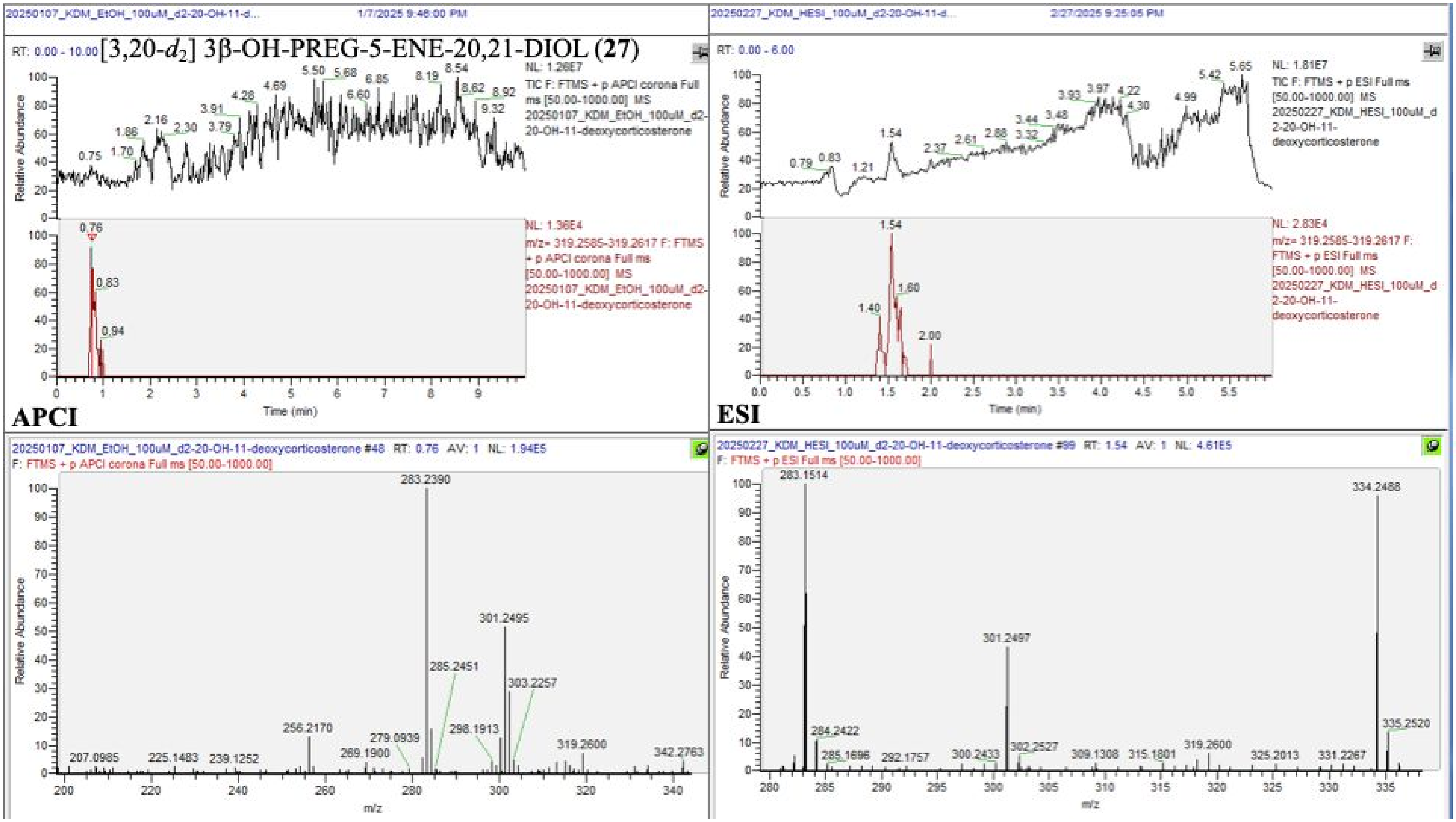

Figure S27. LC-HRMS chromatograms of [3,20-d_2] 3β-OH-preg-5-ene-20,21-diol (**27**, 100 μM) ionized by APCI (left) and HESI (right). *Original name of d_2-20-OH-11-deoxycorticosterone (printed on the spectrum) has been corrected to current name.*

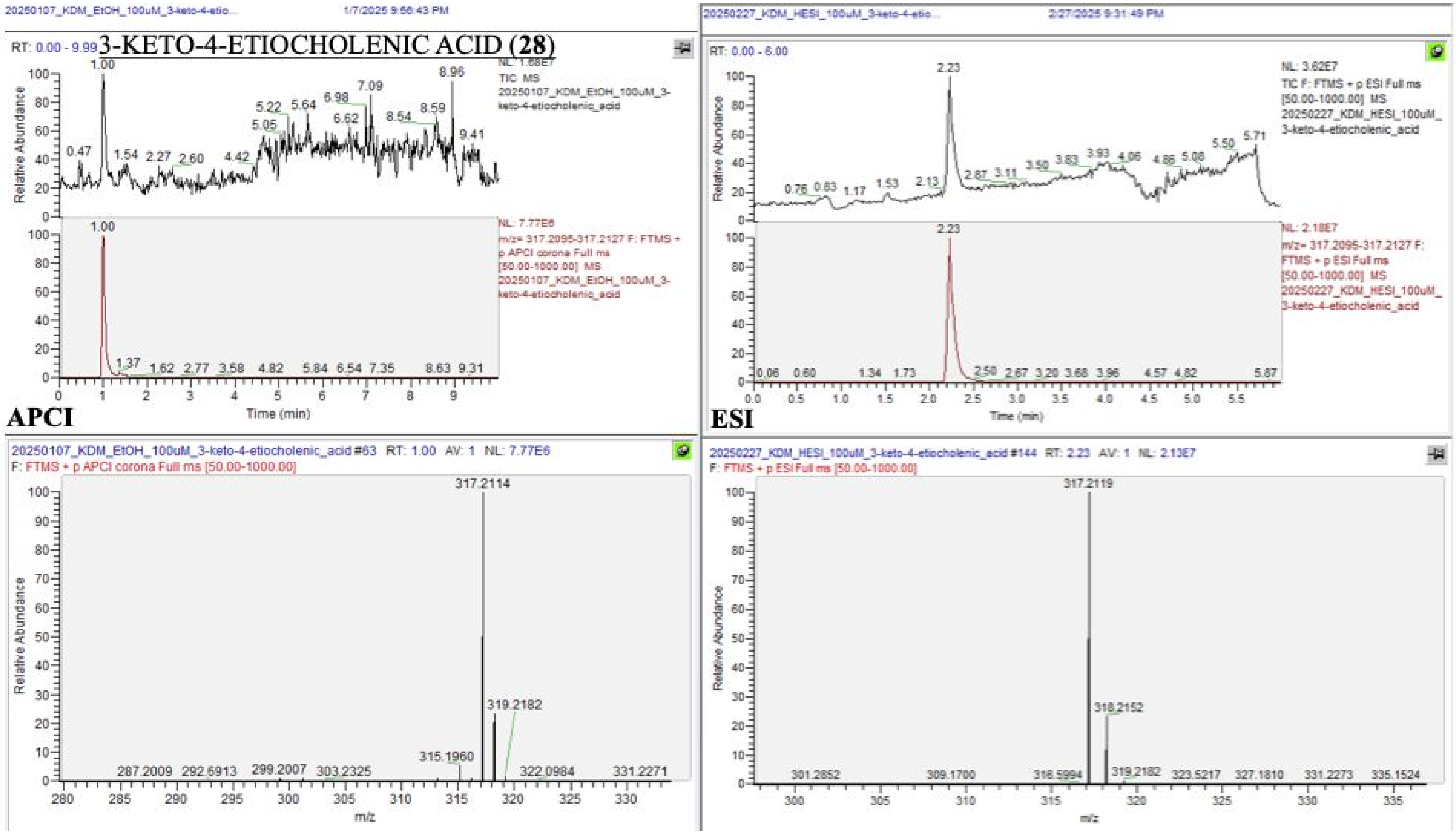

Figure S28. LC-HRMS chromatograms of 3-keto-4-etiocholenic acid (**28**, 100 μM) ionized by APCI (left) and HESI (right).

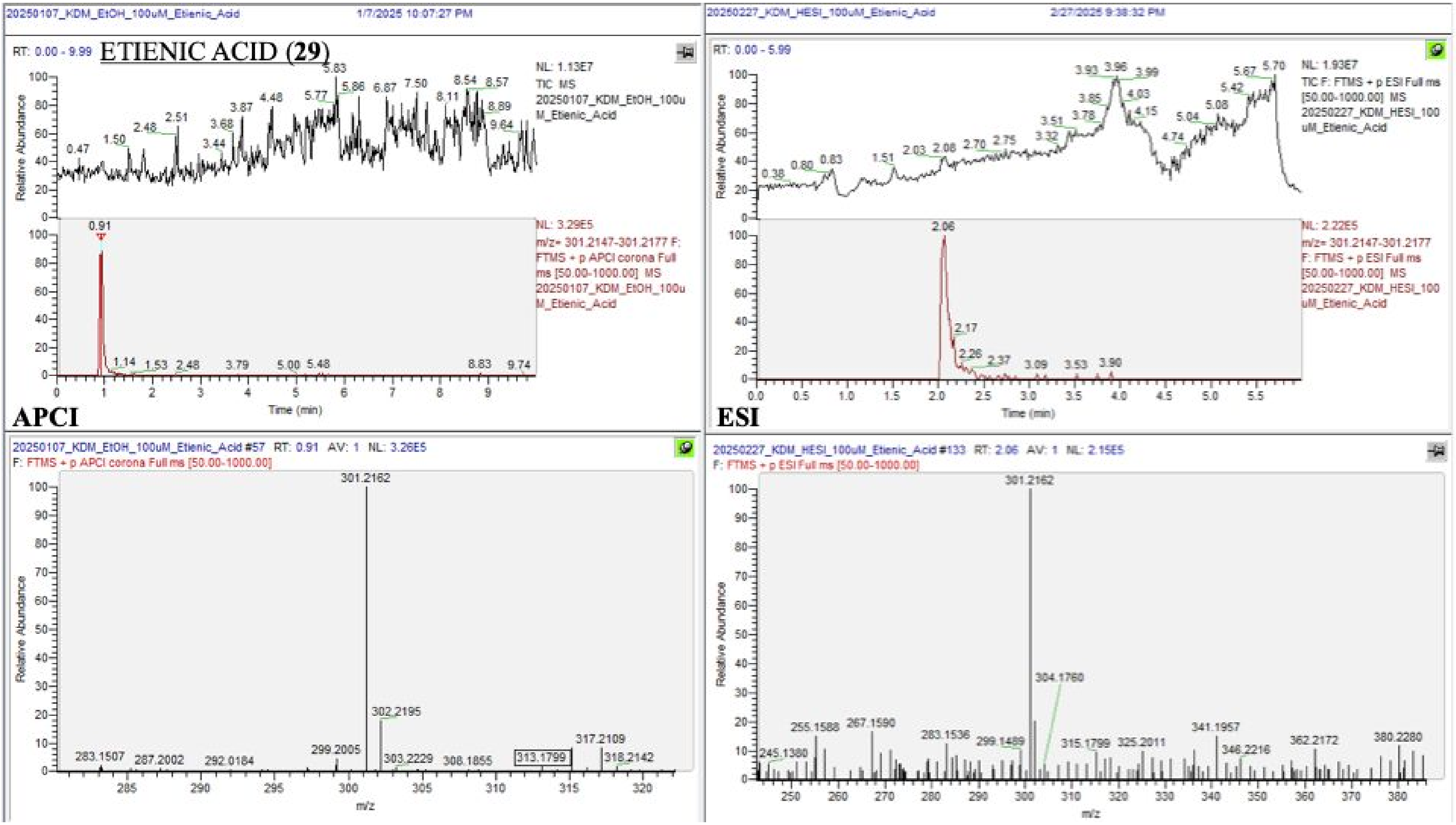

Figure S29. LC-HRMS chromatograms of etienic acid (**29**, 100 μM) ionized by APCI (left) and HESI (right).

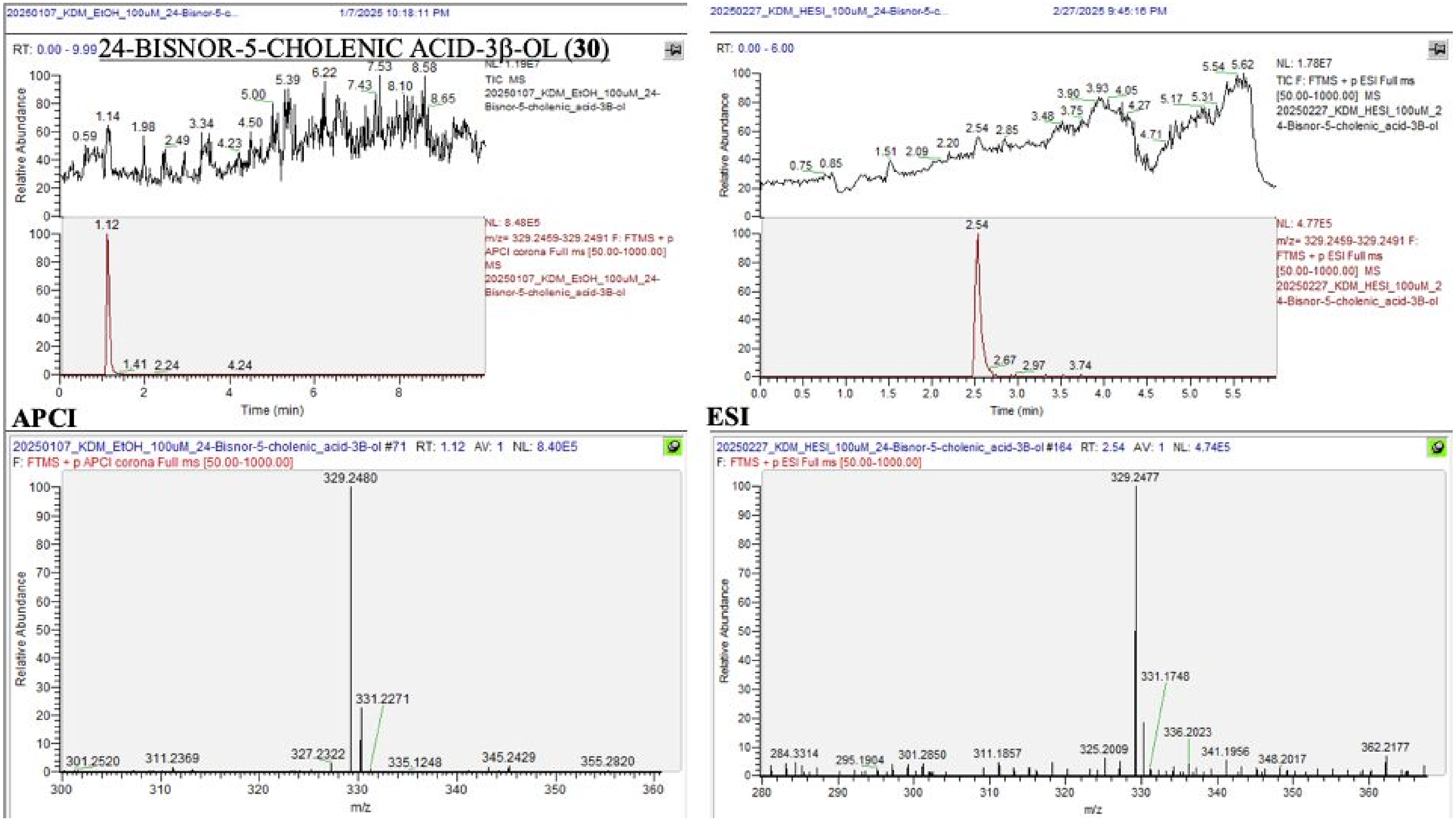

Figure S30. LC-HRMS chromatograms of 24-bisnor-5-cholenic acid-3β-ol (**30**, 100 μM) ionized by APCI (left) and HESI (right).

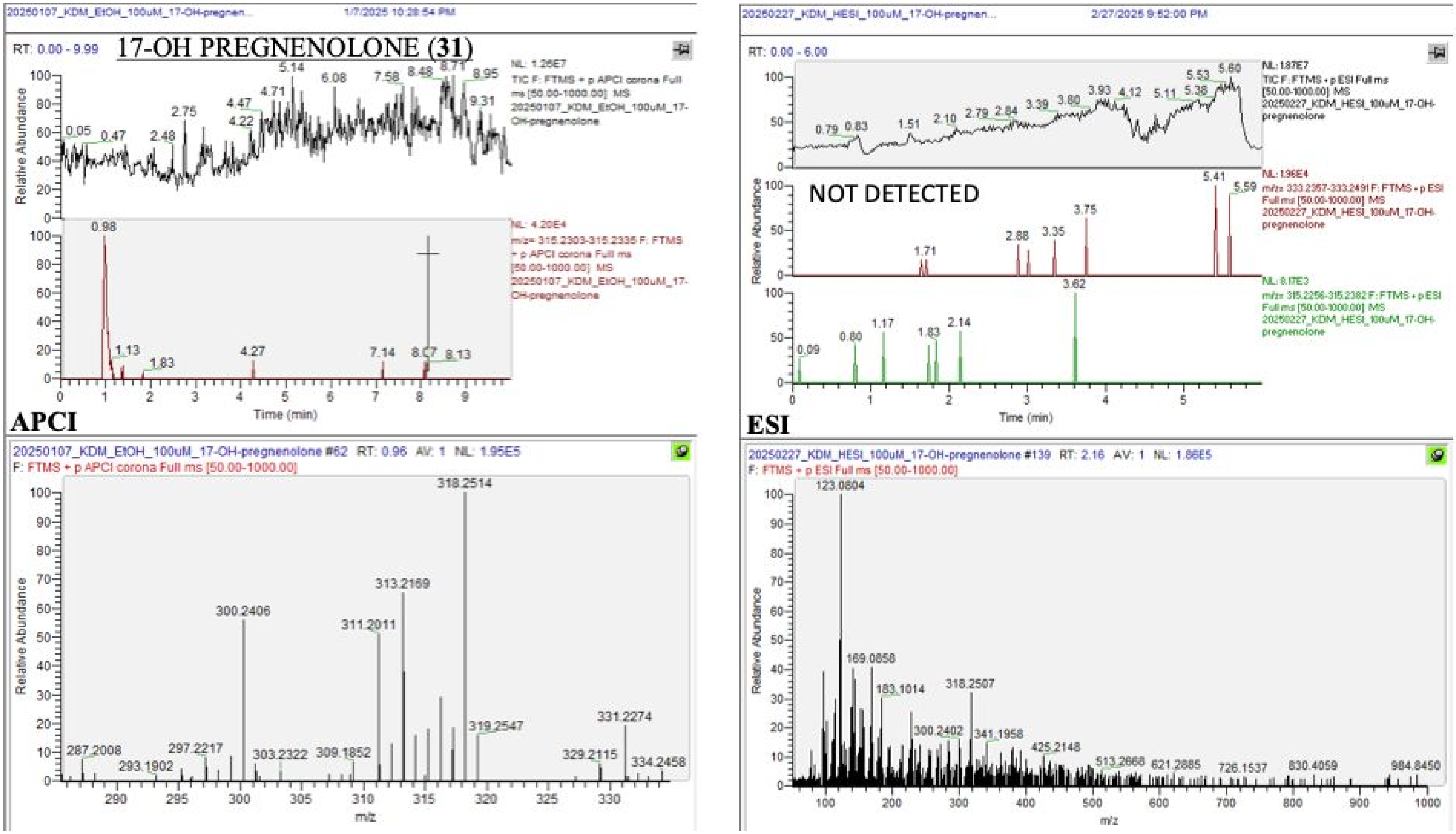

Figure S31. LC-HRMS chromatograms of 17-OH pregnenolone (**31**, 100 μM) ionized by APCI (left) and HESI (right).

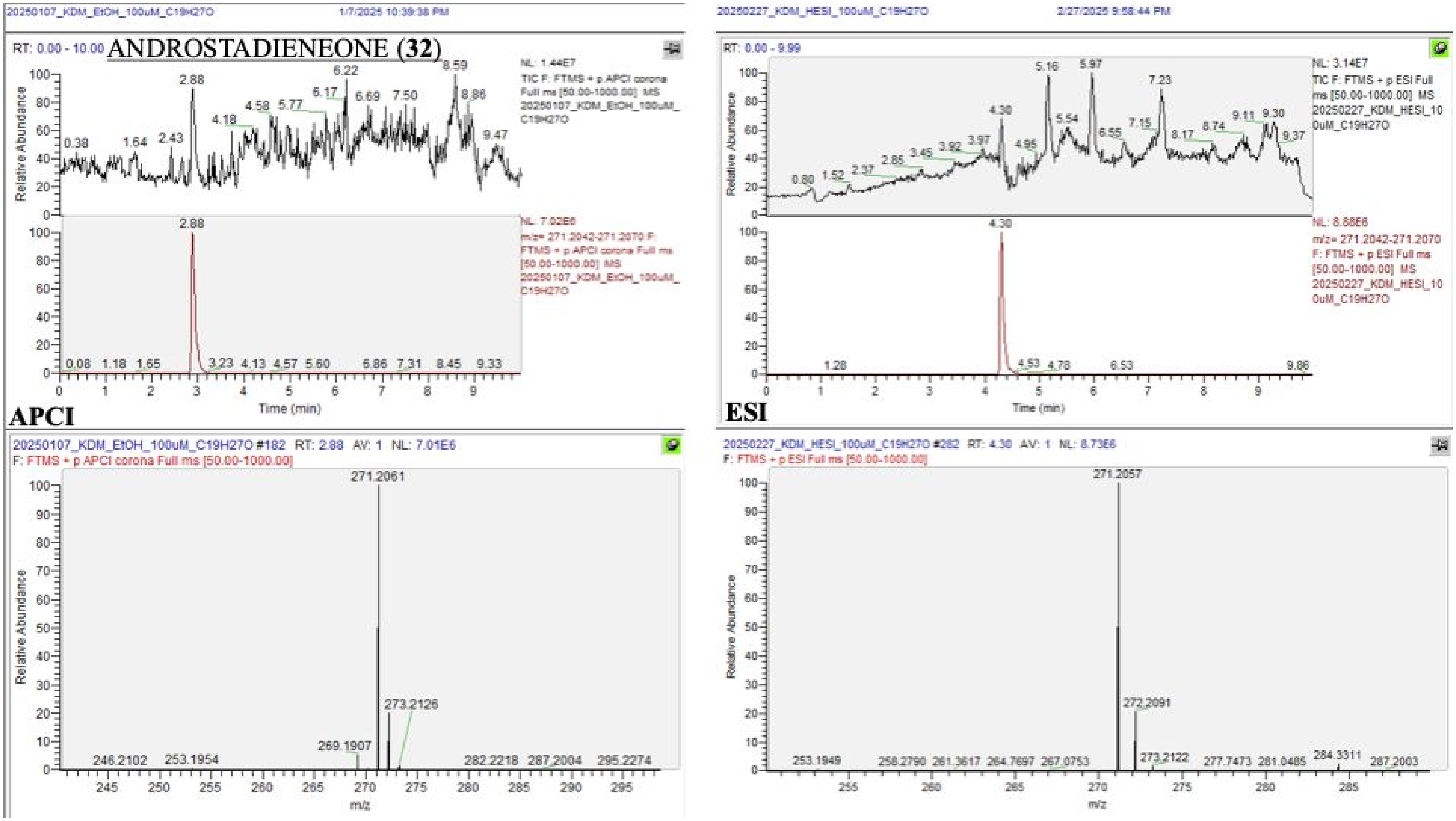

Figure S32. LC-HRMS chromatograms of androstadieneone (**32**, 100 µM) ionized by APCI (left) and HESI (right).

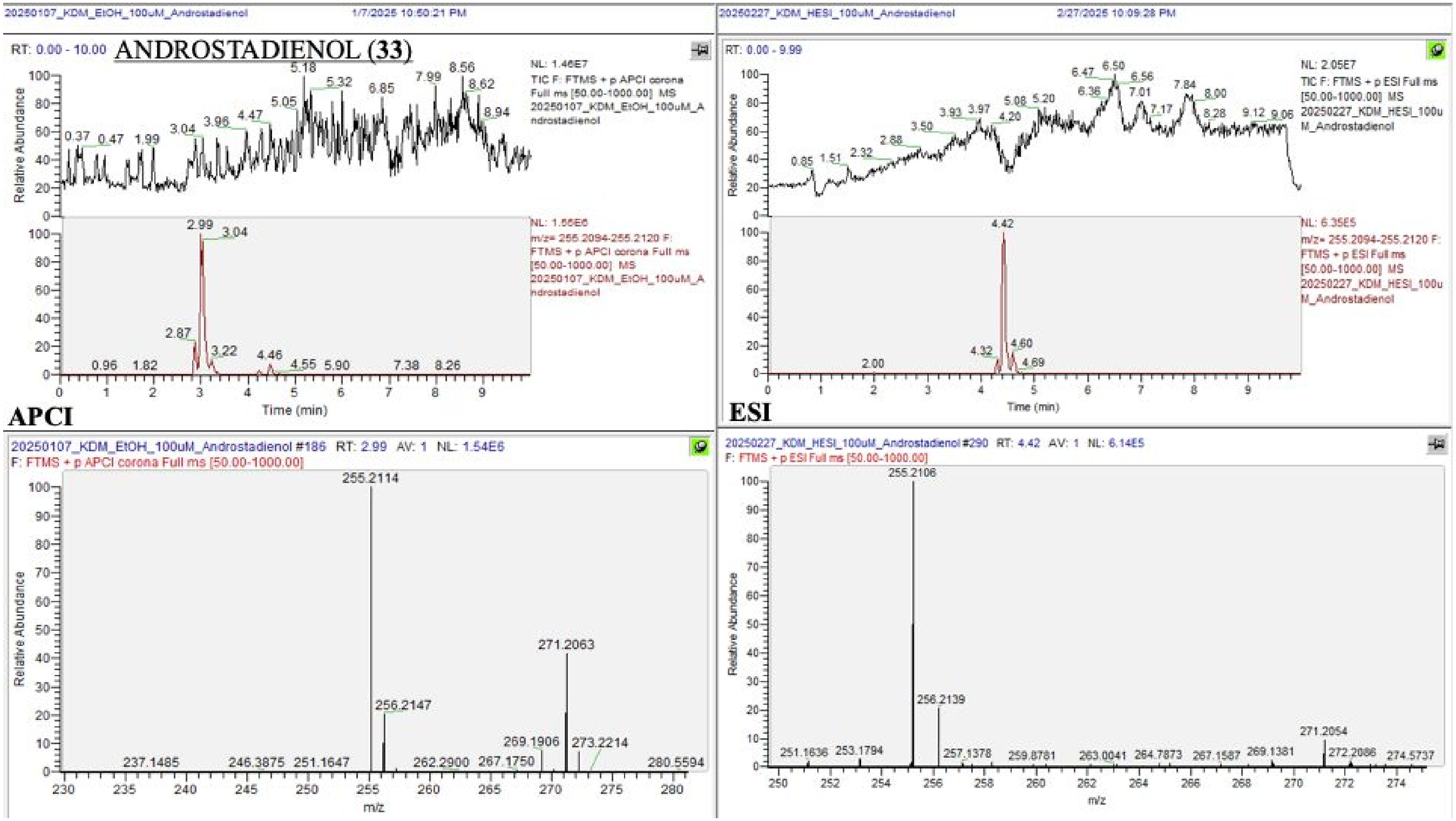

Figure S33. LC-HRMS chromatograms of androstadienol (**33**, 100 μM) ionized by APCI (left) and HESI (right).

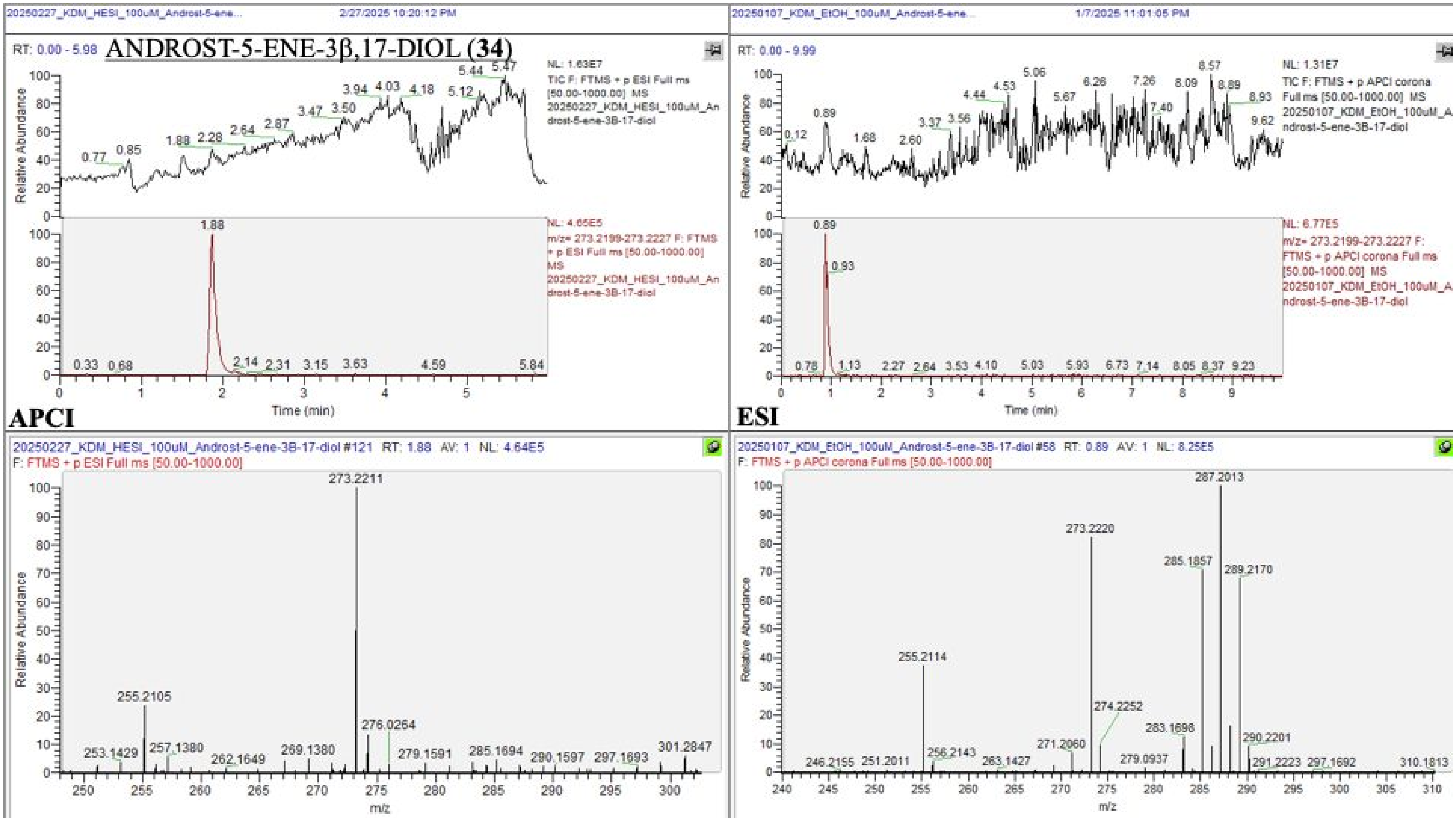

Figure S34. LC-HRMS chromatograms of androst-5-ene-3β,17-diol (**34**, 100 μM) ionized by APCI (left) and HESI (right).

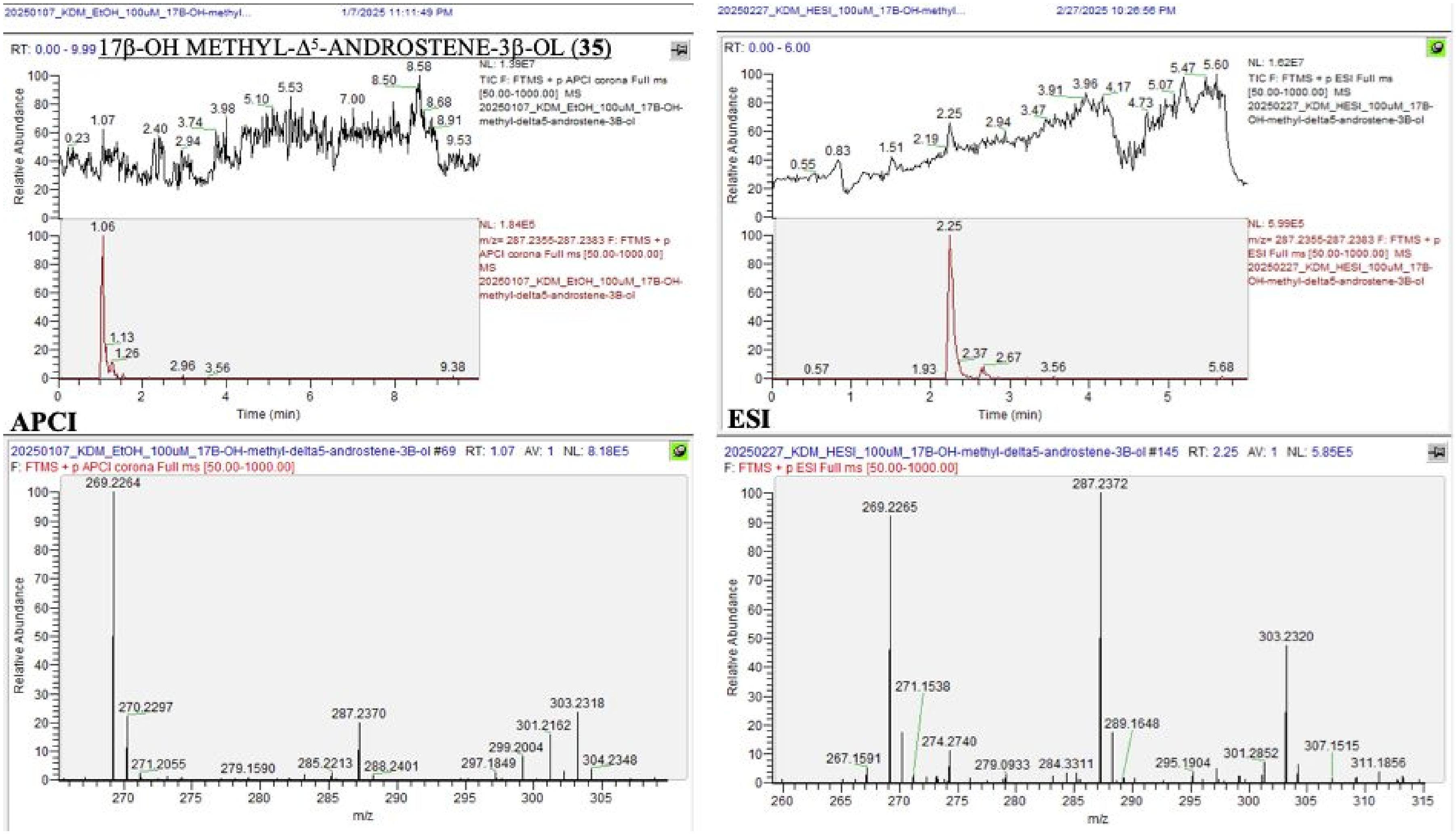

Figure S35. LC-HRMS chromatograms of 17β-OH methyl-Δ^5-androstene-3β-ol (**35**, 100 μM) ionized by APCI (left) and HESI (right).

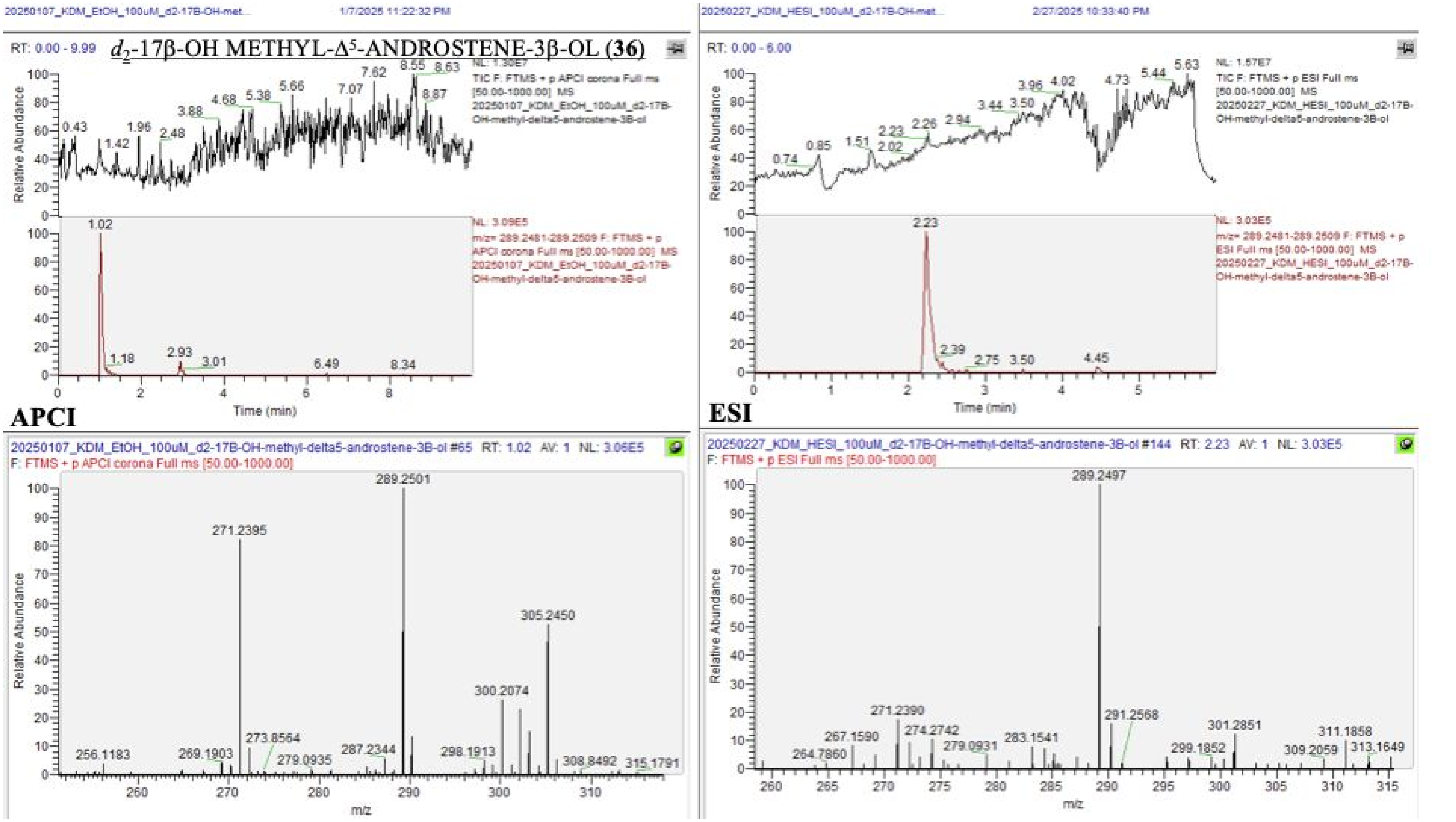

Figure S36. LC-HRMS chromatograms of [d_2]-17β-OH methyl-Δ^5-androstene-3β-ol (**36**, 100 μM) ionized by APCI (left) and HESI

(right).

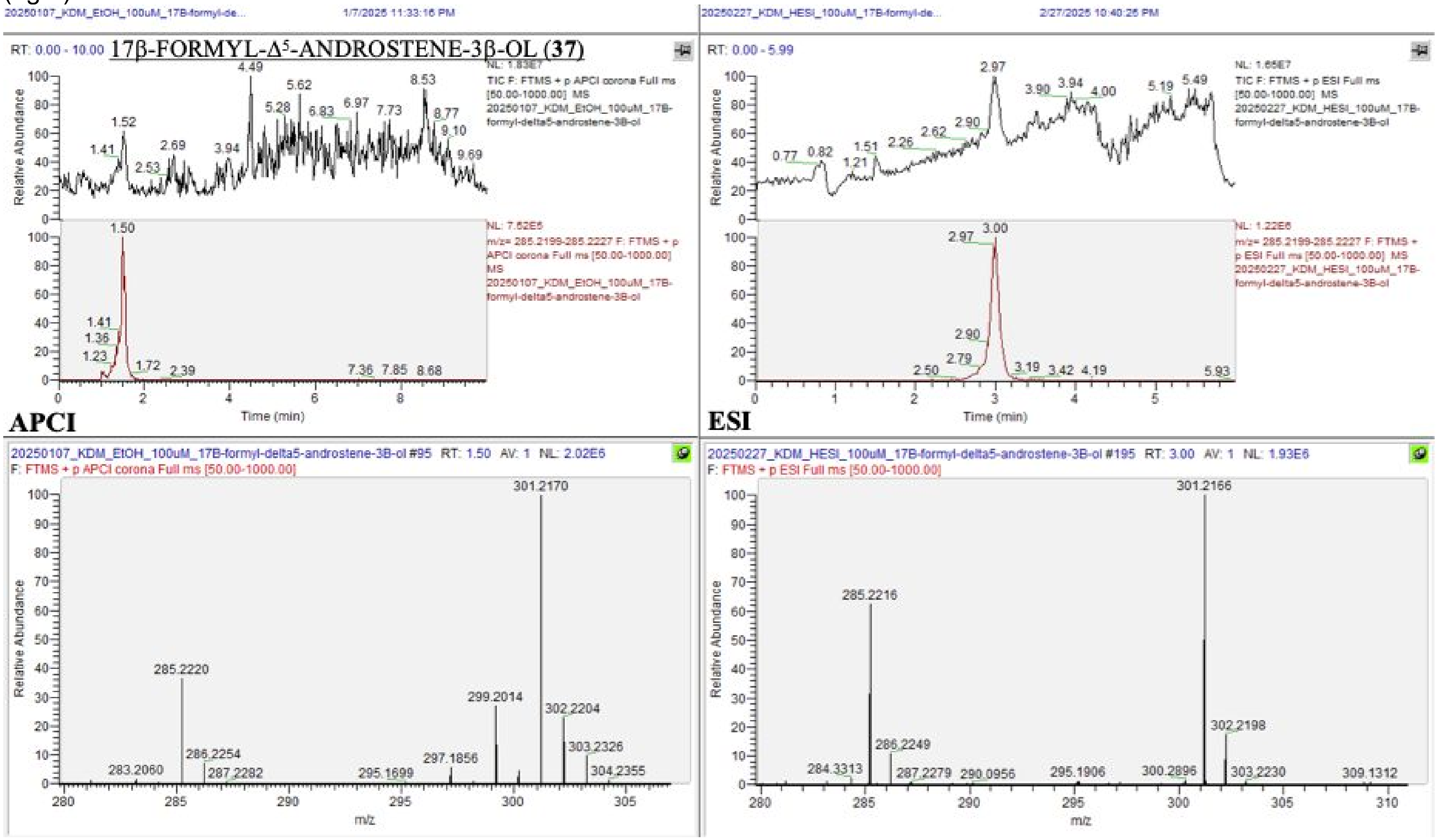

Figure S37. LC-HRMS chromatograms of 17β-formyl-Δ[5]-androstene-3β-ol (**37**, 100 μM) ionized by APCI (left) and HESI (right).

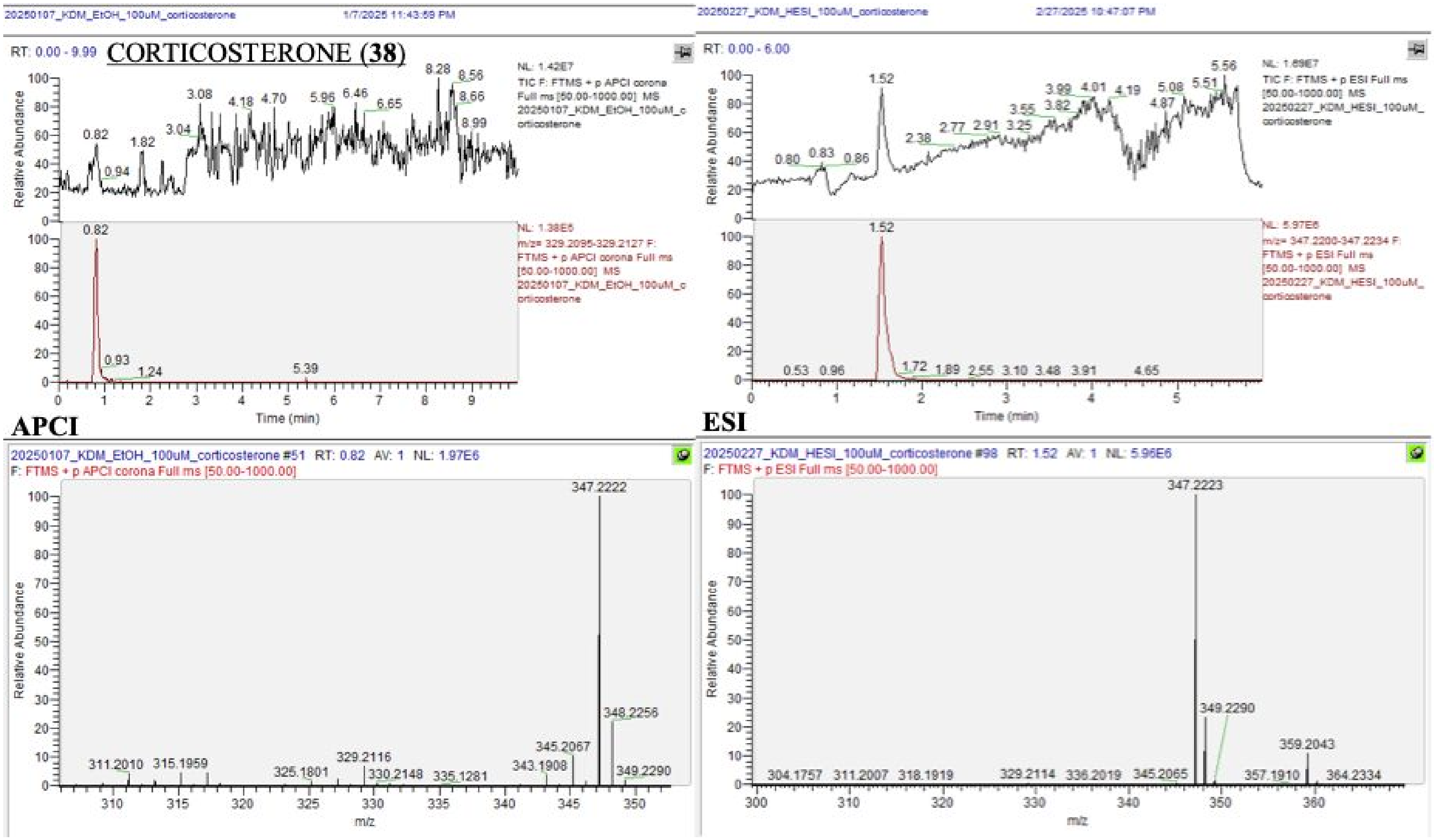

Figure S38. LC-HRMS chromatograms of corticosterone (**38**, 100 μM) ionized by APCI (left) and HESI (right).

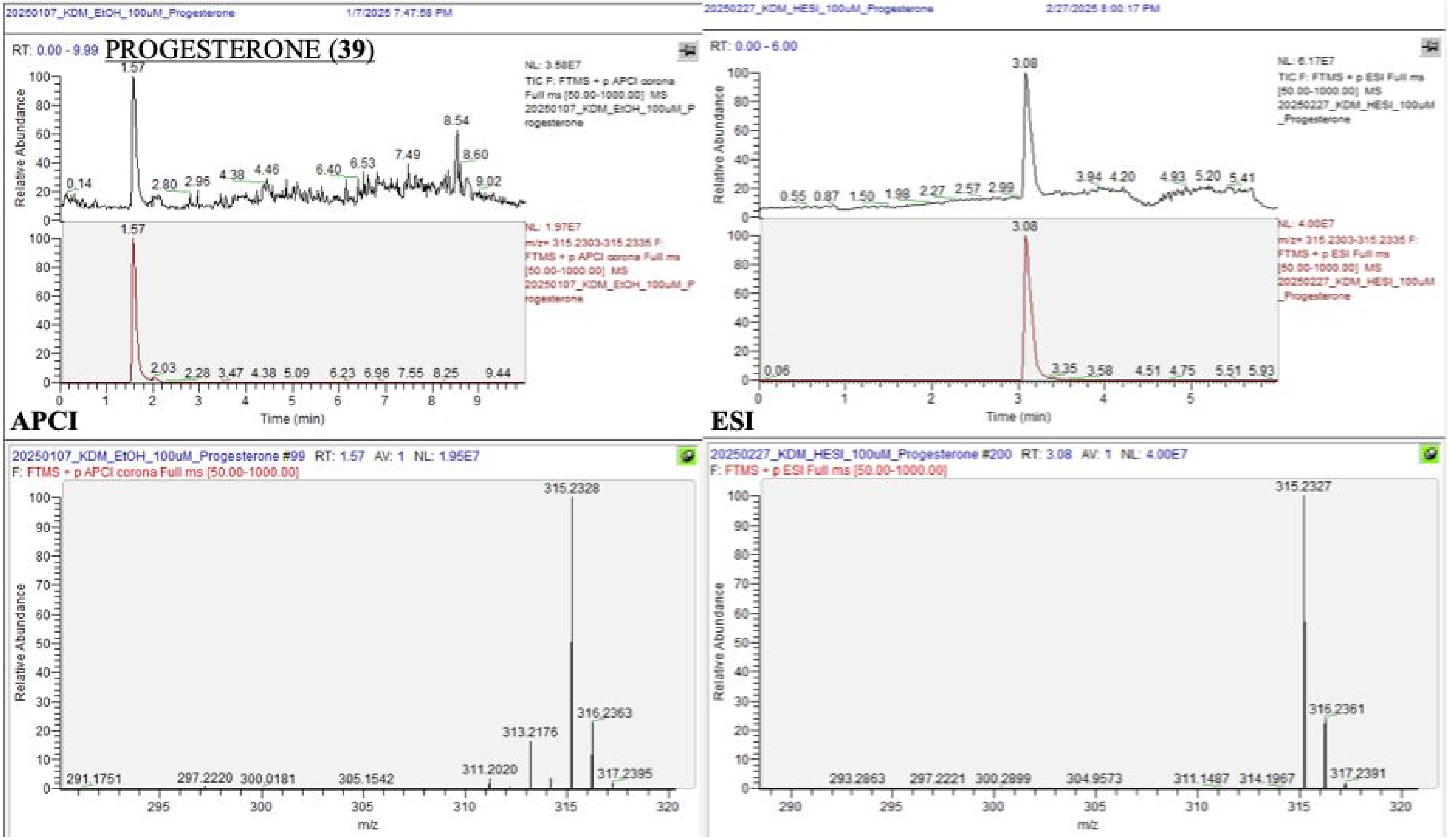

Figure S39. LC-HRMS chromatograms of progesterone (**39**, 100 μM) ionized by APCI (left) and HESI (right).

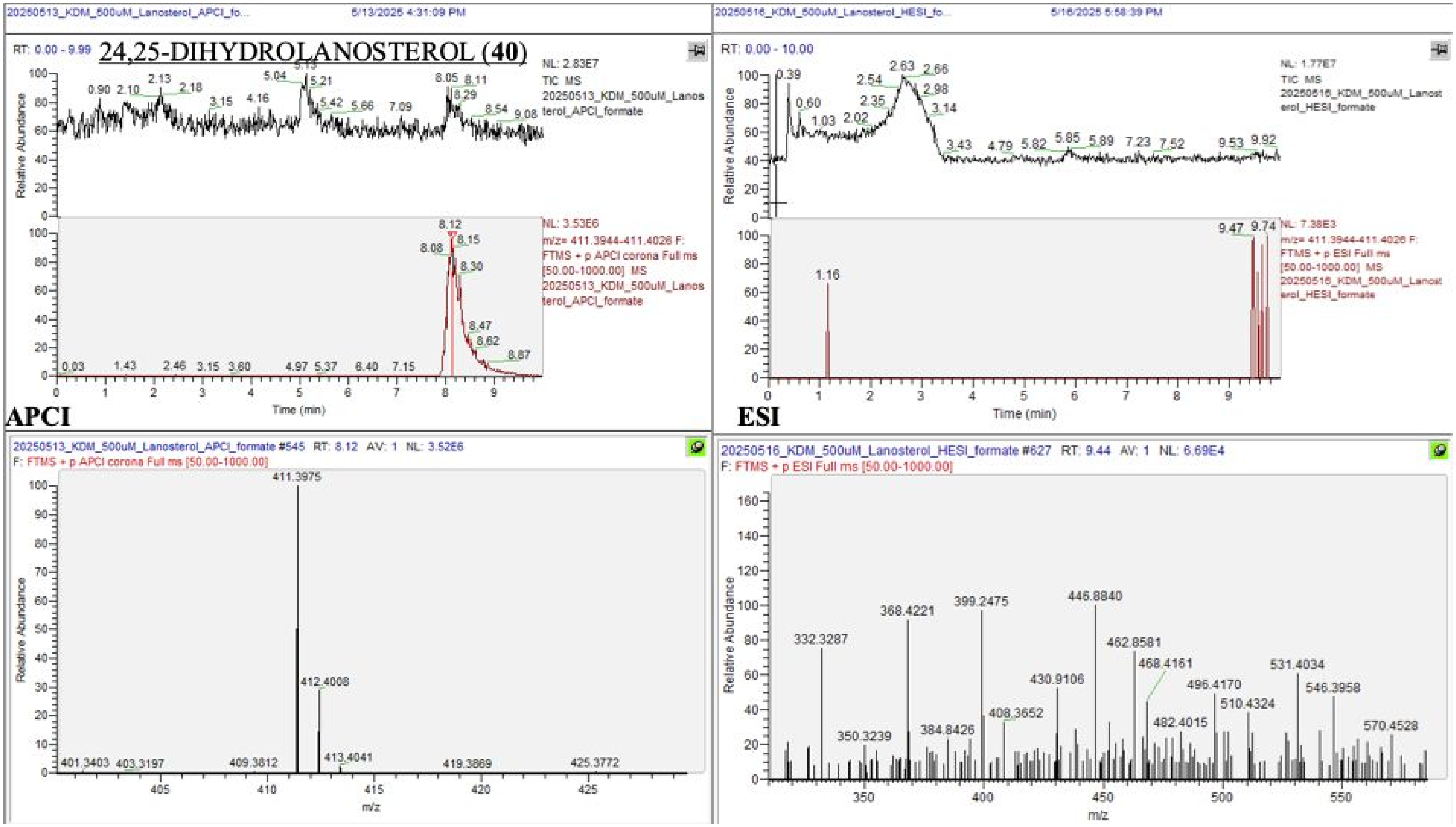

Figure S40. LC-HRMS chromatograms of 24,25-dihydrolanosterol (**40**, 500 μM) ionized by APCI (left) and HESI (right).

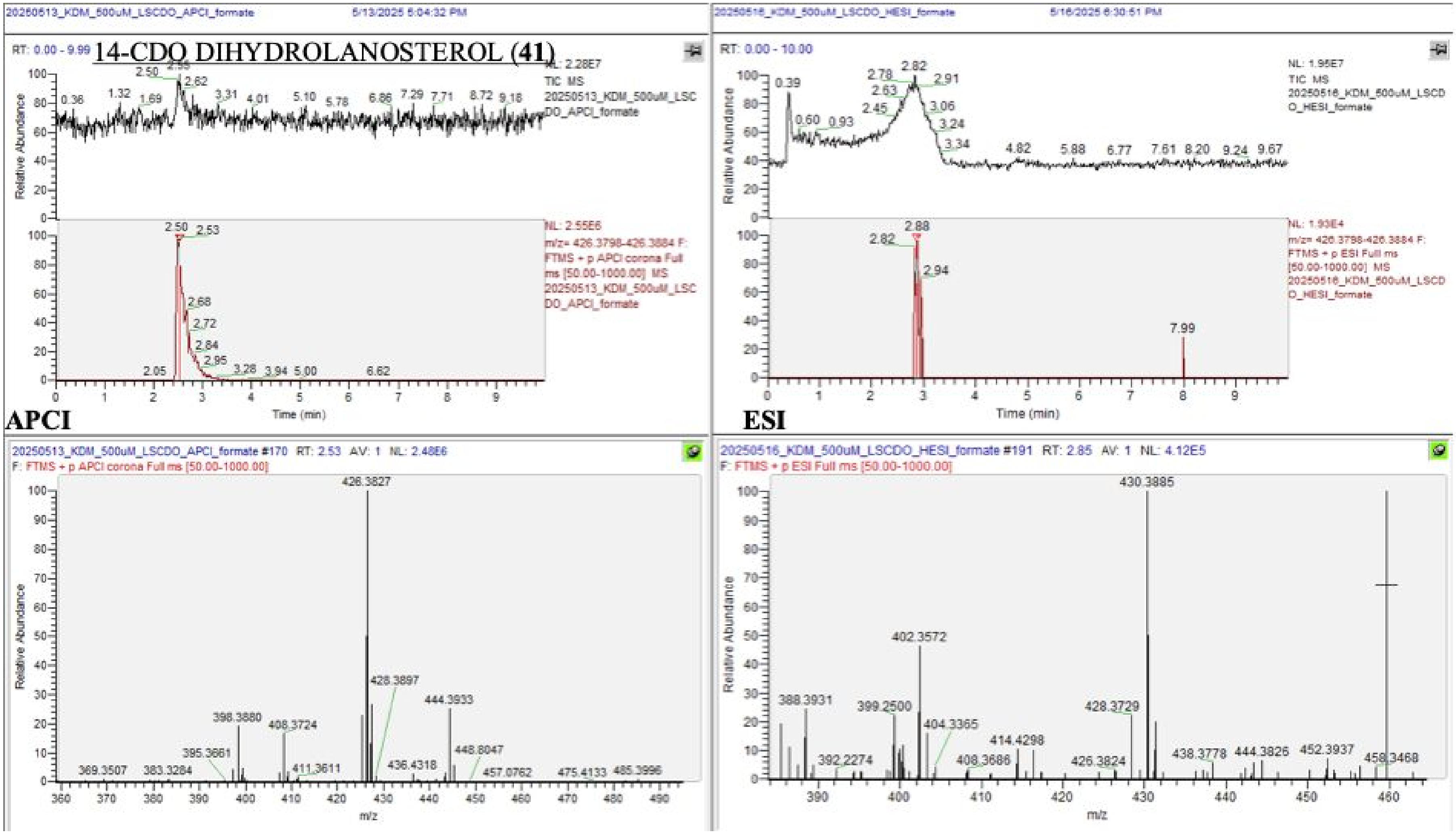

Figure S41. LC-HRMS chromatograms of 14-CDO dihydrolanosterol (**41**, 500 μM) ionized by APCI (left) and HESI (right).

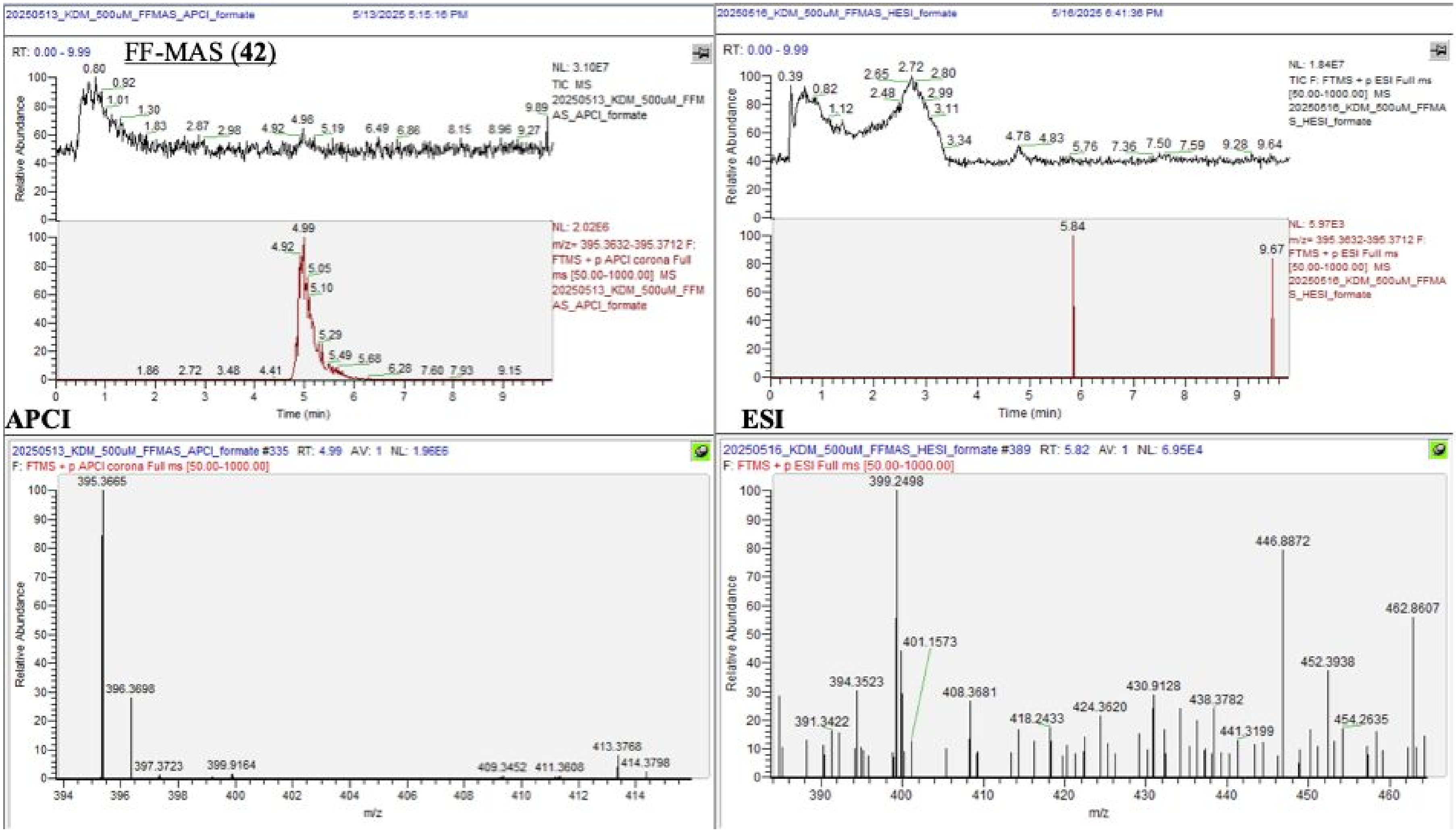

Figure S42. LC-HRMS chromatograms of FF-MAS (**42**, 500 μM) ionized by APCI (left) and HESI (right).

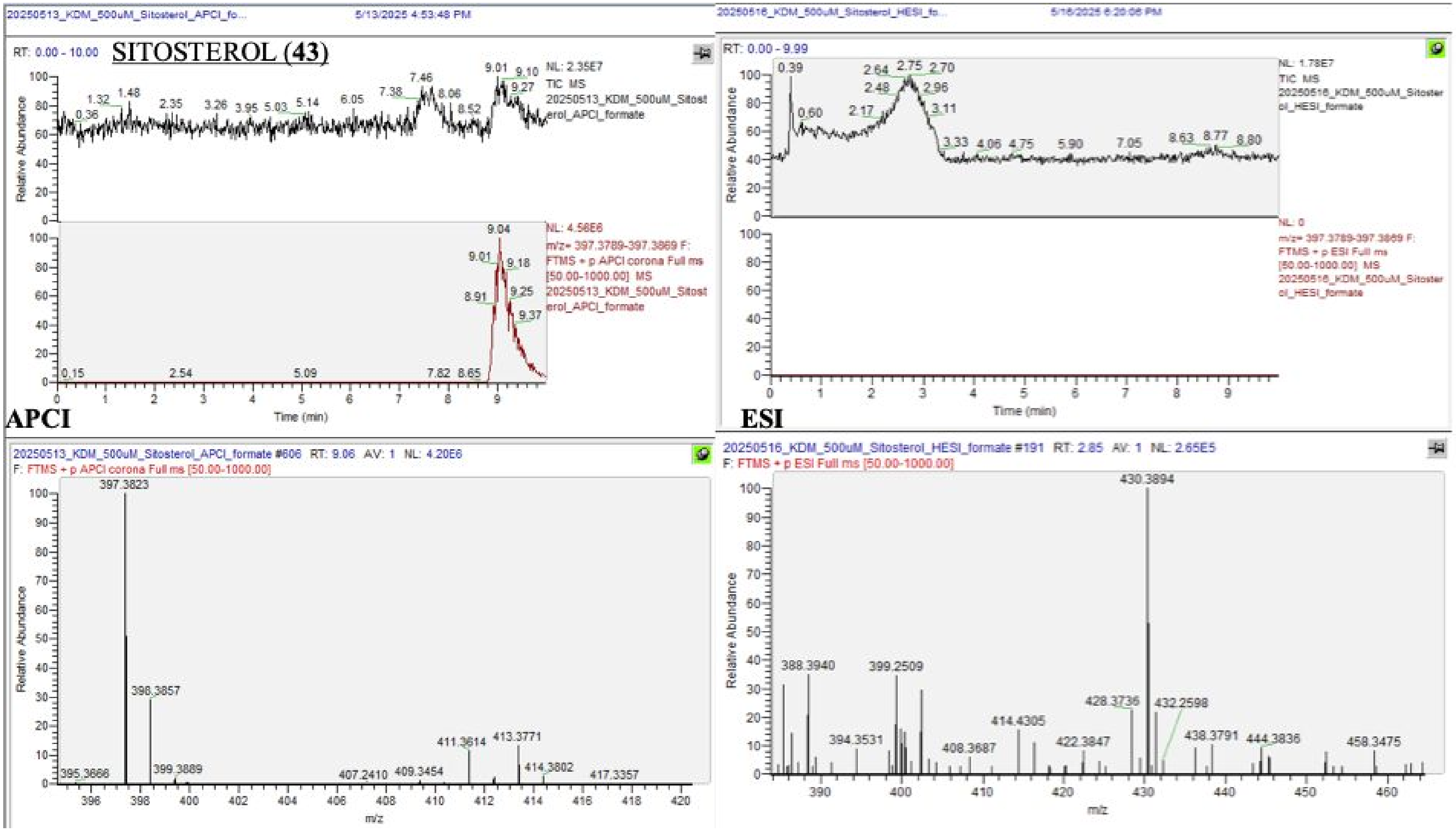

Figure S43. LC-HRMS chromatograms of sitosterol (**43**, 500 μM) ionized by APCI (left) and HESI (right).

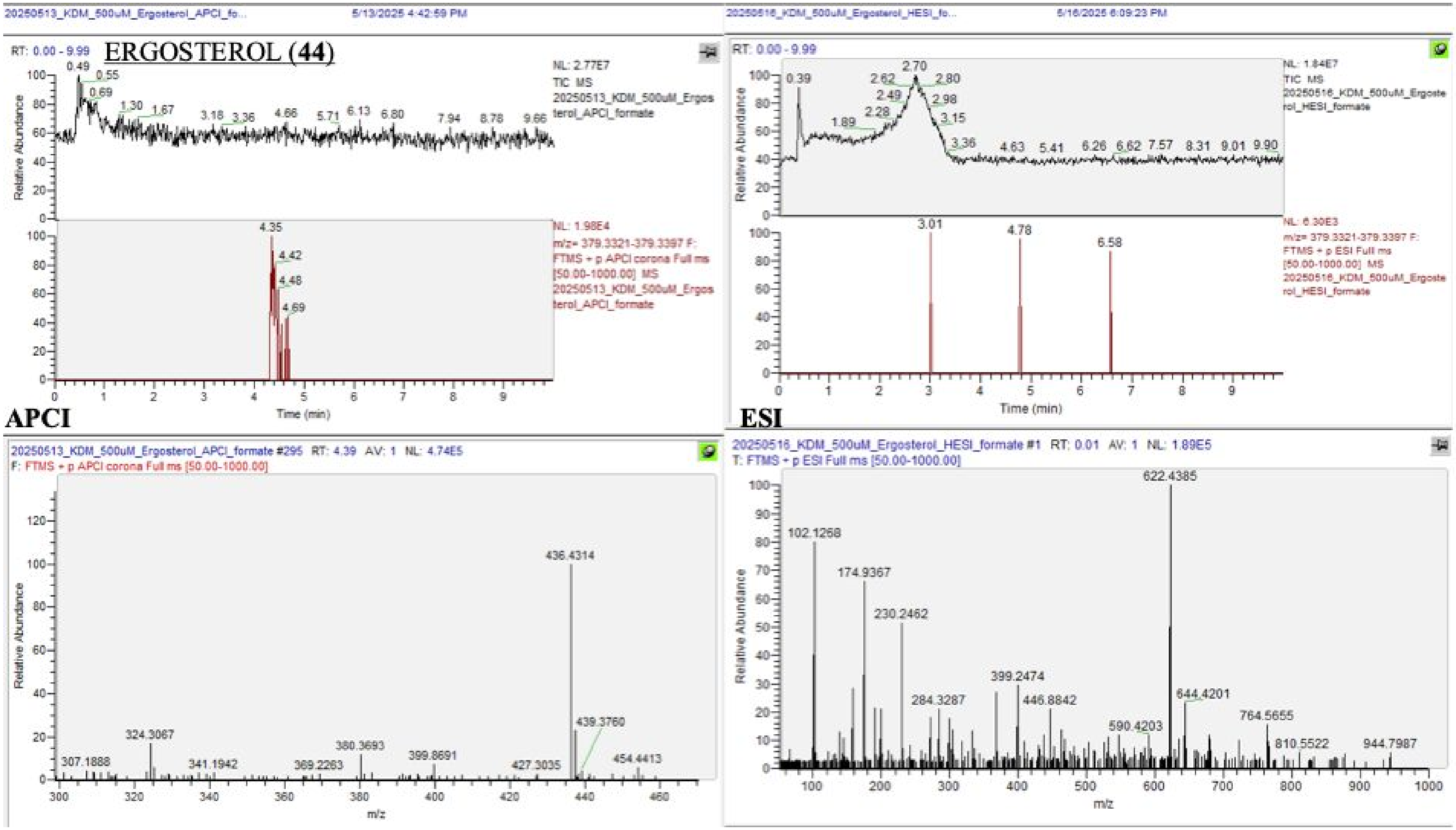

Figure S44. LC-HRMS chromatograms of ergosterol (**44**, 500 µM) ionized by APCI (left) and HESI (right).

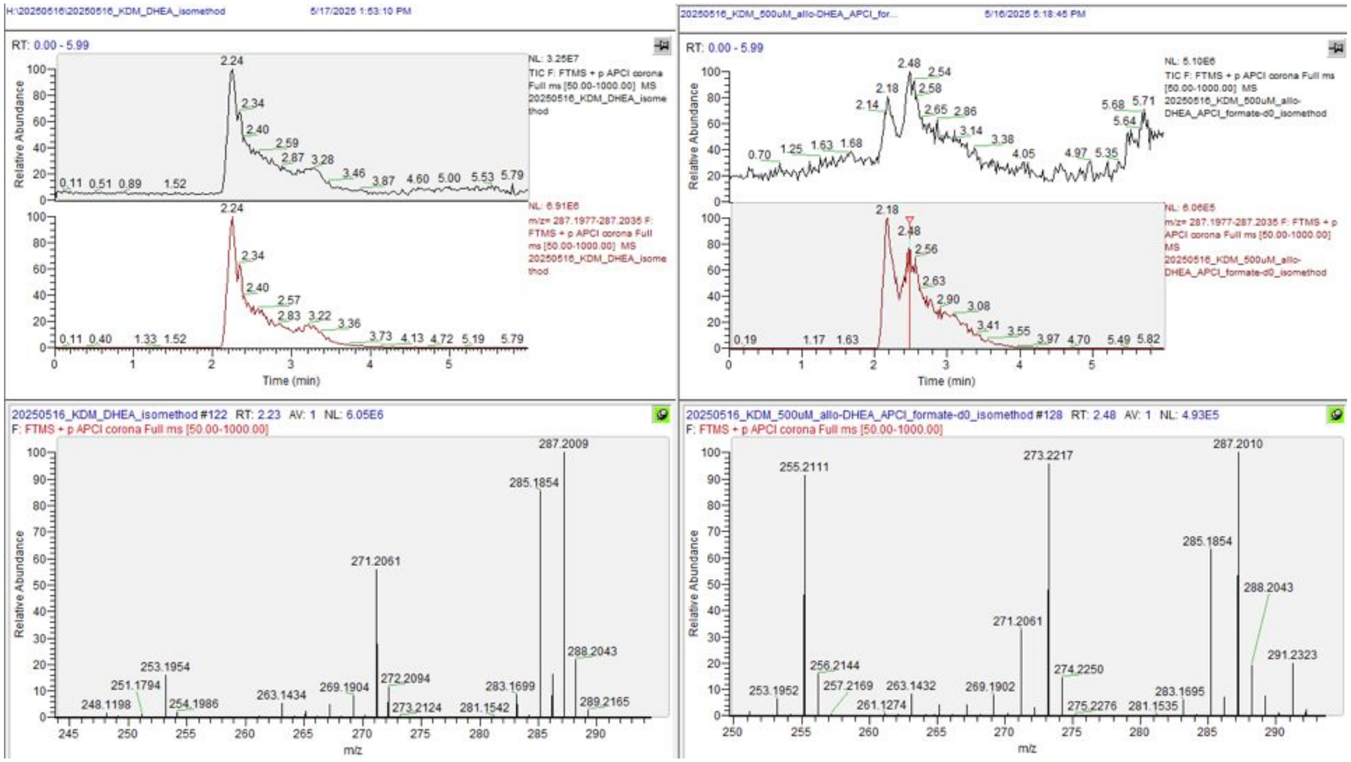

Figure S45. APCI$^+$ analysis of DHEA and *allo*-DHEA. LC-APCI-MS chromatograms (above) and *m/z* spectra (below) of (left) DHEA and (right) allo-DHEA. The base peak (*m/z* 287) is isolated from the TIC.

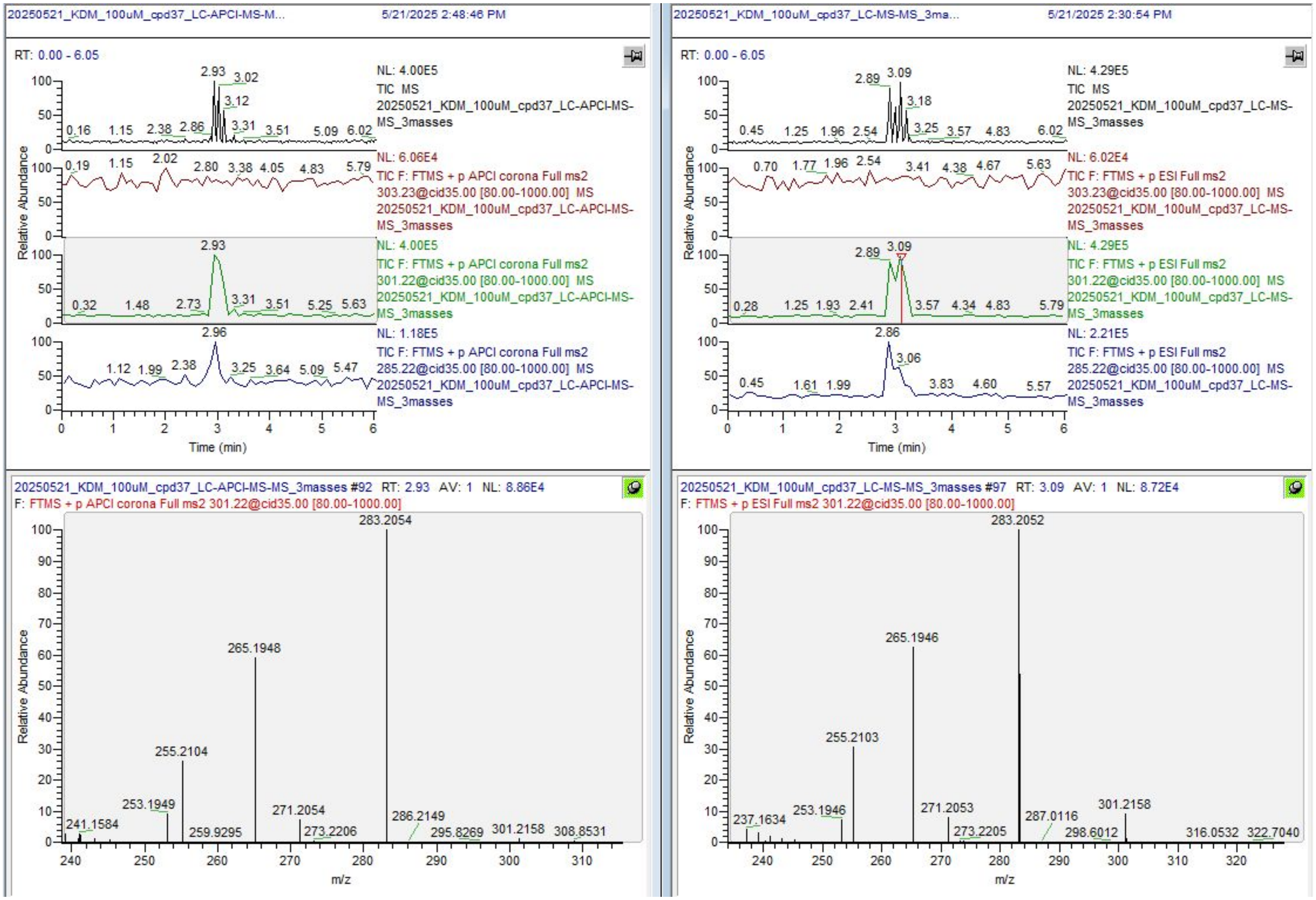

Figure S46. APCI$^+$ and HESI$^+$ targeted LC-MS/MS analysis of **37**. Chromatograms (above) and *m/z* spectra (of isolated *m/z* 301 ion, below) of APCI (left) and HESI (right) MS/MS analyses of compound **37**. Dominant daughter ions include MH^+-H_2O (*m/z* 283) and MH^+-$2H_2O$ (*m/z* 265). The chromatograms of the targeted ions (MH^+ ion (*m/z* 303), MH^+-2 ion (*m/z* 301), and the MH^+-H_2O ion (*m/z* 285)) are displayed.

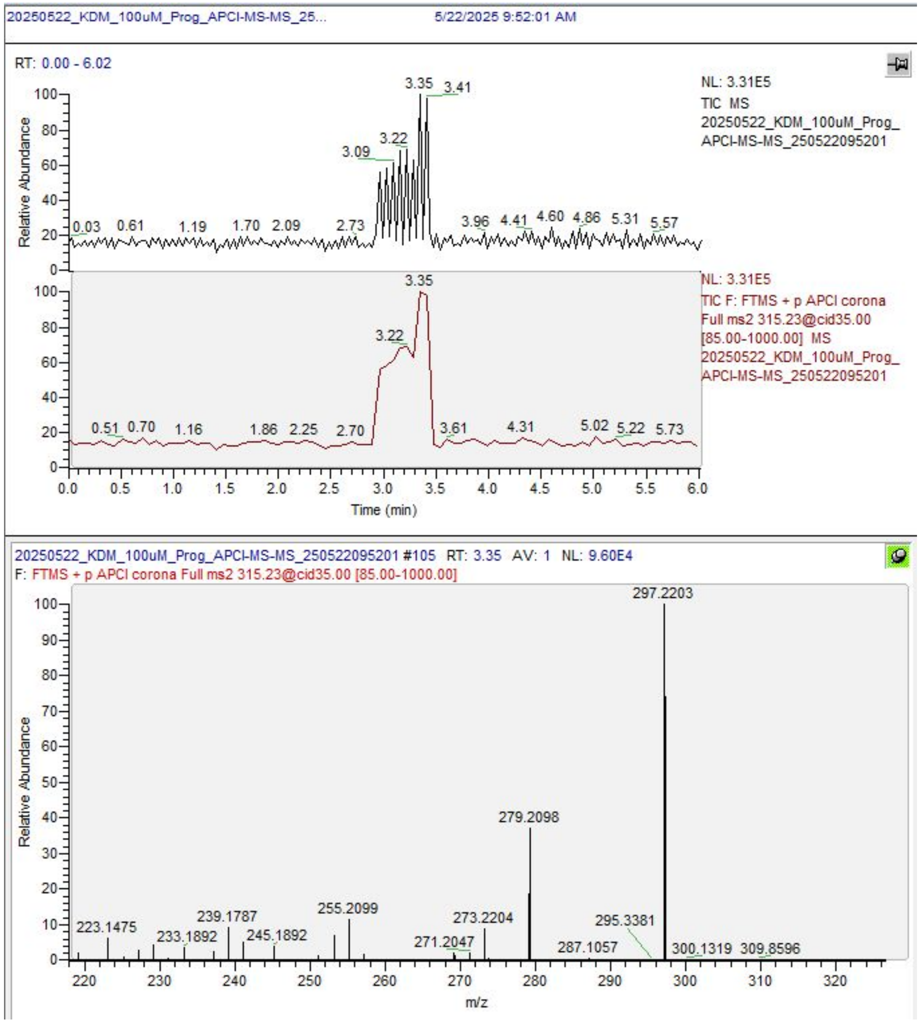

Figure S47. APCI$^+$ targeted LC-MS/MS analysis of **39**. Chromatogram (above) and *m/z* spectrum (of isolated *m/z* 315 ion, below) of APCI MS/MS analyses of compound **39**. Dominant daughter ions include MH^+-H_2O (*m/z* 297) and MH^+-$2H_2O$ (*m/z* 279). The chromatogram of the targeted ion (MH^+ ion (*m/z* 315) is displayed.

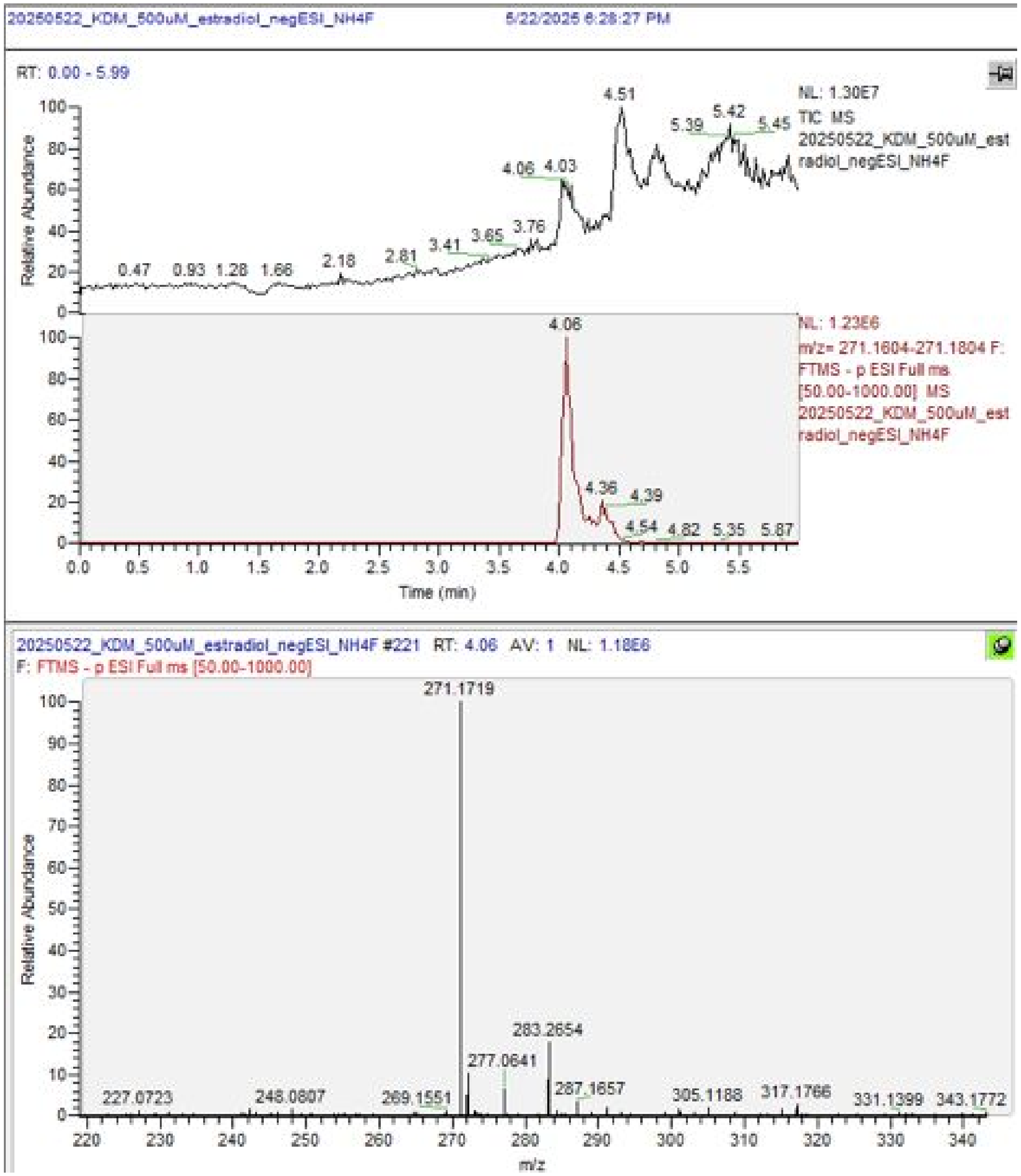

Figure S48. HESI⁻ analysis of **1** with NH_4F mobile phase. The top panel contains the TIC and extracted ion $[M-H]^-$ chromatograms and the bottom panel is the *m/z* spectrum of the $[M-H]^-$ ion.

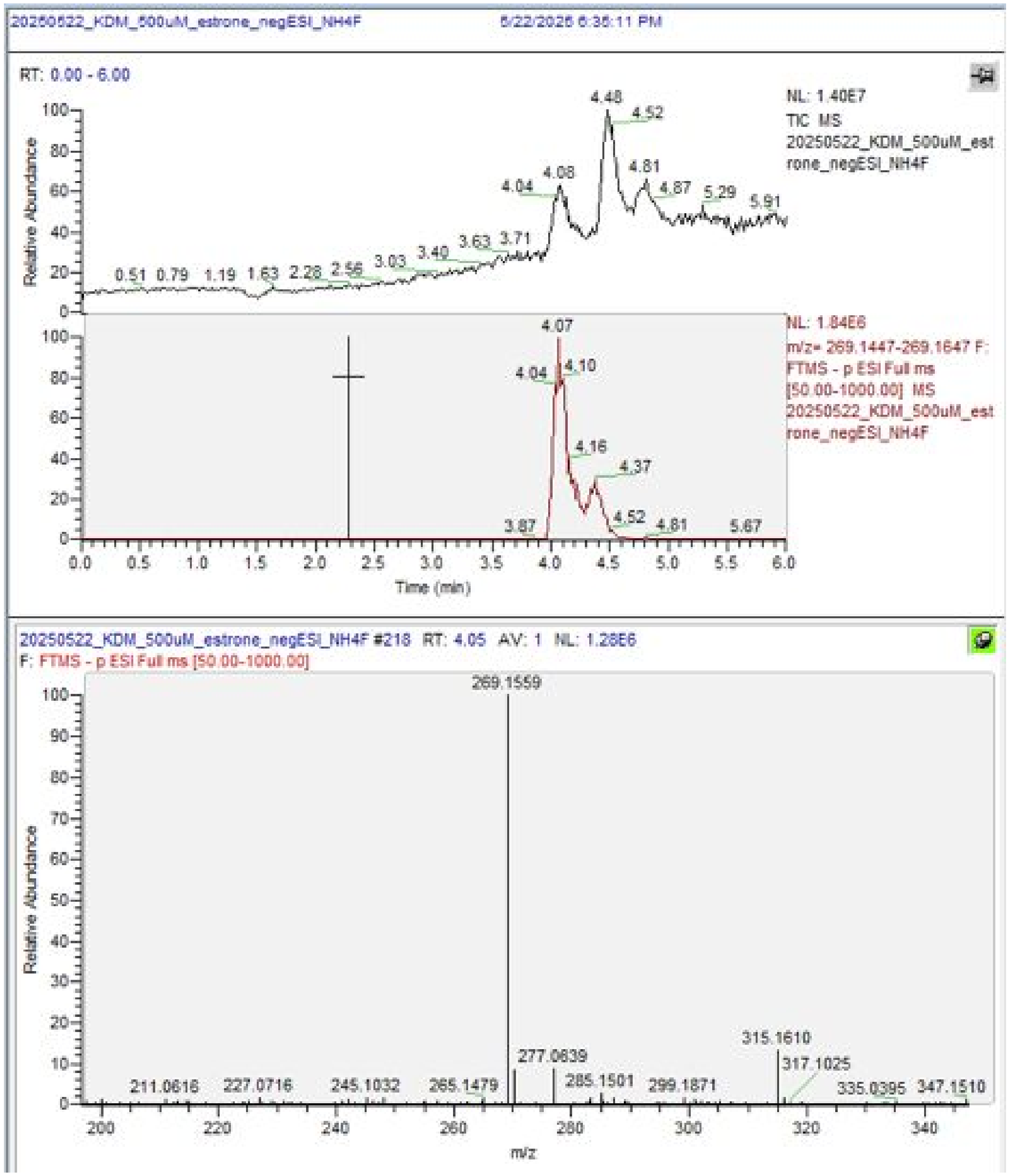

Figure S49. HESI⁻ analysis of **2** with NH_4F mobile phase. The top panel contains the TIC and extracted ion $[M-H]^-$ chromatograms and the bottom panel is the *m/z* spectrum of the $[M-H]^-$ ion.

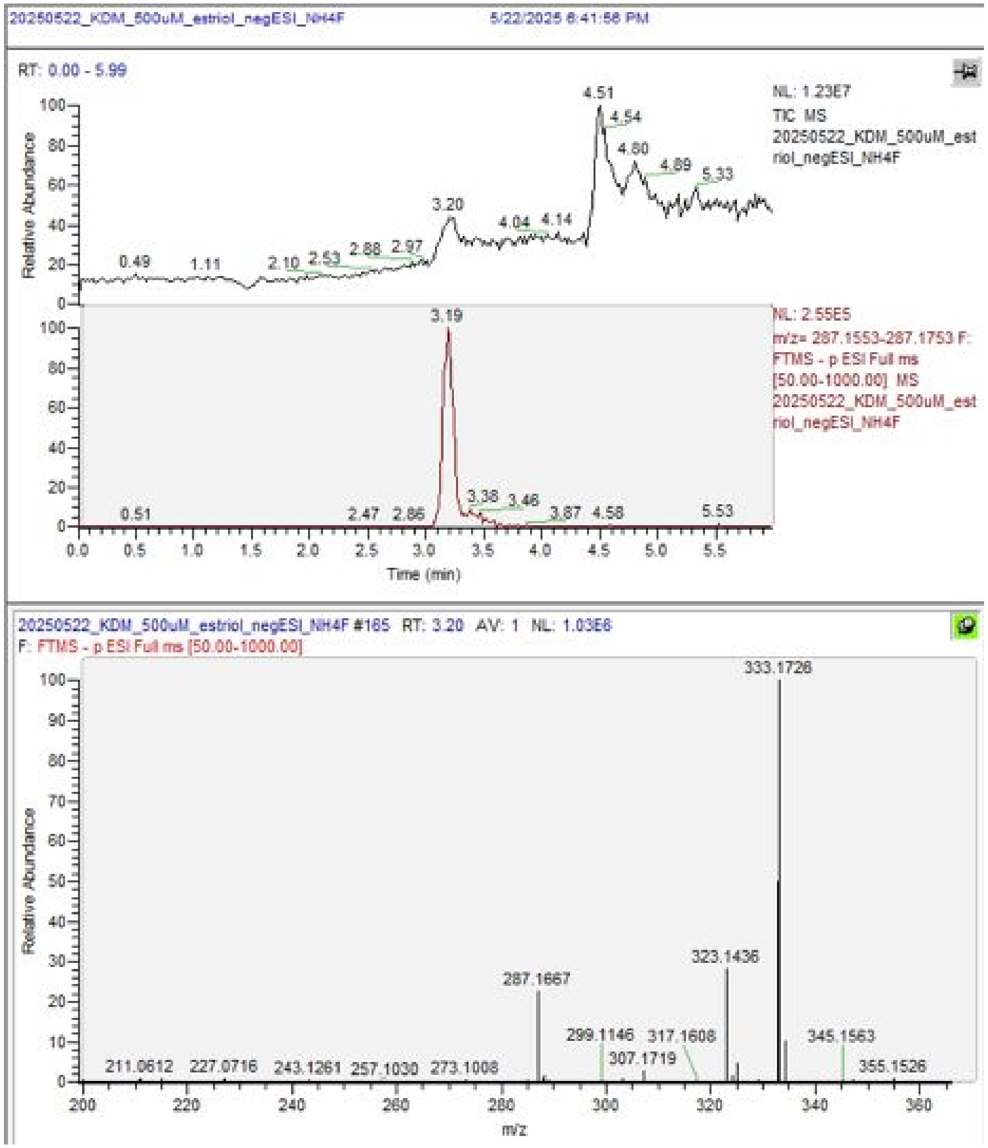

Figure S50. HESI$^-$ analysis of **3** with NH_4F mobile phase. The top panel contains the TIC and extracted ion $[M-H]^-$ chromatograms and the bottom panel is the *m/z* spectrum of the $[M-H]^-$ ion.

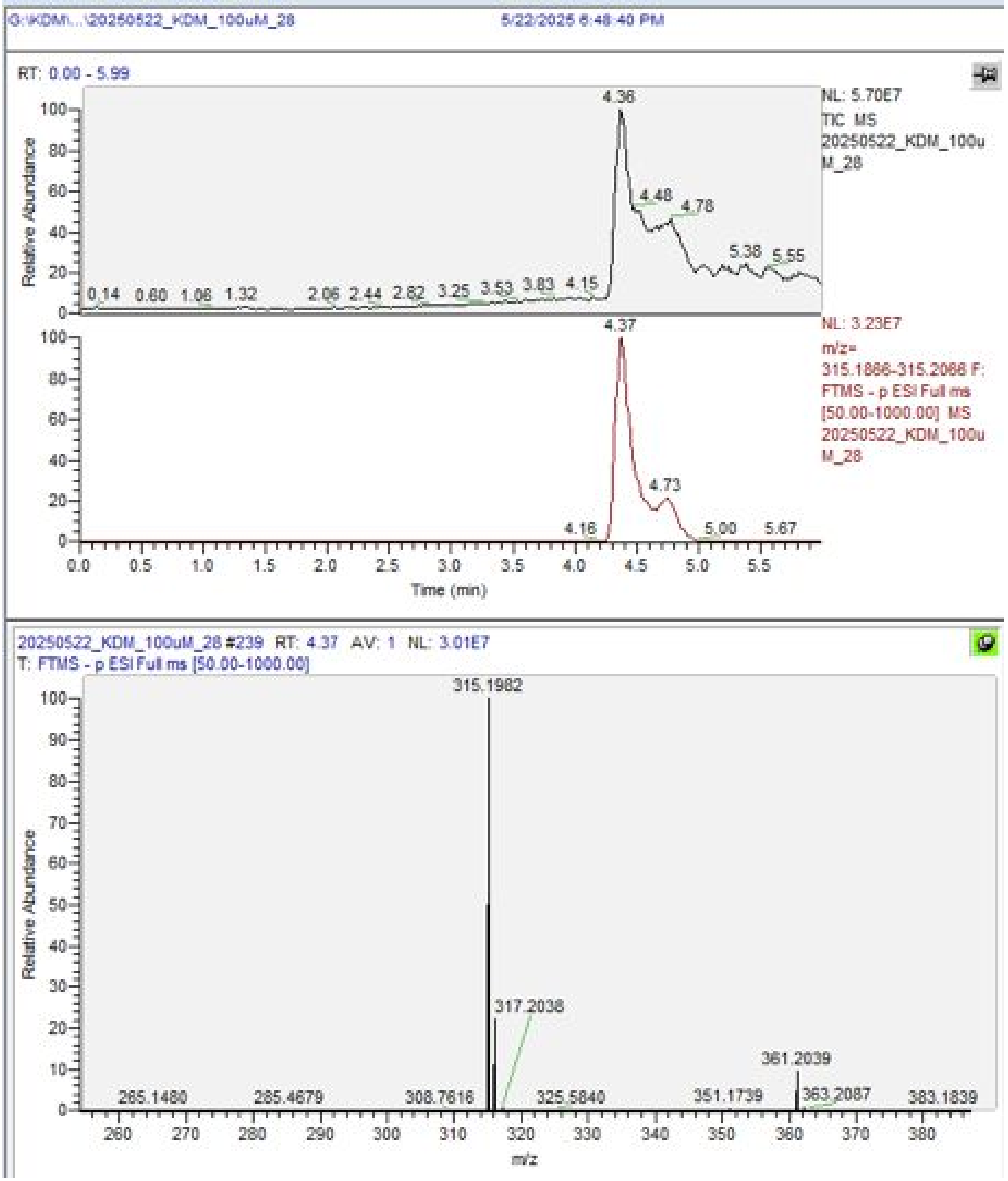

Figure S51. HESI$^-$ analysis of **28** with NH_4F mobile phase. The top panel contains the TIC and extracted ion [M-H]$^-$ chromatograms and the bottom panel is the *m/z* spectrum of the [M-H]$^-$ ion.

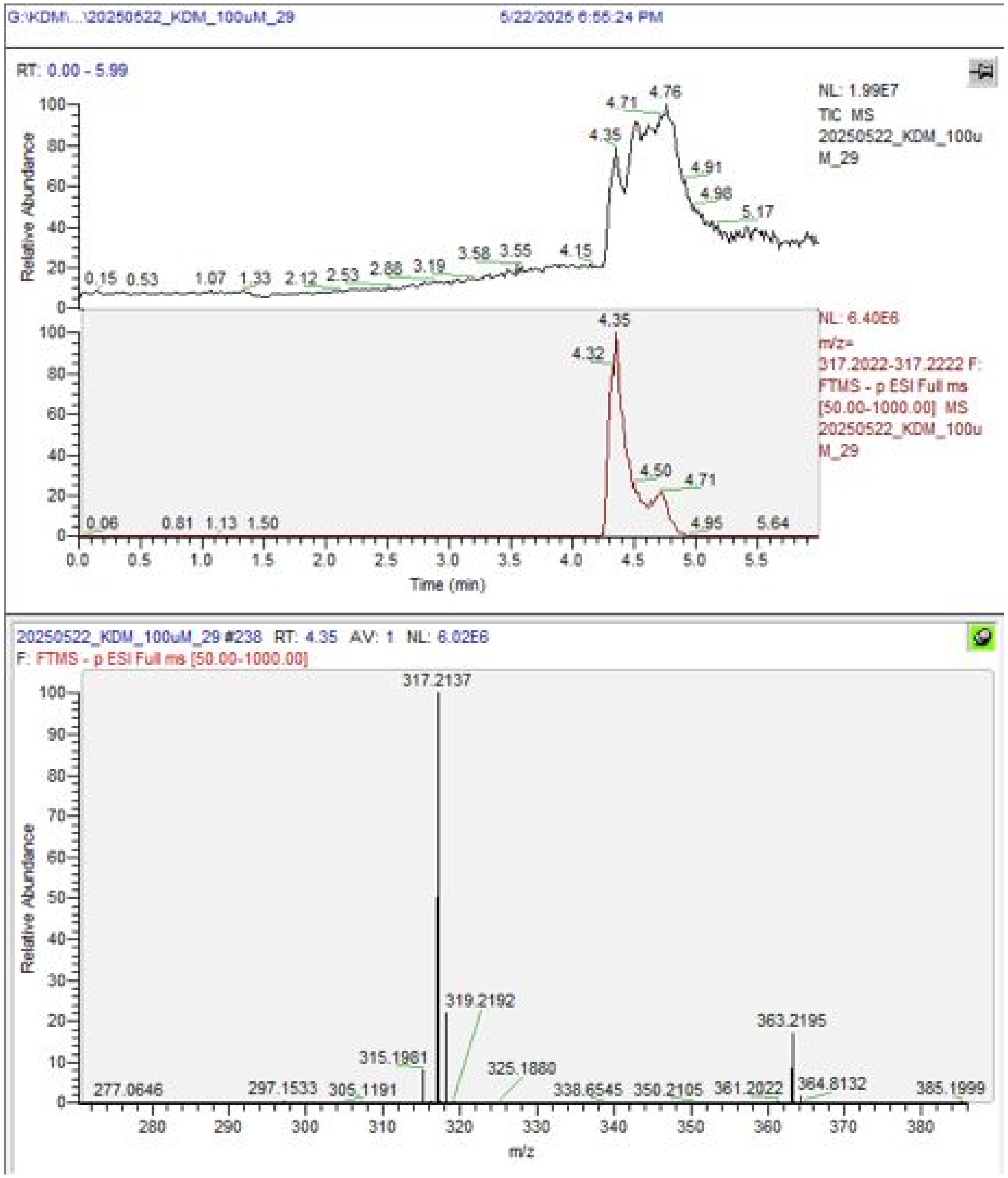

Figure S52. HESI$^-$ analysis of **29** with NH_4F mobile phase. The top panel contains the TIC and extracted ion [M-H]$^-$ chromatograms and the bottom panel is the *m/z* spectrum of the [M-H]$^-$ ion.

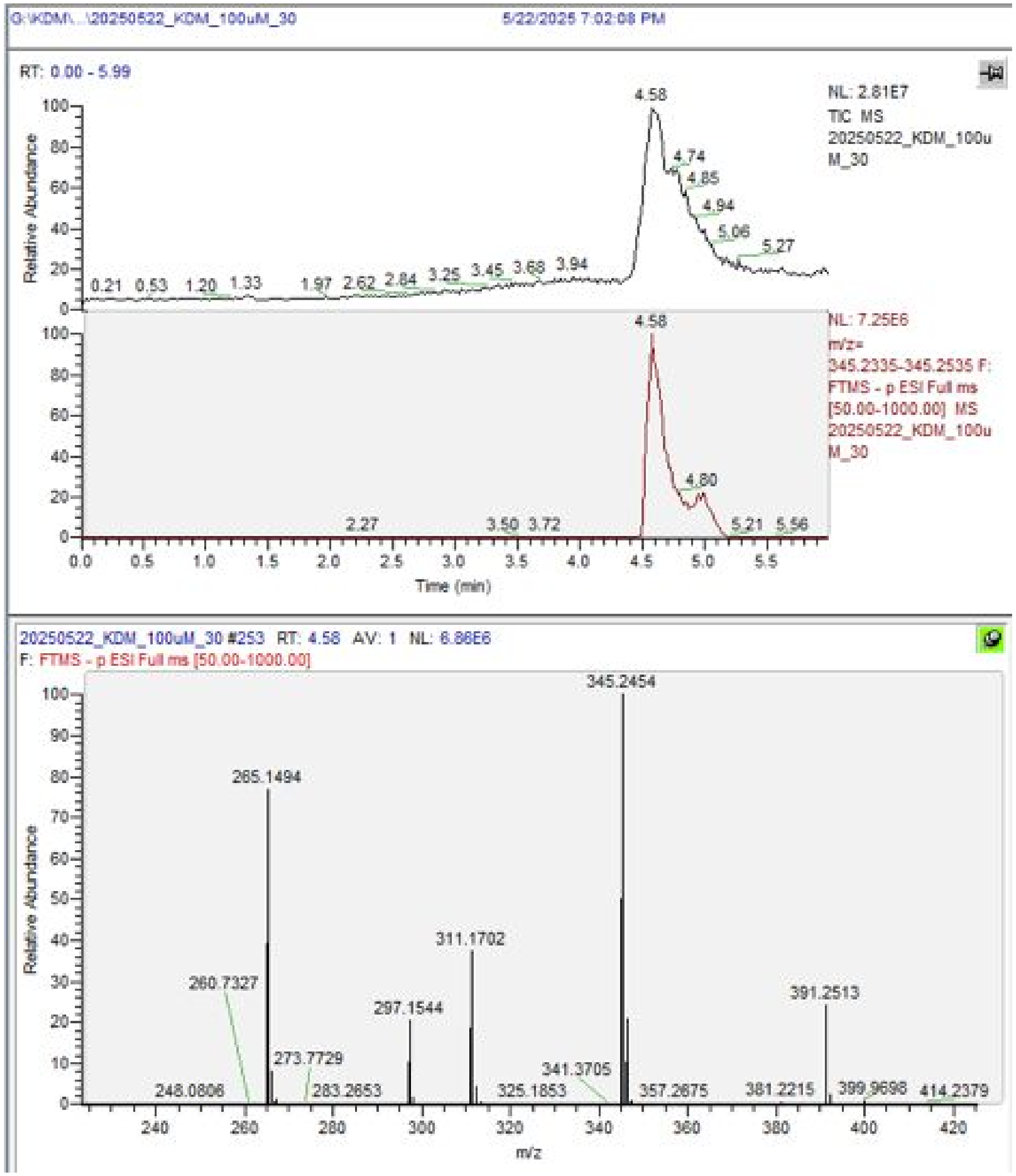

Figure S53. HESI⁻ analysis of **30** with NH_4F mobile phase. The top panel contains the TIC and extracted ion $[M-H]^-$ chromatograms and the bottom panel is the *m/z* spectrum of the $[M-H]^-$ ion.
